# Approval of extreme right-wing organizations and social movements and support for political violence in the United States: findings from a nationally representative survey

**DOI:** 10.1186/s40621-025-00631-8

**Published:** 2025-12-08

**Authors:** Garen J. Wintemute, Bradley Velasquez, Yueju Li, Elizabeth A. Tomsich, Paul M. Reeping, Daniel Tancredi, Sonia L. Robinson

**Affiliations:** 1 Department of Emergency Medicine, School of Medicine, UC Davis, 2221 Stockton Blvd, Sacramento, CA 95814 USA; 2 Violence Prevention Research Program, University of California Davis, Sacramento, CA USA; 3 California Firearm Violence Research Center, UC Davis, Sacramento, CA USA; 4 Department of Public Health Sciences, University of California, Davis, Sacramento, CA USA; 5 Department of Pediatrics, University of California, Davis, Sacramento, CA USA; 6 Present Address: Vital City NYC, 605 West 113th Street, Suite 1 New York, NY 10025 USA

**Keywords:** Political violence, Firearm violence, Civil war, Domestic violent extremism, Authoritarianism, Proud boys, Oath keepers, Three percenters, QAnon, White supremacy, Christian nationalism, Militia, Boogaloo

## Abstract

**Background:**

Concern for political violence in the United States remains high. The limited information available indicates that approval of specific extreme right-wing organizations and social movements is associated with support for and willingness to engage in political violence, but systematic data are lacking. The study objective is to quantify those associations at the individual level in a nationally representative survey sample.

**Methods:**

Cross-sectional analysis from Wave 1 of a nationally representative survey of members of the Ipsos KnowledgePanel, conducted May-June 2022. The exposure is approval (categorized from non-approval to strong approval) of 8 named organizations and social movements, considered individually and together. Principal outcomes are justification for political violence, in general and to advance specific political objectives; willingness to engage in political violence, by type of violence and target population; and expectation of firearm use in political violence. Outcomes are expressed as weighted percentages and adjusted prevalence differences (aPDs, expressed in absolute percentage points (pp)).

**Results:**

The completion rate was 55.8%; there were 8,620 respondents. After weighting, 50.6% of respondents (95% CI 49.4%, 51.7%) were female, and 62.6% (95% CI 61.4%, 63.9%) were white, non-Hispanic; the weighted mean (SD) age was 48.4 (18.0) years. Few respondents (1.4%, 95% CI 1.0%, 1.8%) strongly approved of the organizations and movements considered together. However, strong approvers were much more likely than non-approvers to consider violence usually or always justified to advance specific political objectives and much more willing to engage in political violence; aPDs frequently exceeded 30% points. To advance a political objective, 29.1% (95% CI 18.4%, 39.8%) of strong approvers were very or completely willing to kill someone, and 18.1% (95% CI 8.5%, 27.7%) thought it very or extremely likely that they would shoot someone; prevalences among non-approvers were 0.9% (95% CI 0.5%, 1.2%) and 0.6% (95% CI 0.3%, 0.9%), respectively.

**Conclusions:**

Approval of extreme right-wing organizations and social movements is strongly associated with support for and willingness to engage in political violence. Given continued concern for political violence in the United States and the new possibility that such individuals might join federal law enforcement in large numbers, focused prevention measures are urgently needed.

**Supplementary Information:**

The online version contains supplementary material available at 10.1186/s40621-025-00631-8.

Political violence—the use of physical force to advance political objectives [1]—is increasingly recognized as a public health problem in the United States (US) [2, 3]. In surveys conducted by the authors during 2022–2024, 25%−33% of respondents considered violence to be usually or always justified to advance at least 1 specified political objective [4–6]. Others have found that 24% of firearm purchases in 2023 were “motivated by concerns of political violence” [3]. There is concern among experts that widespread political violence may occur in the US [7–10]. Preventing such violence is important to preserving the public’s health and safety and the nation’s future as a democracy.

Expert assessment in recent years—including by the Federal Bureau of Investigation, Department of Homeland Security, and Director of National Intelligence—has been that the threat of political violence in the US is driven by right-wing extremism [7, 8, 11–13]. Cynthia Miller-Idriss characterizes right-wing extremism (her preferred “best bad term” is “the far right”) as “a spectrum or as a cluster of overlapping ideologies and practices” that are “antidemocratic, antiegalitarian, white supremacist, and embedded in solutions like authoritarianism, ethnic cleansing or ethnic migration, and the establishment of separate ethno- states or enclaves along racial and ethnic lines” [14].

Evidence supporting the assessment linking right-wing extremism to violence extends well beyond case reports to include a substantial body of research [14–23]. Just prior to the COVID pandemic, for example, more than 80% of homicides related to extremist violence in the US were attributable to right-wing extremism [14]. In a 2022 analysis of more than 1500 extremist events in the US, right-wing extremists were twice as likely as left-wing extremists to resort to violence [16].

Specific right-wing extremist organizations and social movements such as the Proud Boys, Oath Keepers, Three Percenters, QAnon, the white supremacy movement, the Christian nationalist movement, the militia movement, and the boogaloo movement have repeatedly been linked to political violence [11, 14, 17–26], including the riot at the Capitol on January 6, 2021 [10]. One expert described that event as “the first wave of Christian nationalist violence” [27]. Among the Proud Boys, committing an act of violence is required to attain full membership [19].

Prosecutions for involvement in the January 6 riot significantly impacted the Oath Keepers and Proud Boys [28, 29]. In 2025, however, the Proud Boys are regrouping and adding members [30, 31]. On his first day in office, President Trump pardoned or commuted the sentences of the nearly 1,600 people charged with or convicted of offenses related to January 6 [32]. Even before the 2024 election, there was concern over the potential development of “an army of modern blackshirts” [33] that could be deployed in President Trump’s support, such as to implement a mass deportation program [33–36]. That concern has increased with the May 2025 announcement by the White House of Project Homecoming, which allows the Department of Homeland Security to deputize thousands of private individuals for that purpose [37, 38]. Funding for that expansion was included in H.R. 1, which was signed into law July 4, 2025 [39].

Unfortunately, there are significant gaps in our knowledge of right-wing extremism and its potential for violence. We know relatively little about the number of people actively involved, for example; estimates range from 6,000 to 35,000 (roughly 0.002% to 0.013% of the adult population) for the Proud Boys as of 2022 [18] and from 75,000 to 100,000 (roughly 0.029% to 0.038% of the adult population) for the white supremacy movement as of 2020 [14]. Approval of right-wing extremism may be much more common [40]; this is particularly important given suggestive evidence [41] that “right-wing ideological values” are associated with support for political violence [42]. There are pervasive limitations to studies of right-wing violence, as well. These have often been based on small, non-probability samples; have focused on members of just 1 extremist organization or movement; have not included comparison populations; have not examined associations between membership (or, more broadly, affiliation) and political violence; have not defined political violence or included specific political violence outcome measures; or have not assessed personal willingness to commit political violence.

This study aims to address those gaps. It relies on 2022 data from a large, nationally representative longitudinal survey [4, 43–45]. It assesses the extent to which respondents’ approval (versus non-approval) of the 8 organizations and social movements named above, collectively and individually, is associated with a broad array of political violence outcome measures (using specifically-defined terms in place of “political violence” in the survey instrument). These include respondents’ opinion that political violence is justified, in general and to advance each of a list of specific political objectives; respondents’ willingness to commit political violence across specified types of violence and against specified target populations; and respondents’ predicted use of firearms in acts of political violence. A deeper understanding of these matters can facilitate the design and implementation of prevention measures.

## Methods

The survey was designed by the authors and administered online in English and Spanish from May 13 to June 2, 2022 by the research firm Ipsos [46]. The study was reviewed by the UC Davis Institutional Review Board (protocol 187125: exempt from full review, category 2, survey research). Before participants accessed the questionnaire, they were provided informed consent language that concluded, “[by] continuing, you are agreeing to participate in this study.” This report follows American Association for Public Opinion Research guidelines [47].

## Participants

Respondents were drawn from the Ipsos KnowledgePanel, an online research panel widely used in studies of violence and firearm ownership [48–51]. To establish national representativeness, members are recruited through address-based probability sampling using the US Postal Service’s Delivery Sequence File [52, 53]. Recruitment involves repeated contact attempts, if necessary, by mail and telephone. Recruited households without internet access are provided a web-enabled device and free internet service. A modest, primarily point-based incentive program seeks to encourage participation and promote participant retention.

A probability-proportional-to-size procedure was used to select a study-specific representative sample. All KnowledgePanel members aged ≥ 18 years were eligible. E-mail invitations were followed by automatic e-mail and telephone reminders to non-respondents beginning 3 days later [52, 53].

A final survey weight provided by Ipsos adjusted for the initial probability of selection into KnowledgePanel and for survey-specific nonresponse and over- or under-coverage using design weights with post-stratification raking ratio adjustments. The weighted sample was designed to be statistically representative of the noninstitutionalized adult population of the US as reflected in the 2021 March supplement of the Current Population Survey [52, 53].

### Measures

Sociodemographic data were collected by Ipsos from KnowledgePanel member profiles.

Eight survey items concerned the principal exposure for this analysis: approval of the 8 specified extreme right-wing organizations and social movements named previously. They were chosen by the authors based on the expectation that they were well known to respondents, either because of their size and longstanding existence (examples: the Christian nationalist movement, the white supremacist movement) or their recent salience in national politics (examples: the Proud Boys, the militia movement).

Our outcome measures concerned political violence; we included non-political violence to assess the specificity of any association between political violence and the exposure under study. Violence was represented in the questionnaire by “force or violence,” defined as “physical force strong enough that it could cause pain or injury to a person.” “Force or violence to advance an important political objective that you support” was used to describe political violence.

Respondents were asked first about nonpolitical violence and then about the extent to which they considered political violence to be justified “in general” and to advance 17 specified political objectives (examples: “to return Donald Trump to the presidency this year,” “to stop police violence”). Nine objectives were presented to all respondents and 8 were paired, each respondent seeing 1 item from each pair. Each respondent was presented with 13 of 17 objectives.

Respondents who considered violence at least sometimes justified for at least 1 objective were asked about their personal willingness to engage in violence, by type of violence (to “damage property,” “threaten or intimidate a person,” “injure a person,” “kill a person”) and target population (examples: “an elected federal or state government official,” “a police officer,” “a person who does not share your religion”).

All respondents were asked about the future likelihood of their use of firearms in a situation where they considered political violence to be justified (examples: “I will be armed with a gun,” “I will shoot someone with a gun”).

To provide context for findings on their beliefs regarding political violence, respondents were also presented items that elicited their beliefs regarding the status and stature of democracy and the prospect of civil war.

The full text of questionnaire items is in the Supplement (see Supplement, Additional File 1). Except where noted otherwise there, questionnaire items were developed by the authors.

## Implementation

Forty KnowledgePanel members participated in an English language pretest administered April 27 to May 2, 2022.

Respondents were randomized 1:1 to receive response options in order from negative to positive valence (e.g., from ‘do not agree’ to ‘strongly agree’) or the reverse. When a question presented multiple items, their order was randomized unless ordering was necessary. Logic-driving questions (those for which responses might invoke a skip pattern) included non-response prompts. Other design elements were adopted to minimize inattentive responses and biased estimation of support for political violence (see Supplement, Additional file 1).

We employed unipolar response arrays without a neutral midpoint. The literature is not in agreement on the use of neutral midpoints [54, 55]. We were persuaded by the studies reviewed by Chyung et al. [54], which suggest that neutral midpoints facilitate satisficing—selection of “a minimally acceptable response as soon as it is found.” Satisficing is particularly common when respondents are uncomfortable with a topic or under social desirability pressures [54], and both conditions apply here. Our analyses focus on affirmative responses above the “somewhat” or “sometimes” level to minimize satisficing’s impact on the results.

### Statistical analysis

Analyses were conducted in SAS version 9.4 (SAS Institute, Inc., Cary, NC) and R version 4.3.0 (R Project for Statistical Computing).

Individual exposure item responses were coded ordinally (do not approve = 0, somewhat approve = 1, strongly approve = 2, very strongly approve = 3) and summed for each respondent for each exposure. Summed scores were normalized to a range between 0 and 1, which represented the minimum and maximum theoretically possible scores. Normalized scores were then categorized according to their position on that range (strong approval, normalized score > 0.66… and ≤ 1; moderate approval, normalized score > 0.33… and ≤ 0.66; weak approval, normalized score > 0… and ≤ 0.33; non-approval, normalized score = 0).

Modified procedures allowed for inclusion of respondents who did not report approval for up to half the organizations and movements (see Supplement, Additional file 1). This resulted almost entirely from respondents indicating that they had never heard of an organization or movement or did not know enough about an organization or movement to rate it (see Supplement, Additional file 1, Table S1). Actual non-response for these items did not exceed 2%.

We calculated Cronbach’s α for a combined measure of approval based on findings for individual organizations and movements, with bootstrapped 95% confidence intervals (CI) based on 500 samples.

To generate prevalence estimates, we calculated unadjusted and adjusted weighted percentages and 95% CIs using the *survey* and *srvyr* packages in R. We defined outcomes dichotomously and employed robust standard errors to correct for design effects and heteroskedasticity in binary outcomes. We considered several models for calculating adjusted prevalence differences (aPDs; see Supplement, Additional File 1), choosing the final model based on concordance with theory, findings from prior research, and fit statistics. The final model included age, race and ethnicity, gender, education, income, Census division, and rurality. APDs were expressed in absolute percentage points (pp) with 95% CIs. P-values were corrected for multiple comparisons by controlling the false discovery rate (FDR) using the Benjamini-Hochberg method [56] and presented as q-values [57].

In secondary analyses, we assessed associations between approval of these organizations and support for non-political violence, beliefs and attitudes on democracy and civil war, and the association between approval of each individual organization or movement and our political violence outcome measures.

## Results

Of 15,449 panel members invited, 8,620 completed the survey, yielding a 55.8% completion rate. After weighting, 50.6% of respondents (95% CI 49.4%, 51.7%) were female, and 62.6% (95% CI 61.4%, 63.9%) were white, non-Hispanic; the weighted mean (SD) age was 48.4 (18.0) years (Supplement, Additional file 1, Table S2). Compared with nonrespondents, respondents were older and more frequently white, non-Hispanic; were more often married; had higher education and income; and were less likely to be working (Supplement, Additional file 1, Table S3).

Cronbach’s α for the combined measure of approval based on findings for individual organizations and movements was 0.87 (95% CI 0.82, 0.91). Weighted prevalences were: strong approval, 1.4% (*n* = 77; 95% CI 1.0%, 1.8%); moderate approval, 3.3% (*n* = 247; 95% CI 2.8%, 3.8%); weak approval, 8.6% (*n* = 778; 95% CI 7.9%, 9.2%); non-approval 39.5% (*n* = 3724; 95% CI 38.4%, 40.7%). Another 3794 respondents did not report their approval for more than half the organizations and movements (almost always because they had not heard of them or did not know them well enough to rate them; see Supplement, Additional file 1, Table S1) and could not be categorized.

## Political violence

The view that violence to advance political objectives “in general” was usually or always justified was much more common among strong approvers than among non-approvers (aPD 40.7pp, 95% CI 28.1pp, 53.3pp; q < 0.001), as was the belief that violence was usually or always justified to advance at least 1 of 17 specific political objectives (aPD 57.0pp, 95% CI 47.1pp, 66.9pp; q < 0.001) (Table 1).

**Table 1:** Approval of specified organizations and movements and justification for political violence

What do you think about the use of force or violence in the following situations?	Approval of Organizations and Movements			
	Non-Approval		Weak Approval	
	Unweighted *n*	Weighted % (95% CI)	Unweighted *n*	Weighted % (95% CI)
In general…to advance an important political objective that you support				
Never justified	3184	83.0 (81.5,84.4)	620	77.4 (74.0,80.8)
Sometimes justified	511	16.0 (14.6,17.4)	133	18.2 (15.2,21.3)
Usually or always justified	25	1.0 (0.5,1.4)	25	4.4 (2.5,6.2)
Adjusted prevalence difference* (95% CI; q-value)	Referent		3.1 (1.2,5.0; 0.01)	
Usually or always justified to advance at least 1 of 17 objectives				
	756	20.7 (19.2,22.2)	379	49.0 (45.0,52.9)
Adjusted prevalence difference* (95% CI; q-value)	Referent		24.8 (20.5,29.1; <0.001)	
To return Donald Trump to the presidency this year				
Never justified	3608	96.7 (96.0,97.4)	653	83.0 (79.9,86.2)
Sometimes justified	40	1.2 (0.8,1.6)	59	7.8 (5.7,9.9)
Usually or always justified	56	1.6 (1.1,2.1)	58	8.3 (5.9,10.7)
Adjusted prevalence difference* (95% CI; q-value)	Referent		5.8 (3.5,8.1; <0.001)	
To stop an election from being stolen				
Never justified	3163	84.9 (83.6,86.2)	512	65.1 (61.4,68.9)
Sometimes justified	421	11.1 (10.0,12.3)	148	19.4 (16.3,22.5)
Usually or always justified	116	3.3 (2.6,4.0)	115	15.1 (12.2,18.1)
Adjusted prevalence difference* (95% CI; q-value)	Referent		10.2 (7.3,13.1; <0.001)	
To stop people who do not share my beliefs from voting				
Never justified	3636	97.2 (96.5,97.8)	721	91.9 (89.6,94.1)
Sometimes justified	46	1.6 (1.1,2.1)	34	4.8 (3.1,6.4)
Usually or always justified	28	1.0 (0.6,1.4)	21	3.2 (1.7,4.8)
Adjusted prevalence difference* (95% CI; q-value)	Referent		2.1 (0.5,3.8; 0.05)	
To prevent discrimination based on race or ethnicity				
Never justified	2516	65.7 (64.0,67.4)	464	57.8 (53.9,61.7)
Sometimes justified	979	27.3 (25.7,28.9)	200	26.3 (22.8,29.7)
Usually or always justified	206	6.4 (5.5,7.4)	109	15.4 (12.4,18.4)
Adjusted prevalence difference* (95% CI; q-value)	Referent		7.6 (4.5,10.6; <0.001)	
To preserve an American way of life based on Western European traditions				
Never justified	3193	86.8 (85.7,88.0)	476	62.3 (58.5,66.1)
Sometimes justified	431	10.4 (9.4,11.5)	236	29.7 (26.2,33.3)
Usually or always justified	76	2.2 (1.6,2.7)	58	7.2 (5.2,9.2)
Adjusted prevalence difference* (95% CI; q-value)	Referent		4.1 (2.0,6.2; <0.001)	
To preserve the American way of life I believe in				
Never justified	2525	68.9 (67.2,70.5)	307	41.1 (37.2,45.1)
Sometimes justified	984	25.5 (24.0,27.1)	297	37.2 (33.5,41.0)
Usually or always justified	200	5.3 (4.5,6.1)	174	21.6 (18.4,24.9)
Adjusted prevalence difference* (95% CI; q-value)	Referent		14.4 (11.1,17.7; <0.001)	
To oppose Americans who do not share my beliefs				
Never justified	3538	94.0 (93.0,94.9)	668	85.0 (82.2,87.8)
Sometimes justified	150	4.9 (4.0,5.7)	86	11.0 (8.6,13.4)
Usually or always justified	26	1.0 (0.5,1.5)	24	4.0 (2.3,5.6)
Adjusted prevalence difference* (95% CI; q-value)	Referent		2.7 (0.9,4.4; 0.03)	
To oppose the government when it does not share my beliefs				
Never justified	3322	87.4 (86.1,88.7)	579	73.4 (69.9,76.9)
Sometimes justified	341	10.8 (9.6,12.1)	160	21.6 (18.4,24.9)
Usually or always justified	41	1.1 (0.7,1.5)	37	4.9 (3.2,6.6)
Adjusted prevalence difference* (95% CI; q-value)	Referent		3.8 (2.0,5.6; <0.001)	
To oppose the government when it tries to take private land for public purpose				
Never justified	2792	73.8 (72.2,75.4)	368	46.9 (43.0,50.9)
Sometimes justified	775	21.4 (19.9,22.9)	286	36.7 (32.9,40.5)
Usually or always justified	130	4.1 (3.3,4.8)	119	15.9 (13.0,18.8)
Adjusted prevalence difference* (95% CI; q-value)	Referent		10.3 (7.3,13.2; <0.001)	

**What do you think about the use of force or violence in the following situations?**	**Approval of Organizations and Movements**			
	**Moderate Approval**		**Strong Approval**	
	**Unweighted n**	**Weighted % (95% CI)**	**Unweighted n**	**Weighted % (95% CI)**
In general…to advance an important political objective that you support				
Never justified	151	57.1 (49.8,64.5)	20	22.1 (12.0,32.2)
Sometimes justified	74	30.0 (23.4,36.7)	26	33.5 (21.4,45.6)
Usually or always justified	22	12.9 (7.0,18.7)	31	44.4 (31.5,57.2)
Adjusted prevalence difference* (95% CI; q-value)	10.4 (4.9,15.9; 0.002)		40.7 (28.1,53.3; <0.001)	
Usually or always justified to advance at least 1 of 17 objectives				
	164	65.6 (58.6,72.6)	65	83.9 (74.4,93.5)
Adjusted prevalence difference* (95% CI; q-value)	39.6 (32.2,46.9; <0.001)		57.0 (47.1,66.9; <0.001)	
To return Donald Trump to the presidency this year				
Never justified	165	63.4 (56.1,70.6)	22	30.2 (18.2,42.1)
Sometimes justified	38	17.5 (11.6,23.3)	19	22.9 (12.1,33.8)
Usually or always justified	43	18.7 (12.8,24.6)	35	44.7 (32.0,57.4)
Adjusted prevalence difference* (95% CI; q-value)	15.9 (10.1,21.6; <0.001)		42.3 (29.8,54.8; <0.001)	
To stop an election from being stolen				
Never justified	100	40.8 (33.7,48.0)	17	26.4 (14.8,38.0)
Sometimes justified	77	29.4 (23.0,35.8)	20	27.2 (15.2,39.2)
Usually or always justified	68	29.0 (22.2,35.8)	39	44.1 (31.6,56.7)
Adjusted prevalence difference* (95% CI; q-value)	24.3 (17.6,31.0; <0.001)		40.1 (27.5,52.6; <0.001)	
To stop people who do not share my beliefs from voting				
Never justified	185	70.0 (63.1,76.9)	33	36.6 (24.6,48.6)
Sometimes justified	38	17.5 (11.9,23.1)	14	21.1 (10.3,32.0)
Usually or always justified	23	12.0 (6.8,17.1)	29	40.0 (27.4,52.7)
Adjusted prevalence difference* (95% CI; q-value)	9.6 (4.6,14.5; 0.002)		37.1 (24.6,49.5; <0.001)	
To prevent discrimination based on race or ethnicity				
Never justified	128	49.3 (42.0,56.6)	23	27.7 (16.5,38.9)
Sometimes justified	73	28.1 (21.8,34.4)	18	25.4 (13.4,37.3)
Usually or always justified	45	22.1 (15.7,28.5)	34	43.8 (31.1,56.4)
Adjusted prevalence difference* (95% CI; q-value)	12.3 (6.1,18.4; <0.001)		32.6 (19.7,45.5; <0.001)	
To preserve an American way of life based on Western European traditions				
Never justified	100	42.7 (35.4,49.9)	21	28.2 (16.6,39.8)
Sometimes justified	95	34.2 (27.6,40.8)	23	26.8 (15.5,38.1)
Usually or always justified	49	21.3 (15.1,27.5)	32	42.7 (30.1,55.4)
Adjusted prevalence difference* (95% CI; q-value)	18.7 (12.6,24.9; <0.001)		40.6 (28.1,53.1; <0.001)	
To preserve the American way of life I believe in				
Never justified	58	26.5 (19.9,33.1)	9	11.9 (3.7,20.1)
Sometimes justified	108	42.5 (35.3,49.6)	25	35.8 (23.3,48.4)
Usually or always justified	81	31.1 (24.4,37.8)	42	50.1 (37.2,62.9)
Adjusted prevalence difference* (95% CI; q-value)	23.3 (16.7,29.9; <0.001)		43.9 (31.0,56.8; <0.001)	
To oppose Americans who do not share my beliefs				
Never justified	172	64.4 (57.2,71.6)	29	35.5 (23.3,47.7)
Sometimes justified	53	22.8 (16.6,28.9)	15	19.6 (9.5,29.8)
Usually or always justified	22	12.9 (7.2,18.5)	32	42.6 (29.9,55.3)
Adjusted prevalence difference* (95% CI; q-value)	9.9 (4.7,15.2; 0.003)		39.3 (26.8,51.8; <0.001)	
To oppose the government when it does not share my beliefs				
Never justified	134	53.7 (46.4,61.0)	18	24.8 (13.7,36.0)
Sometimes justified	82	31.6 (24.9,38.4)	23	25.1 (14.2,36.0)
Usually or always justified	29	13.8 (8.6,19.1)	34	46.3 (33.5,59.2)
Adjusted prevalence difference* (95% CI; q-value)	11.6 (6.7,16.6; <0.001)		44.7 (32.2,57.2; <0.001)	
To oppose the government when it tries to take private land for public purpose				
Never justified	75	32.3 (25.4,39.2)	13	17.7 (7.8,27.5)
Sometimes justified	104	36.9 (30.1,43.7)	23	26.9 (16.0,37.8)
Usually or always justified	67	30.3 (23.4,37.2)	40	53.2 (40.5,66.0)
Adjusted prevalence difference* (95% CI; q-value)	22.9 (16.0,29.8; <0.001)		45.4 (32.5,58.2; <0.001)	

* Prevalence differences are adjusted for age, race and ethnicity, gender, education, income, Census division, and rurality and are for the usually or always justified comparison. They are expressed in absolute percentage points. Q-values represent the probability that the given difference would be a false discovery; they represent the expected proportion of “false positives” that would be seen among the collection of all differences whose q-values were at or below the given q-value

For the 17 objectives considered individually, strong approvers were much more likely than non-approvers to see violence as usually or always justified; aPDs exceeded 30% points for 14 of 17 objectives (Table 1; Supplement, Additional File 1, Table S4). Results for individual political objectives varied with the exposures and objectives. For example, violence “to return Donald Trump to the presidency this year” (i.e., in 2022) was considered usually or always justified by 44.7% (95% CI 32.0%, 57.4%) of strong approvers and 1.6% (95% CI 1.1%, 2.1%) of non-approvers (aPD 42.3pp, 95% CI 29.8pp, 54.8pp; q < 0.001).

Strong approvers were substantially more likely than non-approvers to be very or completely willing to damage property (aPD 26.5pp, 95% CI 15.7pp, 37.3pp; q < 0.001), threaten a person (aPD 18.6pp, 95% CI 9.1pp, 28.0pp, q = 0.002), injure a person (aPD 26.9pp, 95% CI 16.0pp, 37.7pp; q < 0.001), or kill a person (aPD 28.3pp, 95% CI 17.6pp, 39.0pp; q < 0.001) (Table 2). They were also more frequently very or completely willing to use violence against specified target populations because of those populations’ occupational or social characteristics (Supplement, Additional File 1, Table S5); for 8 of 9 target populations, aPDs exceeded 20% points.

**Table 2:** Approval of specified organizations and movements and willingness to engage in political violence, by type

In a situation where you think force or violence is justified to advance an important political objective… How willing would *you personally* be to use force or violence in each of these ways?	Approval of Organizations and Movements				
	Non-Approval			Weak Approval	
	Unweighted *n*		Weighted % (95% CI)	Unweighted *n*	Weighted % (95% CI)
To damage property					
Not asked	1060		28.3 (26.7,29.9)	82	10.0 (7.6,12.4)
Not willing	2334		61.5 (59.8,63.3)	598	76.8 (73.4,80.2)
Somewhat willing	267		8.0 (7.0,9.0)	69	9.2 (6.9,11.5)
Very or completely willing	56		1.9 (1.4,2.5)	26	3.6 (2.1,5.1)
Adjusted prevalence difference* (95% CI; q-value)	Referent			2.1 (0.5,3.7; 0.05)	
To threaten or intimidate a person					
Not asked	1060		28.3 (26.7,29.9)	82	10.0 (7.6,12.4)
Not willing	2467		65.4 (63.7,67.1)	606	77.7 (74.4,81.0)
Somewhat willing	165		5.3 (4.4,6.2)	60	7.8 (5.8,9.8)
Very or completely willing	25		0.8 (0.4,1.1)	26	4.0 (2.4,5.7)
Adjusted prevalence difference* (95% CI; q-value)	Referent			3.0 (1.4,4.7; 0.004)	
To injure a person					
Not asked	1060		28.3 (26.7,29.9)	82	10.0 (7.6,12.4)
Not willing	2495		66.3 (64.6,68.0)	616	78.8 (75.5,82.0)
Somewhat willing	130		4.2 (3.4,5.0)	59	8.3 (6.1,10.5)
Very or completely willing	25		0.8 (0.5,1.2)	17	2.2 (1.1,3.3)
Adjusted prevalence difference* (95% CI; q-value)	Referent			1.5 (0.2,2.7; 0.15)	
To kill a person					
Not asked	1060		28.3 (26.7,29.9)	82	10.0 (7.6,12.4)
Not willing	2561		68.3 (66.6,70.0)	637	81.4 (78.3,84.5)
Somewhat willing	64		2.2 (1.6,2.8)	33	5.0 (3.2,6.8)
Very or completely willing	29		0.9 (0.5,1.2)	22	3.0 (1.6,4.4)
Adjusted prevalence difference* (95% CI; q-value)	Referent			2.0 (0.6,3.4; 0.07)	

**In a situation where you think force or violence is justified to advance an important political objective… How willing would** **you personally** **be to use force or violence in each of these ways?**		**Approval of Organizations and Movements**			
		**Moderate Approval**		**Strong Approval**	
		**Unweighted n**	**Weighted % (95% CI)**	**Unweighted n**	**Weighted % (95% CI)**
To damage property					
Not asked		18	8.2 (4.0,12.3)	4	5.4 (−0.2,11.0)
Not willing		162	61.0 (53.8,68.3)	33	45.0 (32.2,57.8)
Somewhat willing		37	14.8 (9.7,19.9)	14	19.8 (9.0,30.6)
Very or completely willing		27	15.1 (9.2,21.0)	26	29.8 (18.5,41.1)
Adjusted prevalence difference* (95% CI; q-value)		12.5 (7.0,18.0; <0.001)		26.5 (15.7,37.3; <0.001)	
To threaten or intimidate a person					
Not asked		18	8.2 (4.0,12.3)	4	5.4 (−0.2,11.0)
Not willing		156	58.1 (50.8,65.4)	32	38.3 (26.0,50.6)
Somewhat willing		47	19.1 (13.5,24.7)	21	33.9 (21.0,46.7)
Very or completely willing		25	14.1 (8.3,20.0)	19	20.2 (11.0,29.5)
Adjusted prevalence difference* (95% CI; q-value)		12.6 (7.2,18.1; <0.001)		18.6 (9.1,28.0; 0.002)	
To injure a person					
Not asked		18	8.2 (4.0,12.3)	4	5.4 (−0.2,11.0)
Not willing		160	59.8 (52.6,67.1)	34	41.7 (29.2,54.2)
Somewhat willing		42	16.6 (11.4,21.9)	16	24.0 (11.9,36.0)
Very or completely willing		26	14.9 (9.0,20.7)	23	28.9 (17.8,40.1)
Adjusted prevalence difference* (95% CI; q-value)		13.4 (7.8,19.0; <0.001)		26.9 (16.0,37.7; <0.001)	
To kill a person					
Not asked		18	8.2 (4.0,12.3)	4	5.4 (−0.2,11.0)
Not willing		171	63.8 (56.5,71.1)	36	48.9 (36.0,61.7)
Somewhat willing		31	12.6 (7.8,17.5)	10	14.4 (4.7,24.2)
Very or completely willing		25	13.4 (7.8,19.0)	26	29.1 (18.4,39.8)
Adjusted prevalence difference* (95% CI; q-value)		12.3 (6.8,17.7; <0.001)		28.3 (17.6,39.0; <0.001)	

* Prevalence differences are adjusted for age, race and ethnicity, gender, education, income, Census division, and rurality and are for the usually or always justified comparison. They are expressed in absolute percentage points. Q-values represent the probability that the given difference would be a false discovery; they represent the expected proportion of “false positives” that would be seen among the collection of all differences whose q-values were at or below the given q-value

Strong approvers more often thought it very or extremely likely that, in a future situation where they considered political violence to be justified, they would be armed (aPD 31.3pp, 95% CI 19.2pp, 43.3pp; q < 0.001), would carry a gun openly (aPD 26.2pp, 95% CI 14.8pp, 37.6pp; q < 0.001), would threaten someone with a gun (aPD 23.0pp, 95% CI 12.4pp, 33.7pp; q < 0.001), and would shoot someone (aPD 16.2pp, 95% CI 6.7pp, 27.7pp; q = 0.03) (Table 3). Nearly 1 in 5 strong approvers (18.1%, 95% CI 8.5%, 27.7%) expected to shoot someone.

**Table 3:** Approval of specified organizations and movements and future firearm possession and use in political violence

Thinking now about the future and all the changes it might bring, how likely is it that you will use a gun in any of the following ways in the next few years—in a situation where you think force or violence is justified to advance an important political objective?	Approval of Organizations and Movements					
	Non-Approval		Weak Approval			
	Unweighted *n*	Weighted % (95% CI)	Unweighted *n*	Weighted % (95% CI)		
I will be armed with a gun.						
Not likely	3365	89.1 (88.0,90.3)	569	71.5 (67.9,75.2)		
Sometimes likely	233	6.8 (5.8,7.7)	105	13.5 (10.9,16.1)		
Very or Extremely likely	109	3.5 (2.8,4.2)	101	14.7 (11.7,17.7)		
Adjusted prevalence difference* (95% CI; q-value)	Referent		10.5 (7.4,13.5; <0.001)			
I will carry a gun openly, so that people know I am armed.						
Not likely	3567	95.2 (94.4,96.1)	659	83.7 (80.7,86.7)		
Sometimes likely	94	2.6 (2.0,3.1)	63	8.2 (6.0,10.3)		
Very or Extremely likely	45	1.6 (1.0,2.1)	51	7.7 (5.4,10.1)		
Adjusted prevalence difference* (95% CI; q-value)	Referent		5.2 (2.9,7.6; <0.001)			
I will threaten someone with a gun.						
Not likely	3677	98.4 (97.9,98.9)	752	96.2 (94.6,97.8)		
Sometimes likely	15	0.5 (0.2,0.7)	17	2.3 (1.1,3.4)		
Very or Extremely likely	8	0.3 (0.0,0.6)	7	1.3 (0.2,2.4)		
Adjusted prevalence difference* (95% CI; q-value)	Referent		0.9 (−0.3,2.0; 0.50)			
I will shoot someone with a gun.						
Not likely	3652	97.6 (97.0,98.3)	735	94.1 (92.2,96.0)		
Sometimes likely	38	1.2 (0.8,1.7)	28	3.8 (2.3,5.3)		
Very or Extremely likely	17	0.6 (0.3,0.9)	12	1.8 (0.7,3.0)		
Adjusted prevalence difference* (95% CI; q-value)	Referent		0.9 (−0.4,2.1; 0.48)			

**Thinking now about the future and all the changes it might bring**,** how likely is it that you will use a gun in any of the following ways in the next few years—in a situation where you think force or violence is justified to advance an important political objective?**	**Approval of Organizations and Movements**					
	**Moderate Approval**		**Strong Approval**			
	**Unweighted n**	**Weighted % (95% CI)**	**Unweighted n**		**Weighted % (95% CI)**	
I will be armed with a gun.						
Not likely	136	53.9 (46.7,61.1)	28		36.2 (23.9,48.6)	
Sometimes likely	37	15.4 (10.1,20.6)	19		25.0 (13.7,36.2)	
Very or Extremely likely	69	29.4 (22.8,35.9)	29		36.6 (24.3,48.8)	
Adjusted prevalence difference* (95% CI; q-value)	24.1 (17.4,30.8; <0.001)		31.3 (19.2,43.3; <0.001)			
I will carry a gun openly, so that people know I am armed.						
Not likely	170	65.9 (58.9,72.9)	36		46.7 (33.8,59.5)	
Sometimes likely	36	16.2 (10.7,21.7)	15		20.9 (10.1,31.8)	
Very or Extremely likely	37	16.8 (11.2,22.5)	25		30.2 (18.9,41.5)	
Adjusted prevalence difference* (95% CI; q-value)	12.8 (7.3,18.4; <0.001)		26.2 (14.8,37.6; <0.001)			
I will threaten someone with a gun.						
Not likely	219	87.0 (82.0,92.0)	46		57.1 (44.4,69.8)	
Sometimes likely	14	7.7 (3.5,11.9)	12		16.8 (6.7,26.9)	
Very or Extremely likely	9	4.0 (1.2,6.8)	18		23.9 (13.2,34.5)	
Adjusted prevalence difference* (95% CI; q-value)	2.8 (−0.1,5.7; 0.39)		23.0 (12.4,33.7; <0.001)			
I will shoot someone with a gun.						
Not likely	207	82.5 (76.9,88.0)	45		55.7 (42.9,68.5)	
Sometimes likely	23	9.5 (5.5,13.5)	17		23.9 (12.7,35.2)	
Very or Extremely likely	12	6.0 (2.3,9.8)	14		18.1 (8.5,27.7)	
Adjusted prevalence difference* (95% CI; q-value)	4.4 (0.9,7.8; 0.10)		16.2 (6.7,25.7; 0.03)			

* Prevalence differences are adjusted for age, race and ethnicity, gender, education, income, Census division, and rurality and are for the usually or always justified comparison. They are expressed in absolute percentage points. Q-values represent the probability that the given difference would be a false discovery; they represent the expected proportion of “false positives” that would be seen among the collection of all differences whose q-values were at or below the given q-value

### Democracy and civil war

Strong approvers and non-approvers differed substantially on beliefs regarding democracy and elections (Table 4). Strong approvers more frequently agreed strongly or very strongly with the statements that “having a strong leader for America is more important than having a democracy” (aPD 40.0pp, 95% CI 27.5pp, 52.6pp; q < 0.001) and that “armed citizens should patrol polling places at election time” (aPD 45.6pp, 95% CI 32.9pp, 58.3pp; q < 0.001). Most strong approvers (54.6%, 95% CI 41.7%, 67.5%) agreed strongly or very strongly that “in the next few years, there will be civil war in the United States” (aPD 38.7pp, 95% CI 25.3pp, 52.1pp; q < 0.001).

**Table 4:** Approval of specified organizations and movements and beliefs concerning democracy in the US

Statement			Approval of Organizations and Movements							
			Non-Approval				Weak Approval			
			Unweighted *n*		Weighted % (95% CI)		Unweighted *n*		Weighted % (95% CI)	
Do you believe that things in this country today are…										
Generally headed in the wrong direction			2874		77.1 (75.6,78.6)		657		84.1 (81.2,87.1)	
Generally headed in the right direction			830		22.4 (20.9,24.0)		117		15.5 (12.6,18.5)	
Adjusted prevalence difference* (95% CI; q-value)			Referent				7.9 (4.6,11.3; <0.001)			
When thinking about democracy in the United States these days, do you believe…?										
There is a serious threat to our democracy.			2913		76.0 (74.4,77.6)		538		66.8 (63.1,70.6)	
There may be a threat to our democracy, but it is not serious.			633		18.4 (17.0,19.9)		178		24.2 (20.8,27.7)	
There is no threat to our democracy.			164		5.1 (4.3,6.0)		60		8.6 (6.3,10.9)	
Adjusted prevalence difference* (95% CI; q-value)			Referent				−9.4 (−13.5,−5.4; <0.001)			
How important do you think it is for the United States to remain a democracy?										
Not important			28		0.9 (0.5,1.3)		18		2.5 (1.3,3.6)	
Somewhat important			104		3.7 (2.9,4.5)		44		7.3 (5.1,9.6)	
Very or extremely important			3583		95.1 (94.2,96.0)		715		90.0 (87.5,92.5)	
Adjusted prevalence difference* (95% CI; q-value)			Referent				1.3 (−0.0,2.6; 0.22)			
Democracy is the best form of government.										
Do not agree			104		3.3 (2.7,4.0)		46		6.4 (4.5,8.4)	
Somewhat agree			590		18.2 (16.7,19.7)		130		18.5 (15.4,21.7)	
Strongly or very strongly agree			3020		78.1 (76.5,79.7)		600		74.8 (71.3,78.3)	
Adjusted prevalence difference* (95% CI; q-value)			Referent				2.6 (0.3,4.8; 0.12)			
These days, American democracy only serves the interests of the wealthy and powerful										
Do not agree			1067		25.3 (23.8,26.8)		241		27.9 (24.5,31.3)	
Somewhat agree			1362		36.7 (35.0,38.4)		253		32.9 (29.2,36.6)	
Strongly or very strongly agree			1288		37.8 (36.0,39.6)		283		39.1 (35.2,43.0)	
Adjusted prevalence difference* (95% CI; q-value)			Referent				0.4 (−3.9,4.7; 0.87)			
Having a strong leader for America is more important than having a democracy										
Do not agree			2798		72.8 (71.1,74.4)		397		49.0 (45.0,52.9)	
Somewhat agree			523		15.3 (14.0,16.7)		173		23.2 (19.8,26.5)	
Strongly or very strongly agree			391		11.5 (10.3,12.7)		207		27.7 (24.1,31.3)	
Adjusted prevalence difference* (95% CI; q-value)			Referent				13.5 (9.8,17.3; <0.001)			
The 2020 election was stolen from Donald Trump, and Joe Biden is an illegitimate president.										
Do not agree			3415		91.8 (90.8,92.8)		420		56.5 (52.6,60.3)	
Somewhat agree			159		4.1 (3.4,4.7)		141		17.1 (14.3,19.8)	
Strongly or very strongly agree			142		3.9 (3.2,4.6)		212		26.0 (22.6,29.4)	
Adjusted prevalence difference* (95% CI; q-value)			Referent				21.1 (17.7,24.5; <0.001)			
Armed citizens should patrol polling places at election time.										
Do not agree			3575		95.5 (94.8,96.3)		607		76.8 (73.4,80.3)	
Somewhat agree			92		2.6 (2.0,3.2)		97		13.6 (10.9,16.4)	
Strongly or very strongly agree			48		1.6 (1.1,2.1)		70		9.2 (6.8,11.6)	
Adjusted prevalence difference* (95% CI; q-value)			Referent				6.7 (4.3,9.1; <0.001)			
In the next few years, there will be civil war in the United States.										
Do not agree			2173		57.3 (55.5,59.1)		321		39.8 (36.0,43.6)	
Somewhat agree			1233		33.1 (31.4,34.8)		313		39.6 (35.8,43.5)	
Strongly or very strongly agree			297		9.1 (8.0,10.2)		142		20.4 (17.0,23.8)	
Adjusted prevalence difference* (95% CI; q-value)			Referent				9.4 (5.9,12.9; <0.001)			

Statement			Approval of Organizations and Movements							
			**Moderate Approval **				**Strong Approval **			
			Unweighted *n*		Weighted % (95% CI)		Unweighted *n*		Weighted % (95% CI)	
Do you believe that things in this country today are…										
Generally headed in the wrong direction			210		80.3 (73.8,86.8)		50		56.9 (43.8,69.9)	
Generally headed in the right direction			36		19.0 (12.6,25.4)		26		40.5 (27.4,53.5)	
Adjusted prevalence difference* (95% CI; q-value)			5.5 (−0.8,11.8; 0.19)				−14.4 (−27.0,−1.8; 0.07)			
When thinking about democracy in the United States these days, do you believe…?										
There is a serious threat to our democracy.			176		63.7 (56.3,71.0)		43		50.8 (37.9,63.6)	
There may be a threat to our democracy, but it is not serious.			45		23.0 (16.3,29.6)		18		25.1 (13.6,36.6)	
There is no threat to our democracy.			25		12.9 (7.4,18.3)		16		24.1 (12.9,35.3)	
Adjusted prevalence difference* (95% CI; q-value)			−8.1 (−15.1,−1.1; 0.06)				−14.5 (−26.4,−2.6; 0.05)			
How important do you think it is for the United States to remain a democracy?										
Not important			13		5.9 (2.6,9.2)		4		6.9 (0.1,13.8)	
Somewhat important			34		17.8 (11.6,24.0)		14		21.4 (10.3,32.5)	
Very or extremely important			200		76.2 (69.6,82.9)		58		70.9 (58.8,83.0)	
Adjusted prevalence difference* (95% CI; q-value)			3.6 (0.4,6.8; 0.19)				3.7 (−2.9,10.4; 0.48)			
Democracy is the best form of government.										
Do not agree			27		11.9 (7.0,16.7)		12		13.4 (5.1,21.6)	
Somewhat agree			78		34.2 (27.2,41.1)		15		26.2 (14.1,38.4)	
Strongly or very strongly agree			140		52.8 (45.5,60.0)		50		60.4 (47.6,73.2)	
Adjusted prevalence difference* (95% CI; q-value)			5.7 (0.9,10.5; 0.12)				4.9 (−2.3,12.2; 0.39)			
These days, American democracy only serves the interests of the wealthy and powerful.										
Do not agree			53		18.9 (13.5,24.3)		14		17.0 (7.6,26.5)	
Somewhat agree			82		34.4 (27.5,41.3)		23		36.0 (23.2,48.9)	
Strongly or very strongly agree			110		45.5 (38.3,52.8)		40		46.9 (34.2,59.6)	
Adjusted prevalence difference* (95% CI; q-value)			2.0 (−5.7,9.6; 0.76)				−2.6 (−16.8,11.7; 0.76)			
Having a strong leader for America is more important than having a democracy.										
Do not agree			102		33.9 (27.4,40.4)		19		22.8 (12.6,33.0)	
Somewhat agree			64		27.3 (20.9,33.7)		12		19.4 (8.7,30.1)	
Strongly or very strongly agree			79		37.6 (30.2,45.0)		45		56.6 (43.9,69.3)	
Adjusted prevalence difference* (95% CI; q-value)			22.6 (15.3,29.9; <0.001)				40.0 (27.5,52.6; <0.001)			
The 2020 election was stolen from Donald Trump, and Joe Biden is an illegitimate president.										
Do not agree			46		18.7 (13.1,24.3)		8		10.6 (3.0,18.2)	
Somewhat agree			67		29.3 (22.4,36.1)		20		35.3 (22.5,48.2)	
Strongly or very strongly agree			134		52.0 (44.7,59.3)		48		52.4 (39.5,65.3)	
Adjusted prevalence difference* (95% CI; q-value)			47.7 (40.9,54.5; <0.001)				50.9 (38.4,63.4; <0.001)			
Armed citizens should patrol polling places at election time.										
Do not agree			111		39.1 (32.2,46.1)		11		15.2 (5.6,24.8)	
Somewhat agree			75		32.2 (25.4,39.1)		24		34.6 (22.2,47.1)	
Strongly or very strongly agree			60		28.3 (21.5,35.1)		41		49.0 (36.2,61.8)	
Adjusted prevalence difference* (95% CI; q-value)			24.9 (18.3,31.5; <0.001)				45.6 (32.9,58.3; <0.001)			
In the next few years, there will be civil war in the United States.										
Do not agree			55		22.1 (15.8,28.4)		8		12.4 (3.5,21.3)	
Somewhat agree			109		39.9 (33.0,46.9)		23		33.0 (20.6,45.4)	
Strongly or very strongly agree			81		36.8 (29.6,43.9)		46		54.6 (41.7,67.5)	
Adjusted prevalence difference* (95% CI; q-value)			23.6 (16.4,30.9; <0.001)				38.7 (25.3,52.1; <0.001)			

* Prevalence differences are adjusted for age, race and ethnicity, gender, education, income, Census division, and rurality and are for the usually or always justified comparison. They are expressed in absolute percentage points. Q-values represent the probability that the given difference would be a false discovery; they represent the expected proportion of “false positives” that would be seen among the collection of all differences whose q-values were at or below the given q-value

### Non-political violence

Majorities of respondents across all levels of approval saw violence as usually or always justified in self-defense or to prevent someone from injuring another person or themselves; there were no significant differences between strong approvers and non-approvers (Additional file 1, Supplement, Table S6). Strong approvers were much more likely than non-approvers to endorse violence “to prevent harm or damage to property” (aPD 28.6pp, 95% CI 16.4pp, 40.8pp; q < 0.001), “to win an argument” (aPD 30.0pp, 95% CI 18.1pp, 41.8pp; q < 0.001), “in response to an insult” (aPD 35.6pp, 95% CI 22.7pp, 48.6pp; q < 0.001), and “to get respect” (aPD 46.0pp, 95% CI 33.4pp, 58.6pp; q < 0.001).

## Approval of individual organizations and movements

Prevalences of strong or very strong approval for the 8 individual organizations and movements did not exceed 5% (Additional File 1, Supplement, Table S1). For all but the Christian nationalist movement, a large majority (range, 71.9% to 88.8%) of respondents reporting strong or very strong approval for any 1 organization or movement also did so for at least 1 of the others.

### Political violence

At least 70% of strong or very strong approvers of each of these organizations or movements—90.5% for the boogaloo movement—considered violence usually or always justified to advance at least 1 of 17 objectives, and at least 20% (not infrequently > 40%) considered violence usually or always justified for each objective considered individually (Additional file 1, Supplement, Tables S7, S8). Occasional exceptions were limited to the Oath Keepers and the Christian nationalist movement. Most prevalences for non-approvers were below 10%.

Among strong or very strong approvers, very or complete willingness to kill a person ranged from 10.9% (95% CI 7.1%, 14.6%) for the Christian nationalist movement to 41.5% (95% CI 27.6%, 55.3%) for the boogaloo movement (Additional file 1, Supplement, Table S9). Very or complete willingness to use violence against members of 9 target populations varied and was highest (prevalences generally > 30%) among strong or very strong approvers of the white supremacy and boogaloo movements (Additional file 1, Supplement, Table S10).

Expectations of firearm involvement in political violence were common. From 6.4% (95% CI 2.3%, 10.6%) of strong or very strong approvers of the Proud Boys to 25.1% (95% CI 12.6%, 37.6%) of strong or very strong approvers of the boogaloo movement thought it very or extremely likely that they would shoot someone in a future situation where they thought political violence was justified (Additional file 1, Supplement, Table S11).

For all measures of willingness to engage in political violence and of firearm use in political violence, prevalences for non-approvers were below 3% (Additional file 1, Supplement, Tables S9-S11).

### Democracy and civil war

At least 40% of strong or very strong approvers of these organizations or movements—more than 60% for the white supremacy movement—agreed strongly or very strongly that “having a strong leader for America is more important than having a democracy” (Additional file 1, Supplement, Table S12). Except in the case of the Christian Nationalist movement, at least 40% agreed that “armed citizens should patrol polling places at election time” and that “in the next few years, there will be civil war in the United States.” Prevalences for non-approvers were below 20% for the strong leader statement, below 5% for the polling places item, and below 15% for expectation of civil war.

### Non-political violence

Support for violence “to win an argument,” “in response to an insult,” or “to get respect” was much more common among strong or very strong approvers than among non-approvers (Additional file 1, Supplement, Table S13). Between 20% and 50% of strong or very strong approvers of 7 of these organizations or movements, and 15%−20% in the case of the Christian nationalist movement, considered violence usually or always justified for those purposes. Prevalences for non-approvers were below 4%.

## Discussion

In this large, nationally representative survey in the United States, strong approval of specified extreme right-wing organizations and social movements was associated with high levels of support for and personal willingness to engage in political violence, including lethal violence and the use of firearms. Differences between approvers and non-approvers were often strikingly large.

These associations have not previously been quantified, to our knowledge, but they are not unexpected. They validate current expert assessments that these beliefs, organizations, and movements pose not just a general social hazard but a specific threat to democracy in the US [7–13]. For example, in endorsing violence “to return Donald Trump to the presidency this year” (in 2022), 44.7% of strong approvers were endorsing the violent overthrow of a legitimately elected federal government.

Support for political violence may reflect a general predisposition to the use of violence among these respondents, who were also more likely than non-approvers (and the general population [4]) to endorse violence to win an argument, in response to an insult, and to get respect.

Strong approvers accounted for just 1.4% of the study population; they were greatly outnumbered by non-approvers (and weak approvers) whose support for violence was often dramatically lower. At the same time, comparison of the estimated proportions of the adult population actively involved in the white supremacy movement [14] or Proud Boys [18] with our estimates of the prevalence of strong approval for them (Additional file 1, Supplement, Table S1) indicates that approval is far more common than involvement: by a factor of roughly 30- to 40-fold in the former case and 200- to 1000-fold in the latter.

Moreover, and unlike other respondents in our surveys who saw political violence as justified [4–6, 43–45], strong approvers who justified violence were frequently willing to engage in that violence themselves. This is consistent with the research linking “right-wing ideological values” to violence in the general population [41, 42].

In 2025, experts have suggested that the risk of violence in this population has been increased still further by the pardons given to the January 6 offenders [58, 59]; impunity is emboldening [19]. There have been calls—and militia movement volunteers—for a private “army” to implement mass deportations [60, 61]. The possibility that persons resembling the strong approvers in this study could be deployed as members of such an army or as federal law enforcement agents— including to implement the administration’s newly announced Project Homecoming—is cause for great concern [33–38].

Our findings support focused efforts to identify, dissuade, deter, and incapacitate individuals and organizations that pose threats of political violence, along with efforts to celebrate and reinforce the majority view that political violence is unacceptable [62]. The Center for Prevention Programs and Partnerships (CP3) at the Department of Homeland Security is based on a public health model [63, 64]. Others have raised concerns about its approach [65, 66] for several years, however, and in 2025, CP3 was placed under the direction of a 22-year-old with no national security experience [67]. Recent research has identified potential interventions [7, 68–73]. but there is limited evidence of their long-term efficacy or population-level effectiveness [73, 74]. The finding from another survey of this cohort [75] that many who expect to be combatants in a civil war are open to change in response to input from their family and others reinforces the suggestion that suasion by family members, friends, and others is efficacious [76].

### Limitations

These data were collected in 2022, when political violence was considered most likely to arise from the political right [7, 8, 11–14]. Subsequent events raise the possibility of a threat from the left, but there are few left-wing violent extremist organizations and movements in the US. The findings are cross-sectional and subject to sampling error and bias due to nonresponse, social desirability, and strategic responding. The cross-sectional data limit us to reporting associations; we do not draw causal inferences. Some response counts are small, producing weighted prevalences below 5%. Support for violence to advance specific objectives is in part a function of the objectives presented; a different list might have produced different results. We did not ask respondents if they were members of the specified organizations and movements, choosing “approval” as a broader measure of affiliation. Many respondents could not be classified on our exposure measure; this has been seen elsewhere [39]. Mass shootings in Buffalo, NY (a race-related hate crime motivated by great-replacement thinking [77]) and Uvalde, TX while the survey was in the field may have affected our findings.

We will address several of these limitations in future research. The survey has been conducted annually (Wave 4 was in the field in May-June 2025), with additions to the list of political objectives for which respondents might see violence as justified and with support for these 8 organizations and movements remeasured in 2025. The survey is longitudinal; it provides linked data on individuals over time, and we plan to assess trends in support for violence among approvers of right- (and left-) wing extremism in response to events such as the 2024 US presidential election.

## Conclusions

Political violence is strongly associated with approval of extreme right-wing organizations and social movements. High levels of support for and willingness to engage in political violence are concentrated in a small percentage of the population. Such findings can facilitate the orientation and development of efforts to prevent political violence.

## Supplementary Information

Supplementary Material 1: Approval of Extreme Right-Wing Organizations and Social Movements and Support for Political Violence in the United States: Findings from a Nationally Representative Survey

## Data Availability

The datasets generated and/or analyzed during the current study will be made available to qualified researchers subject to the terms of a data use agreement.

## Abbreviations

CI: Confidence interval
SD: Standard deviation
aPD: Adjusted prevalence difference
pp: Percentage point
