# Supplementary material for: Approval of extreme right-wing organizations and social movements and support for political violence in the United States: findings from a nationally representative survey
Source: Inj Epidemiol. 2025 Dec 8;12:82. doi: 10.1186/s40621-025-00631-8 (PMC12683887; doi:10.1186/s40621-025-00631-8)
Supplement: Supplementary file 1 — Supplementary Material 1: Approval of Extreme Right-Wing Organizations and Social Movements and Support for Political Violence in the United States: Findings from a Nationally Representative Survey [file 40621_2025_631_MOESM1_ESM.pdf]

Supplement

Approval of Extreme Right-Wing Organizations and Social Movements

and Support for Political Violence in the United States:

Findings from a Nationally Representative Survey

Garen J. Wintemute, MD, MPH; Bradley Velasquez, MS; Yueju Li, MA; Elizabeth A. Tomsich, PhD; Paul M. Reeping, PhD, MS; Daniel Tancredi, PhD; Sonia L. Robinson, PhD, MPH

This supplement has been provided by the authors to give readers additional information about the work.

Page	Item
2	Questions that supplied data for this study
6	References for the question list
7	Additional methods text
8	Table S1. Prevalence of approval of individual organizations and movements
10	Table S2. Personal characteristics of respondents
13	Table S3. Comparison of respondents with non-respondents (weights were not available for nonrespondents)
16	Table S4. Approval of specified organizations and movements and justification for violence to advance 8 additional specific political objectives
18	Table S5. Approval of specified organizations and movements and personal willingness to engage in political violence, by target of violence
20	Table S6. Approval of specified organizations and movements and justification for violence in non-political situations
22	Table S7. Approval of individual organizations and movements and justification for political violence, in general and to advance 9 specific political objectives
31	Table S8. Approval of individual organizations and movements and justification for violence to advance 8 additional specific political objectives
40	Table S9. Approval of individual organizations and movements and personal willingness to engage in political violence, by type of violence
48	Table S10. Approval of individual organizations and movements and personal willingness to engage in political violence, by target of violence
57	Table S11. Approval of individual organizations and movements and future likelihood of firearm possession and use in a situation where political violence is perceived as justified
65	Table S12. Approval of individual organizations and movements and beliefs concerning democracy in the US
74	Table S13. Approval of individual organizations and movements and justification for violence in non-political situations

QUESTIONS THAT SUPPLIED DATA FOR THIS STUDY

In the list below, questions or items that were repeated or adapted from prior surveys contain citations to those surveys. Items used for principal exposures were not presented en bloc. An (R) indicates that an item was reverse coded.

Approval of specified organizations and movements

Q: How much do you approve or disapprove of these named groups and organizations?

Proud Boys
Oath Keepers
Three Percenters
QAnon

1. Do not approve
2. Somewhat approve
3. Strongly approve
4. Very strongly approve
5. I don’t know enough about this group or organization to rate it
6. I have never heard of this group or organization

Q: How much do you approve or disapprove of these political or social movements?

The militia movement
The white supremacy movement
The Christian nationalist movement
The boogaloo movement

1. Do not approve
2. Somewhat approve
3. Strongly approve
4. Very strongly approve
5. I don’t know enough about this group or organization to rate it
6. I have never heard of this group or organization

Democracy and US institutions

Q: Do you believe that things in this country today are…?[4]

Generally headed in the wrong direction
Generally headed in the right direction

Q: When thinking about democracy in the United States these days, do you believe…?[5]

There is a serious threat to our democracy.
There may be a threat to our democracy, but it is not serious.
There is no threat to our democracy.

Q: How important do you think it is for the United States to remain a democracy?[6]

Not important

Somewhat important
Very important
Extremely important

Q: How much do you agree or disagree with the following statements about democracy in the United States?

Democracy is the best form of government.[7]
These days, American democracy only serves the interests of the wealthy and powerful.[1]
Having a strong leader for America is more important than having a democracy.
In the next few years, there will be civil war in the United States.[8]

1. Do not agree
2. Somewhat agree
3. Strongly agree
4. Very strongly agree

Q: People have many different views about American society. How much do you agree or disagree with each of the following?

The 2020 election was stolen from Donald Trump, and Joe Biden is an illegitimate president.
Armed citizens should patrol polling places at election time.

1. Do not agree
2. Somewhat agree
3. Strongly agree
4. Very strongly agree

Violence, including political violence

Q: In general, what do you think about the use of force or violence in the following situations—is it never justified, sometimes justified, usually justified, or always justified? "Force or violence" means physical force strong enough that it could cause pain or injury to a person.

(Not randomized)

In self defense
To prevent someone from injuring or killing another person
To prevent someone from injuring or killing themselves
To prevent harm or damage to property
To win an argument
In response to an insult
To get respect

1. Never justified
2. Sometimes justified
3. Usually justified
4. Always justified

Q: People sometimes talk about using force or violence to achieve political objectives. In general, what do <u>you</u> think about using force or violence to advance an important political objective that you support—is it…?

1. Never justified

2. Sometimes justified
3. Usually justified
4. Always justified

Q: You said that in general, the use of force or violence was [response inserted] to advance an important political objective that you support. Your opinion might depend on the specific objective that was involved. What do you think about the use of force or violence in the following situations—is it never justified, sometimes justified, usually justified, or always justified?

[Items 1a-4b were paired, and each respondent was presented with 1 item from each pair.]

1a. To stop voter fraud
OR
1b. To stop voter intimidation

2a. To stop police violence
OR
2b. To reinforce the police

3a. To stop illegal immigration
OR
3b. To keep our borders open

4a. To stop a protest or demonstration
OR
4b. To support a protest or demonstration

5. To preserve the American way of life I believe in
6. To oppose Americans who do not share my beliefs

1. Never justified
2. Sometimes justified
3. Usually justified
4. Always justified

Q: Again, your view of the use of force or violence to advance an important political objective might depend on the specific objective that was involved. What do you think about the use of force or violence in the following situations—is it never justified, sometimes justified, usually justified, or always justified?

To return Donald Trump to the presidency this year
To stop an election from being stolen
To stop people who do not share my beliefs from voting
To prevent discrimination based on race or ethnicity
To preserve an American way of life based on Western European traditions
To oppose the government when it does not share my beliefs
To oppose the government when it tries to take private land for public purposes

1. Never justified
2. Sometimes justified
3. Usually justified
4. Always justified

(Questions asked of respondents who endorsed—as sometimes, usually, or always justified—1 or more uses of violence to achieve specific political objectives.)

Q: In a situation where you think force or violence is justified to advance an important political objective, how willing would you personally be to use force or violence in each of these ways?

To damage property
To threaten or intimidate a person
To injure a person
To kill a person

1. Not willing
2. Somewhat willing
3. Very willing
4. Completely willing

Q: In a situation where you think force or violence is justified to advance an important political objective, how willing would you personally be to use force or violence against a person because they are…

An elected federal or state government official
An elected local government official
A public health official
A member of the military or National Guard
A police officer
A person who does not share your race or ethnicity
A person who does not share your religion
An election worker, such as a poll worker or vote counter
A person who does not share your political beliefs

1. Not willing
2. Somewhat willing
3. Very willing
4. Completely willing

(Question asked of all respondents.)

Q: Thinking now about the future and all the changes it might bring, how likely is it that you will use a gun in any of the following ways in the next few years—in a situation where you think force or violence is justified to advance an important political objective?

I will be armed with a gun.
I will carry a gun openly, so that people know I am armed.
I will threaten someone with a gun.
I will shoot someone with a gun.

1. Not likely
2. Somewhat likely
3. Very likely
4. Extremely likely

REFERENCES FOR THE QUESTION LIST

1. Survey Center on American Life. January 2021 American Perspectives Survey topline questionnaire. https://www.americansurveycenter.org/wp-content/uploads/2021/03/January-2021-APS-Topline-Questionnaire.pdf.

2. Pew Research Center. Americans see advantages and challenges in country's growing racial and ethnic diversity. 2019 May. https://www.pewresearch.org/social-trends/2019/05/08/americans-see-advantages-and-challenges-in-countrys-growing-racial-and-ethnic-diversity/.

3. Public Religion Research Institute. The persistence of Q-Anon in the post-Trump era: an analysis of who believes the conspiracies. 2022 Feb 24. https://www.prri.org/research/the-persistence-of-qanon-in-the-post-trump-era-an-analysis-of-who-believes-the-conspiracies/.

4. Economist/YouGov Poll June 13 - 15, 2021 - 1500 U.S. Adult Citizens. https://docs.cdn.yougov.com/uagnfc262c/econTabReport.pdf.

5. NPR/PBS NewsHour/Marist National Poll. Trust in elections, threat to democracy, November 2021. 2021 November 1. https://maristpoll.marist.edu/polls/npr-pbs-newshour-marist-national-poll-trust-in-elections-threat-to-democracy-biden-approval-november-2021/.

6. Grinnell College National Poll. 52% of Americans believe democracy facing "major threat." Study #2243. 2021 October 20. https://www.grinnell.edu/news/52-americans-believe-democracy-facing-major-threat.

7. The Economist/YouGov Poll. 2021 June 13-16. https://docs.cdn.yougov.com/uagnfc262c/econTabReport.pdf.

8. Zogby. Will the US have another civil war? 2021 Feb 4. https://zogbyanalytics.com/news/997-the-zogby-poll-will-the-us-have-another-civil-war

9. IFYC – PRRI Survey on Religion & COVID-19 Vaccine Trust. 2021 March. https://www.prri.org/wp-content/uploads/2021/05/Topline-IFYC-PRRI-Survey-on-Religion-and-COVID-19-Vaccine-Trust-v2_final.pdf.

ADDITIONAL METHODS TEXT

Questionnaire design

To minimize inattentive responses to questions regarding political violence, questions on that topic were immediately preceded by a question asking respondents about the justifiability of the use of force or violence in 7 non-political situations. These were presented to all respondents in a fixed order from what the authors considered likely to be seen as justifying violence ("in self-defense") to unlikely ("to get respect"). This was done to create an expected response transition from support to nonsupport that respondents would need to reverse to indicate support for political violence. Questions for which responses might invoke a skip pattern included non-response prompts.

Exposure categorization

For each exposure, summed scores for respondents with missing data for up to half the items were normalized to a range from 0 to 1, with 0 and 1 representing the minimum and maximum theoretically possible scores given the number of items missing. Respondents with missing data for half or more of the items for an exposure were excluded from categorization. Missingness resulted almost entirely from respondents indicating that they did not know enough about an organization or movement to rate it or that they had never heard of a group or organization. Frequencies of these responses for each organization or movement are presented in Table S6. True non-response for these items did not exceed 2% (Table S6).

Model development

We examined the following models for adjusting prevalence differences:

Model 0: unadjusted;

Model 1: adjusted for age (numerical), race and ethnicity (White, Non-Hispanic; Black, Non-Hispanic; Other, Non-Hispanic; Hispanic; 2+ Races, Non-Hispanic), and gender (Male, Female);

Model 2: additionally adjusted for income (Less than \$10,000, \$10,000 to \$24,999, \$25,000 to \$49,999, \$50,000 to \$74,999, \$75,000 to \$99,999, \$100,000 to \$149,999, \$150,000 or more), education (No high school diploma or GED, High school graduate (high school diploma or the equivalent GED), Some college or Associate's degree, Bachelor's degree, Master's degree or higher), and Census division (New England, Mid-Atlantic, East-North Central, West-North Central, South Atlantic, East-South Central, West-South Central, Mountain, Pacific);

Model 3: additionally adjusted for rurality (Urban, Rural; derived from Rural-Urban Commuting Codes matched to census tracts (https://www.ers.usda.gov/data-products/rural-urban-commuting-area-codes/).

Model 3 was selected; findings from Model 3 appear in the 'Adjusted prevalence difference" rows in tables. Q-values for comparisons in the text were also produced using Model 3.

Table S1. Prevalence of approval of individual organizations and movements

Approval	Proud Boys		Oath Keepers		Three Percenters		QAnon	
	Unweighted n	Weighted % (95% CI)	Unweighted n	Weighted % (95% CI)	Unweighted n	Weighted % (95% CI)	Unweighted n	Weighted % (95% CI)
**For named organization/movement**								
Do not approve	4882	53.1 (52.0,54.3)	3447	35.7 (34.6,36.8)	2423	25.9 (24.9,26.9)	4740	51.3 (50.1,52.5)
Somewhat approve	324	4.1 (3.6,4.6)	321	3.8 (3.4,4.3)	197	2.6 (2.2,2.9)	209	2.8 (2.3,3.2)
Strongly approve	83	1.2 (0.9,1.5)	119	1.7 (1.3,2.0)	63	1.1 (0.8,1.3)	64	1.1 (0.8,1.4)
Very strongly approve	78	1.1 (0.8,1.4)	99	1.4 (1.1,1.7)	55	0.8 (0.6,1.1)	54	0.7 (0.5,1.0)
I don't know enough about this group or organization to rate it	1706	19.9 (19.0,20.9)	2064	23.9 (22.9,24.9)	2331	26.5 (25.4,27.5)	1972	23.3 (22.3,24.3)
I have never heard of this group or organization	1444	19.1 (18.1,20.1)	2455	32.0 (30.9,33.2)	3436	41.7 (40.5,42.9)	1465	19.3 (18.3,20.3)
No response	103	1.4 (1.1,1.7)	115	1.5 (1.2,1.8)	115	1.5 (1.2,1.8)	116	1.5 (1.2,1.8)
**For other organizations/movements**								
Strong/very strong approval for named organization/movement only	51	28.1 (20.5,35.7)	74	27.5 (21.2,33.7)	29	19.9 (12.4, 27.4)	36	23.7 (15.5,31.9)
Strong/very strong approval for at least 1 additional organization/movement	110	71.9 (64.3,79.5	144	72.5 (66.3,78.8)	89	80.1 (72.6,87.6)	82	76.3 (68.1,84.5)

Table S1, continued.

Approval	Christian Nationalist Movement		White Supremacy Movement		Militia Movement		Boogaloo Movement	
	Unweighted n	Weighted % (95% CI)	Unweighted n	Weighted % (95% CI)	Unweighted n	Weighted % (95% CI)	Unweighted n	Weighted % (95% CI)
**For named organization/movement**								
Do not approve	3284	35.8 (34.7,37.0)	6833	76.3 (75.3,77.4)	4301	45.4 (44.3,46.6)	2635	28.3 (27.3,29.3)
Somewhat approve	571	6.5 (5.9,7.1)	163	2.3 (1.9,2.7)	394	4.8 (4.3,5.4)	97	1.5 (1.2,1.9)
Strongly approve	196	2.5 (2.1,2.9)	35	0.5 (0.3,0.7)	87	1.4 (1.1,1.7)	26	0.5 (0.3,0.8)
Very strongly approve	160	2.1 (1.8,2.5)	38	0.6 (0.4,0.8)	55	0.8 (0.5,1.0)	31	0.5 (0.3,0.7)
I don't know enough about this group or organization to rate it	2367	27.4 (26.3,28.4)	888	11.2 (10.4,12.0)	2092	25.3 (24.3,26.3)	2093	24.4 (23.4,25.4)
I have never heard of this group or organization	1916	23.9 (22.8,24.9)	537	7.4 (6.7,8.1)	1573	20.7 (19.7,21.7)	3632	43.4 (42.2,44.5)
No response	126	1.7 (1.4,2.1)	126	1.6 (1.3,2.0)	118	1.6 (1.3,1.9)	106	1.4 (1.1,1.7)
**For other organizations/movements**								
Strong/very strong approval for named organization/movement only	239	61.4 (55.3,67.6)	16	13.9 (6.7,23.0)	42	23.2 (15.9,30.4)	9	11.2 (3.2,19.2)
Strong/very strong approval for at least 1 additional organization/movement	117	38.6 (32.4,44.7)	57	86.1 (79.0, 93.3)	100	76.8 (69.6,84.1)	48	88.8 (80.8,96.8)

Table S2. Personal characteristics of respondents

Characteristic	Respondents (n= 8620)	
	Unweighted n	Weighted % (95% CI)
**Age**		
18-24	447	10.5 (9.6, 11.5)
25-34	1024	16.6 (15.6, 17.6)
35-44	1374	18.5 (17.6, 19.5)
45-54	1215	14.5 (13.7, 15.3)
55-64	1833	17.4 (16.6, 18.2)
65-74	1788	14.4 (13.7, 15.1)
75+	939	8.0 (7.4, 8.5)
Non-response	0	0
**Gender**		
Female	4300	50.6 (49.4, 51.7)
Male	4159	47.2 (46.1, 48.4)
Transgender	41	0.6 (0.4, 0.8)
Non-binary	44	0.7 (0.5, 0.9)
Other	20	0.3 (0.1, 0.4)
Non-response	56	0.7 (0.5, 0.9)
**Race and ethnicity**		
White, Non-Hispanic	6046	62.6 (61.4, 63.9)
Black, Non-Hispanic	834	11.9 (11.1, 12.8)
Hispanic, any race	1084	16.9 (15.9, 17.8)
American Indian or Alaska Native, Non-Hispanic	54	1.3 (0.9, 1.6)
Asian American / Pacific Islander, non-Hispanic	313	5.4 (4.8, 6.1)
Some other race, Non-Hispanic	22	0.1 (0.1, 0.2)
2+ Races, Non-Hispanic	267	1.7 (1.5, 2.0)
Non-response	0	0
**Marital status**		
Now married	5246	56.1 (54.9, 57.3)
Widowed	443	4.0 (3.6, 4.4)
Divorced	909	8.7 (8.1, 9.3)
Separated	139	1.7 (1.4, 2.1)
Never married	1883	29.5 (28.3, 30.7)
Non-response	0	0

Table S2, continued.

Characteristic	Respondents (n= 8620)	
	Unweighted n	Weighted % (95% CI)
**Education**		
No high school diploma or GED	542	9.5 (8.7, 10.4)
High school graduate (diploma or GED)	2158	28.3 (27.2, 29.4)
Some college or Associate's degree	2364	27.1 (26.0, 28.1)
Bachelor's degree	1951	19.7 (18.8, 20.6)
Master's degree or higher	1605	15.4 (14.7, 16.2)
Non-response	0	0
**Household Income**		
Less than $10,000	272	3.9 (3.4, 4.4)
$10,000 to $24,999	745	9.0 (8.3, 9.7)
$25,000 to $49,999	1469	17 (16.1, 17.9)
$50,000 to $74,999	1414	16.3 (15.4, 17.2)
$75,000 to $99,999	1214	13.2 (12.4, 14)
$100,000 to $149,999	1500	17.9 (16.9, 18.8)
$150,000 or more	2006	22.8 (21.8, 23.7)
Non-response	0	0
**Employment**		
Working - as a paid employee	4323	54.3 (53.1, 55.4)
Working - self-employed	694	8.0 (7.3, 8.6)
Not working - on temporary layoff from a job	40	0.6 (0.4, 0.8)
Not working - looking for work	312	5.1 (4.5, 5.7)
Not working - retired	2478	20.9 (20.1, 21.8)
Not working - disabled	314	4.2 (3.7, 4.7)
Not working - other	459	7.0 (6.3, 7.7)
Non-response	0	0
**Census division**		
New England	412	4.7 (4.2, 5.2)
Mid-Atlantic	1090	12.5 (11.8, 13.3)
East-North Central	1267	14.3 (13.5, 15.1)
West-North Central	604	6.4 (5.8, 6.9)
South Atlantic	1714	20.5 (19.5, 21.4)
East-South Central	465	5.8 (5.3, 6.4)
West-South Central	904	12.0 (11.1, 12.8)
Mountain	745	7.7 (7.1, 8.2)
Pacific	1419	16.2 (15.3, 17.1)
Non-response	0	0

Table S2, continued.

This table was previously published in Wintemute GJ, Robinson SL, Crawford A, Tancredi D, Schleimer JP, Tomsich EA, Reeping PM, Shev AB, Pear VA. Views of democracy and society and support for political violence in the USA: findings from a nationally representative survey. Injury Epidemiology. 2023;10(1):45. DOI: 10.1186/s40621-023-00456-3.

Table S3. Comparison of respondents with nonrespondents (weights were not available for nonrespondents)

Characteristic	Respondents (n = 8,620)		Non-respondents (n = 6,099)	
	Unweighted n	Unweighted %	Unweighted n	Unweighted %
Age				
18-24	447	5.2	977	16.0
25-34	1024	11.9	1129	18.5
35-44	1374	15.9	1340	22.0
45-54	1215	14.1	1151	18.9
55-64	1833	21.3	811	13.3
65-74	1788	20.7	502	8.2
75+	939	10.9	189	3.1
Non-response	0	0.0	0	0.0
Gender				
Male	4247	49.3	3313	54.3
Female	4373	50.7	2786	45.7
Non-response	0	0.0	0	0.0
Race and ethnicity				
White, non-Hispanic	6047	70.2	3528	57.9
Black, non-Hispanic	836	9.7	836	13.7
Other, non-Hispanic	392	4.5	1183	19.4
Hispanic	1084	12.6	304	5.0
2+ races, non-Hispanic	261	3.0	248	4.1
Non-response	0	0.0	0	0.0
Marital status				
Now married	5246	60.9	3128	51.3
Widowed	443	5.1	168	2.8
Divorced	909	10.5	577	9.5
Separated	139	1.6	141	2.3
Never married	1883	21.8	2085	34.2
Non-response	0	0.0	0	0.0

Table S3, continued.

Characteristic	Respondents (n = 8,620)		Non-respondents (n = 6,099)	
	Unweighted n	Unweighted %	Unweighted n	Unweighted %
Education				
No high school diploma or GED	542	6.3	625	10.3
High school graduate (HS diploma or GED)	2158	25.0	1759	28.8
Some college or Associate degree	2364	27.4	1769	29.0
Bachelor's degree	1951	22.6	1140	18.7
Master's degree or higher	1605	18.6	806	13.2
Non-response	0	0.0	0	0.0
Household income				
< $10,000	272	3.2	312	5.1
$10,000 - $24,999	745	8.6	609	10.0
$25,000 - $49,999	1469	17.0	1158	19.0
$50,000 - $74,999	1414	16.4	1012	16.6
$75,000 - $99,999	1214	14.1	810	13.3
$100,000 - $149,999	1500	17.4	1031	16.9
>= $150,000	2006	23.3	1167	19.1
Non-response	0	0.0	0	0.0
Employment				
Working full-time	3888	45.1	3377	55.4
Working part-time	1132	13.1	1055	17.3
Not working	3600	41.8	1667	27.3
Non-response	0	0.0	0	0.0
Census division				
New England	412	4.8	258	4.2
Mid-Atlantic	1090	12.6	747	12.3
East-North Central	1267	14.7	840	13.8
West-North Central	604	7.0	420	6.9
South Atlantic	1714	19.9	1232	20.2
East-South Central	465	5.4	432	7.1
West-South Central	904	10.5	840	13.8
Mountain	745	8.6	426	7.0
Pacific	1419	16.5	904	14.8
Non-response	0	0.0	0	0

Table S3, continued.

Unweighted mean (SD) ages were 53.8 (17.2) for respondents and 42.9 (16.2) for nonrespondents.

This table was previously published in the supplement to Wintemute GJ, Robinson SL, Crawford A, Tancredi D, Schleimer JP, Tomsich EA, Reeping PM, Shev AB, Pear VA. Views of democracy and society and support for political violence in the USA: findings from a nationally representative survey. Injury Epidemiology. 2023;10(1):45. DOI: 10.1186/s40621-023-00456-3.

Table S4. Approval of specified organizations and movements and justification for violence to advance 8 additional specific political objectives

What do you think about the use of force or violence in the following situations?	Approval of Organizations and Movements							
	Non-Approval		Weak Approval		Moderate Approval		Strong Approval	
	Unweighted n	Weighted % (95% CI)	Unweighted n	Weighted % (95% CI)	Unweighted n	Weighted % (95% CI)	Unweighted n	Weighted % (95% CI)
To stop voter fraud								
Never justified	1612	86.4 (84.7,88.2)	247	63.5 (58.1,68.9)	57	41.6 (31.8,51.5)	8	21.6 (5.8,37.3)
Sometimes justified	173	9.4 (7.9,10.9)	75	20.0 (15.6,24.5)	35	28.7 (19.7,37.8)	12	25.2 (11.0,39.4)
Usually or always justified	71	3.7 (2.7,4.6)	63	16.3 (12.0,20.5)	38	29.7 (20.1,39.2)	23	53.2 (36.0,70.5)
Adjusted prevalence difference* (95% CI; q-value)	Referent		10.2 (6.2,14.3; <0.001)		21.8 (12.8,30.7; <0.001)		45.3 (29.2,61.4; <0.001)	
To stop voter intimidation								
Never justified	1161	63.1 (60.6,65.5)	218	56.3 (50.8,61.9)	53	47.1 (36.6,57.6)	8	22.7 (7.0,38.5)
Sometimes justified	540	29.2 (26.9,31.5)	102	25.1 (20.4,29.9)	39	31.0 (21.4,40.6)	15	45.4 (26.4,64.4)
Usually or always justified	153	7.6 (6.4,8.9)	72	18.5 (14.2,22.9)	25	21.9 (13.1,30.7)	10	27.4 (10.9,43.9)
Adjusted prevalence difference* (95% CI; q-value)	Referent		10.3 (5.8,14.8; <0.001)		14.4 (5.6,23.2; 0.02)		22.1 (4.8,39.5; 0.09)	
To reinforce the police								
Never justified	1014	56.3 (53.8,58.8)	105	28.0 (23.0,33.1)	17	18.5 (9.8,27.2)	2	4.4 (-1.9,10.6)
Sometimes justified	690	35.8 (33.4,38.3)	177	45.2 (39.7,50.7)	52	38.6 (28.8,48.4)	12	41.9 (21.5,62.2)
Usually or always justified	159	7.7 (6.4,8.9)	109	26.8 (21.8,31.8)	50	42.9 (32.5,53.3)	21	53.8 (33.7,73.8)
Adjusted prevalence difference* (95% CI; q-value)	Referent		16.6 (11.6,21.6; <0.001)		33.8 (23.3,44.3; <0.001)		46.0 (26.2,65.7; <0.001)	
To stop police violence								
Never justified	908	47.2 (44.7,49.7)	169	41.5 (36.0,47.0)	52	38.3 (28.7,48.0)	13	30.2 (14.8,45.6)
Sometimes justified	771	42.4 (39.9,44.9)	149	38.6 (33.1,44.0)	52	40.5 (30.5,50.5)	16	40.8 (24.1,57.4)
Usually or always justified	169	10.2 (8.6,11.8)	69	19.9 (15.2,24.7)	24	21.2 (12.6,29.8)	12	25.0 (11.3,38.7)
Adjusted prevalence difference* (95% CI; q-value)	Referent		8.2 (3.3,13.1; 0.02)		4.8 (-3.4,13.0; 0.49)		8.4 (-7.1,23.9; 0.51)	
To stop illegal immigration								
Never justified	1477	80.0 (78.0,82.1)	166	45.1 (39.6,50.7)	38	29.3 (20.6,38.1)	4	11.0 (0.6,21.3)
Sometimes justified	304	16.0 (14.2,17.9)	134	32.3 (27.3,37.4)	47	34.7 (25.2,44.2)	14	42.3 (24.1,60.6)
Usually or always justified	76	3.8 (2.9,4.8)	90	22.5 (17.8,27.2)	47	36.0 (26.6,45.4)	18	42.4 (24.6,60.2)
Adjusted prevalence difference* (95% CI; q-value)	Referent		17.2 (12.5,21.9; <0.001)		30.5 (21.2,39.7; <0.001)		38.9 (20.9,57.0; <0.001)	
To keep borders open								
Never justified	1342	72.2 (69.9,74.4)	234	58.0 (52.5,63.6)	57	45.4 (34.7,56.0)	16	32.8 (17.0,48.6)
Sometimes justified	438	23.3 (21.2,25.5)	104	28.4 (23.3,33.5)	30	26.8 (17.1,36.5)	11	33.9 (15.7,52.1)
Usually or always justified	75	4.1 (3.1,5.1)	50	13.6 (9.7,17.4)	26	26.9 (16.6,37.3)	13	33.3 (16.3,50.2)
Adjusted prevalence difference* (95% CI; q-value)	Referent		8.9 (4.7,13.1; <0.001)		21.2 (11.3,31.0; <0.001)		27.6 (10.9,44.3; 0.01)	
To stop a protest								
Never justified	1289	72.0 (69.7,74.2)	178	51.2 (45.4,56.9)	48	36.2 (26.9,45.6)	5	16.9 (2.8,30.9)
Sometimes justified	496	25.9 (23.7,28.1)	148	37.8 (32.4,43.2)	62	46.4 (36.4,56.5)	14	30.6 (14.4,46.9)
Usually or always justified	37	1.7 (1.1,2.3)	40	11.0 (7.5,14.5)	18	17.4 (8.6,26.1)	16	47.8 (29.2,66.3)
Adjusted prevalence difference* (95% CI; q-value)	Referent		7.9 (4.4,11.5; <0.001)		12.4 (4.7,20.2; 0.02)		44.1 (26.5,61.6; <0.001)	
To support a protest								
Never justified	1599	82.7 (80.8,84.7)	303	71.7 (66.7,76.7)	67	50.5 (40.1,61.0)	11	26.6 (11.3,41.8)
Sometimes justified	262	15.5 (13.6,17.4)	82	21.1 (16.6,25.6)	27	26.3 (16.5,36.1)	13	30.2 (13.7,46.8)
Usually or always justified	27	1.6 (1.0,2.3)	27	7.2 (4.3,10.1)	25	23.2 (14.4,32.0)	17	43.2 (25.5,60.9)
Adjusted prevalence difference* (95% CI; q-value)	Referent		5.2 (2.2,8.2; 0.007)		20.1 (11.5,28.6; <0.001)		37.8 (20.6,55.0; <0.001)	

*	Prevalence differences are adjusted for age, race and ethnicity, gender, education, income, Census division, and rurality and are for the usually or always justified comparison. They are expressed in absolute percentage points. Q-values represent the probability that the given difference would be a false discovery; they represent the expected proportion of “false positives” that would be seen among the collection of all differences whose q-values were at or below the given q-value.

Table S5. Approval of specified organizations and movements and personal willingness to engage in political violence, by target of violence

In a situation where you think force or violence is justified to advance an important political objective…How willing would you personally be to use force or violence against a person because they are…	Approval of Organizations and Movements							
	Non-Approval		Weak Approval		Moderate Approval		Strong Approval	
	Unweighted n	Weighted % (95% CI)	Unweighted n	Weighted % (95% CI)	Unweighted n	Weighted % (95% CI)	Unweighted n	Weighted % (95% CI)
An elected federal or state government official								
Not asked the question	1060	28.3 (26.7,29.9)	82	10.0 (7.6,12.4)	18	8.2 (4.0,12.3)	4	5.4 (-0.2,11.0)
Not willing	2535	67.6 (65.9,69.3)	619	78.9 (75.5,82.2)	153	56.4 (49.1,63.8)	33	42.5 (29.8,55.2)
Sometimes willing	94	2.9 (2.3,3.6)	52	7.1 (4.9,9.3)	48	21.7 (15.7,27.8)	17	25.1 (13.6,36.7)
Very or completely willing	22	0.8 (0.4,1.1)	19	3.2 (1.7,4.7)	21	11.5 (5.9,17.1)	22	26.0 (15.0,37.0)
Adjusted prevalence difference* (95% CI; q-value)	Referent		2.4 (0.8,4.0; 0.03)		9.9 (4.6,15.2; 0.004)		23.4 (12.7,34.0; <0.001)	
An elected local government official								
Not asked the question	1060	28.3 (26.7,29.9)	82	10.0 (7.6,12.4)	18	8.2 (4.0,12.3)	4	5.4 (-0.2,11.0)
Not willing	2546	67.9 (66.2,69.5)	627	80.5 (77.3,83.7)	157	59.0 (51.8,66.3)	34	44.7 (31.9,57.5)
Sometimes willing	79	2.6 (2.0,3.2)	44	6.3 (4.3,8.2)	48	20.8 (14.9,26.7)	14	19.9 (9.0,30.8)
Very or completely willing	20	0.6 (0.3,0.9)	18	2.5 (1.3,3.8)	20	11.1 (5.8,16.4)	23	26.9 (16.4,37.4)
Adjusted prevalence difference* (95% CI; q-value)	Referent		2.0 (0.7,3.4; 0.03)		10.1 (5.1,15.1; 0.001)		26.2 (15.2,37.2; <0.001)	
An election worker, such as a poll worker or vote counter								
Not asked the question	1060	28.3 (26.7,29.9)	82	10.0 (7.6,12.4)	18	8.2 (4.0,12.3)	4	5.4 (-0.2,11.0)
Not willing	2605	70.1 (68.5,71.7)	649	82.7 (79.5,85.8)	175	64.8 (57.6,72.1)	33	41.8 (29.1,54.4)
Sometimes willing	27	0.7 (0.4,1.0)	25	3.8 (2.0,5.6)	37	17.8 (12.1,23.6)	15	19.6 (9.0,30.2)
Very or completely willing	14	0.4 (0.2,0.6)	16	2.8 (1.3,4.3)	15	8.7 (3.8,13.5)	24	32.2 (20.4,44.1)
Adjusted prevalence difference* (95% CI; q-value)	Referent		2.6 (1.0,4.1; 0.02)		7.9 (3.2,12.5; 0.01)		31.2 (19.6,42.7; <0.001)	
A public health official								
Not asked the question	1060	28.3 (26.7,29.9)	82	10.0 (7.6,12.4)	18	8.2 (4.0,12.3)	4	5.4 (-0.2,11.0)
Not willing	2600	69.8 (68.2,71.4)	628	81.0 (77.8,84.1)	167	63.1 (56.0,70.3)	31	38.1 (25.7,50.4)
Sometimes willing	35	1.0 (0.6,1.3)	40	5.4 (3.6,7.2)	39	16.9 (11.5,22.2)	17	25.5 (13.8,37.2)
Very or completely willing	10	0.4 (0.1,0.6)	17	2.4 (1.1,3.7)	19	10.9 (5.7,16.0)	22	27.2 (16.0,38.4)
Adjusted prevalence difference* (95% CI; q-value)	Referent		2.2 (0.9,3.6; 0.01)		10.2 (5.3,15.1; <0.001)		27.1 (15.7,38.5; <0.001)	
A member of the military or National Guard								
Not asked the question	1060	28.3 (26.7,29.9)	82	10.0 (7.6,12.4)	18	8.2 (4.0,12.3)	4	5.4 (-0.2,11.0)
Not willing	2535	67.7 (66.1,69.4)	632	81.1 (78.0,84.2)	169	61.3 (54.0,68.7)	31	39.1 (26.6,51.7)
Sometimes willing	94	2.9 (2.2,3.5)	42	5.6 (3.9,7.4)	35	15.8 (10.5,21.2)	17	21.1 (11.1,31.0)
Very or completely willing	19	0.6 (0.3,0.9)	17	2.7 (1.3,4.1)	21	13.3 (7.3,19.3)	24	33.5 (21.1,45.8)
Adjusted prevalence difference* (95% CI; q-value)	Referent		1.5 (0.1,2.9; 0.18)		11.4 (5.8,16.9; <0.001)		30.5 (18.4,42.6; <0.001)	
A police officer								
Not asked the question	1060	28.3 (26.7,29.9)	82	10.0 (7.6,12.4)	18	8.2 (4.0,12.3)	4	5.4 (-0.2,11.0)
Not willing	2491	66.0 (64.3,67.8)	615	78.3 (74.9,81.6)	167	61.5 (54.2,68.8)	39	53.5 (40.8,66.2)
Sometimes willing	124	4.0 (3.2,4.8)	47	6.4 (4.5,8.3)	38	16.4 (11.1,21.8)	12	16.4 (6.5,26.2)
Very or completely willing	33	1.1 (0.7,1.5)	24	4.0 (2.2,5.8)	22	13.4 (7.4,19.3)	21	23.8 (13.7,33.8)
Adjusted prevalence difference* (95% CI; q-value)	Referent		2.4 (0.7,4.1; 0.05)		10.6 (5.1,16.1; 0.002)		19.9 (9.7,30.2; 0.002)	
A person who does not share your race or ethnicity								
Not asked the question	1060	28.3 (26.7,29.9)	82	10.0 (7.6,12.4)	18	8.2 (4.0,12.3)	4	5.4 (-0.2,11.0)
Not willing	2610	70.0 (68.3,71.6)	646	82.5 (79.5,85.6)	174	65.0 (57.8,72.1)	28	34.2 (22.2,46.2)
Sometimes willing	27	0.8 (0.5,1.1)	28	4.1 (2.5,5.7)	38	17.0 (11.6,22.4)	17	24.5 (13.0,36.1)
Very or completely willing	11	0.4 (0.1,0.7)	15	2.4 (1.1,3.7)	16	9.7 (4.6,14.7)	26	34.1 (21.9,46.3)
Adjusted prevalence difference* (95% CI; q-value)	Referent		1.6 (0.3,2.8; 0.10)		8.2 (3.5,13.0; 0.01)		32.5 (20.6,44.5; <0.001)	
A person who does not share your religion								
Not asked the question	1060	28.3 (26.7,29.9)	82	10.0 (7.6,12.4)	18	8.2 (4.0,12.3)	4	5.4 (-0.2,11.0)
Not willing	2608	69.9 (68.2,71.5)	643	81.6 (78.5,84.8)	178	66.6 (59.5,73.7)	34	45.2 (32.4,58.0)
Sometimes willing	22	0.7 (0.4,1.1)	27	4.4 (2.7,6.2)	32	15.2 (9.8,20.5)	15	21.3 (10.0,32.5)
Very or completely willing	14	0.5 (0.2,0.7)	17	2.9 (1.4,4.3)	14	8.5 (3.7,13.3)	22	26.0 (15.5,36.5)
Adjusted prevalence difference* (95% CI; q-value)	Referent		2.2 (0.8,3.6; 0.02)		7.4 (2.7,12.0; 0.02)		24.9 (14.3,35.4; <0.001)	
A person who does not share your political beliefs								
Not asked the question	1060	28.3 (26.7,29.9)	82	10.0 (7.6,12.4)	18	8.2 (4.0,12.3)	4	5.4 (-0.2,11.0)
Not willing	2571	68.7 (67.0,70.3)	634	81.8 (78.7,84.8)	173	64.9 (57.8,72.1)	32	42.7 (30.0,55.4)
Sometimes willing	67	2.1 (1.6,2.7)	42	5.2 (3.6,6.8)	37	18.0 (12.1,24.0)	17	23.1 (11.7,34.4)
Very or completely willing	12	0.4 (0.2,0.6)	13	2.3 (0.9,3.6)	14	7.2 (3.0,11.4)	23	27.8 (16.6,39.0)
Adjusted prevalence difference* (95% CI; q-value)	Referent		1.6 (0.3,2.9; 0.16)		6.5 (2.6,10.4; 0.02)		27.4 (16.3,38.4; <0.001)	

* Prevalence differences are adjusted for age, race and ethnicity, gender, education, income, Census division, and rurality and are for the very or completely willing comparison. They are expressed in absolute percentage points. Q-values represent the probability that the given difference would be a false discovery; they represent the expected proportion of “false positives” that would be seen among the collection of all differences whose q-values were at or below the given q-value.

Table S6. Approval of specified organizations and movements and justification for violence in non-political situations

What do you think about the use of force or violence in the following situations?	Approval of Organizations and Movements							
	Non-Approval		Weak Approval		Moderate Approval		Strong Approval	
	Unweighted n	Weighted % (95% CI)	Unweighted n	Weighted % (95% CI)	Unweighted n	Weighted % (95% CI)	Unweighted n	Weighted % (95% CI)
In self defense								
Never justified	52	1.5 (1.1,2.0)	15	2.1 (1.0,3.3)	6	2.8 (0.5,5.1)	3	3.7 (-0.6,7.9)
Sometimes justified	937	25.1 (23.5,26.6)	135	18.5 (15.3,21.7)	35	20.1 (13.4,26.9)	16	28.7 (16.0,41.5)
Usually or always justified	2727	73.1 (71.5,74.7)	627	79.2 (75.9,82.5)	206	77.1 (70.2,83.9)	57	65.4 (52.4,78.4)
Adjusted prevalence difference* (95% CI; q-value)	Referent		6.4 (2.8,10.1; 0.005)		5.7 (-0.8,12.2; 0.27)		-1.8 (-14.4,10.8; 0.92)	
To prevent someone from injuring or killing another person								
Never justified	48	1.5 (1.0,1.9)	7	2.3 (1.1,3.4)	7	4.3 (0.7,7.9)	6	7.3 (1.1,13.5)
Sometimes justified	763	21.0 (19.5,22.4)	29	15.7 (12.8,18.6)	29	14.6 (9.3,20.0)	18	30.5 (17.7,43.3)
Usually or always justified	2891	76.9 (75.4,78.4)	211	81.8 (78.7,84.8)	211	81.0 (74.9,87.1)	52	60.0 (46.9,73.0)
Adjusted prevalence difference* (95% CI; q-value)	Referent		5.0 (1.5,8.4; 0.03)		6.6 (0.4,12.8; 0.12)		-9.5 (-22.0,3.0; 0.34)	
To prevent someone from injuring or killing themselves								
Never justified	240	6.9 (6.0,7.9)	44	5.8 (3.9,7.8)	14	7.3 (3.1,11.5)	10	12.3 (3.4,21.2)
Sometimes justified	1351	37.5 (35.7,39.2)	241	31.6 (27.9,35.3)	75	29.7 (23.1,36.3)	23	32.1 (19.8,44.4)
Usually or always justified	2117	55.1 (53.3,56.9)	493	62.6 (58.7,66.4)	157	62.8 (55.7,69.9)	43	53.4 (40.5,66.3)
Adjusted prevalence difference* (95% CI; q-value)	Referent		6.5 (2.2,10.8; 0.03)		10.2 (2.8,17.6; 0.05)		5.9 (-7.1,18.9; 0.52)	
To prevent harm or damage to property								
Never justified	777	21.4 (19.9,22.9)	79	9.8 (7.5,12.1)	23	10.9 (6.2,15.7)	6	9.1 (1.3,16.9)
Sometimes justified	2016	54.3 (52.5,56.1)	359	46.5 (42.6,50.5)	96	36.7 (29.7,43.6)	26	35.0 (22.6,47.5)
Usually or always justified	914	23.8 (22.3,25.3)	339	43.6 (39.6,47.5)	128	52.4 (45.1,59.6)	43	51.3 (38.5,64.2)
Adjusted prevalence difference* (95% CI; q-value)	Referent		16.3 (12.1,20.6; <0.001)		25.7 (18.3,33.1; <0.001)		28.6 (16.4,40.8; <0.001)	
To win an argument								
Never justified	3497	93.5 (92.6,94.4)	643	82.0 (79.0,85.0)	171	64.2 (57.0,71.4)	24	27.7 (16.6,38.9)
Sometimes justified	161	4.4 (3.6,5.2)	99	12.5 (10.0,15.1)	49	22.6 (16.2,29.1)	25	33.5 (21.2,45.9)
Usually or always justified	53	1.7 (1.2,2.2)	36	5.5 (3.5,7.4)	26	13.0 (7.8,18.2)	26	34.3 (22.2,46.4)
Adjusted prevalence difference* (95% CI; q-value)	Referent		2.9 (0.9,4.9; 0.04)		8.3 (3.4,13.2; 0.01)		30.0 (18.1,41.8; <0.001)	
In response to an insult								
Never justified	3360	89.1 (88.0,90.3)	629	78.4 (75.1,81.8)	176	66.7 (59.7,73.8)	27	32.5 (20.6,44.4)
Sometimes justified	275	8.1 (7.1,9.2)	117	17.1 (14.0,20.3)	45	20.5 (14.6,26.4)	19	23.7 (13.2,34.2)
Usually or always justified	74	2.3 (1.7,2.9)	32	4.4 (2.8,6.0)	25	12.6 (7.2,18.0)	30	41.6 (28.9,54.4)
Adjusted prevalence difference* (95% CI; q-value)	Referent		1.2 (-0.6,2.9; 0.43)		7.3 (2.4,12.3; 0.04)		35.6 (22.7,48.6; <0.001)	
To get respect								
Never justified	3517	93.9 (93.0,94.8)	672	84.8 (81.9,87.7)	179	65.7 (58.5,73.0)	27	27.5 (16.7,38.4)
Sometimes justified	143	4.1 (3.4,4.8)	74	10.8 (8.2,13.4)	36	19.5 (13.0,26.0)	15	19.3 (9.5,29.1)
Usually or always justified	53	1.6 (1.1,2.1)	31	4.3 (2.7,5.9)	31	14.4 (9.2,19.6)	34	50.9 (38.1,63.7)
Adjusted prevalence difference* (95% CI; q-value)	Referent		1.8 (0.1,3.5; 0.18)		9.9 (5.2,14.6; <0.001)		46.0 (33.4,58.6; <0.001)	

* Prevalence differences are adjusted for age, race and ethnicity, gender, education, income, Census division, and rurality and are for the usually or always justified comparison. They are expressed in absolute percentage points. Q-values represent the probability that the given difference would be a false discovery; they represent the expected proportion of “false positives” that would be seen among the collection of all differences whose q-values were at or below the given q-value.

Table S7. Approval of individual organizations and movements and justification for political violence, in general and to advance 9 specific political objectives

What do you think about the use of force or violence in the following situations?	Proud Boys					
	Do Not Approve		Somewhat Approve		Strongly/Very Strongly Approve	
	Unweighted n	Weighted % (95% CI)	Unweighted n	Weighted % (95% CI)	Unweighted n	Weighted % (95% CI)
In general...to advance an important political objective that you support						
Never justified	4132	82.0 (80.7,83.3)	225	65.5 (59.3,71.7)	85	45.2 (36.5,53.9)
Sometimes justified	700	16.6 (15.4,17.9)	84	27.7 (22.0,33.4)	37	22.9 (15.4,30.5)
Usually or always justified	46	1.3 (0.9,1.7)	15	6.8 (2.8,10.8)	39	31.8 (22.8,40.9)
Adjusted prevalence difference* (95% CI; q-value)	Referent		4.7 (0.7,8.7; 0.11)		28.4 (19.9,36.9; <0.001)	
Usually or always justified to advance at least 1 of 17 objectives	1195	24.7 (23.3,26.1)	186	59.4 (53.3,65.5)	113	72.4 (64.8,80.1)
Adjusted prevalence difference* (95% CI; q-value)	Referent		29.0 (22.7,35.3; <0.001)		41.9 (34.1,49.8; <0.001)	
To return Donald Trump to the presidency this year						
Never justified	4644	94.7 (94.0,95.5)	240	71.1 (65.0,77.2)	81	43.7 (35.0,52.5)
Sometimes justified	103	2.4 (1.8,2.9)	40	14.0 (9.2,18.8)	24	17.2 (9.9,24.5)
Usually or always justified	108	2.4 (1.9,2.9)	42	14.4 (9.5,19.4)	54	37.0 (28.3,45.7)
Adjusted prevalence difference* (95% CI; q-value)	Referent		9.9 (5.2,14.6; <0.001)		33.1 (24.5,41.7; <0.001)	
To stop an election from being stolen						
Never justified	4038	82.7 (81.5,83.9)	147	44.5 (38.3,50.7)	55	31.1 (23.1,39.1)
Sometimes justified	591	12.0 (11.0,13.0)	103	30.2 (24.5,35.8)	44	28.2 (20.1,36.4)
Usually or always justified	228	4.9 (4.2,5.6)	72	24.9 (19.1,30.7)	60	38.6 (29.8,47.4)
Adjusted prevalence difference* (95% CI; q-value)	Referent		17.4 (11.8,23.0; <0.001)		32.1 (23.5,40.7; <0.001)	
To stop people who do not share my beliefs from voting						
Never justified	4716	95.8 (95.1,96.5)	274	80.0 (74.4,85.6)	113	62.5 (53.5,71.5)
Sometimes justified	90	2.3 (1.8,2.8)	33	12.7 (8.1,17.3)	17	13.0 (6.7,19.2)
Usually or always justified	55	1.5 (1.1,2.0)	15	6.9 (3.1,10.7)	29	22.5 (14.4,30.5)
Adjusted prevalence difference* (95% CI; q-value)	Referent		4.3 (0.5,8.1; 0.12)		18.9 (11.2,26.7; <0.001)	
To prevent discrimination based on race or ethnicity						
Never justified	3250	64.5 (63.0,66.0)	176	50.5 (44.2,56.7)	69	39.1 (30.5,47.7)
Sometimes justified	1271	27.0 (25.6,28.4)	101	31.2 (25.3,37.0)	46	29.4 (21.1,37.7)
Usually or always justified	334	8.0 (7.1,8.9)	46	18.2 (12.9,23.4)	43	28.9 (20.7,37.1)
Adjusted prevalence difference* (95% CI; q-value)	Referent		7.0 (1.8,12.2; 0.05)		17.2 (9.1,25.4; <0.001)	
To preserve an American way of life based on Western European traditions						
Never justified	4000	82.9 (81.8,84.1)	154	47.7 (41.4,53.9)	66	38.0 (29.5,46.5)
Sometimes justified	702	13.2 (12.2,14.2)	122	35.9 (30.0,41.9)	46	29.4 (21.1,37.6)
Usually or always justified	145	3.2 (2.6,3.8)	46	15.8 (10.9,20.7)	45	29.6 (21.2,37.9)
Adjusted prevalence difference* (95% CI; q-value)	Referent		10.6 (5.9,15.4; <0.001)		25.6 (17.3,34.0; <0.001)	
To preserve the American way of life I believe in						
Never justified	3072	64.3 (62.8,65.8)	92	29.0 (23.4,34.6)	37	21.7 (14.7,28.6)
Sometimes justified	1400	27.5 (26.1,28.9)	145	43.3 (37.1,49.5)	53	35.4 (26.7,44.1)
Usually or always justified	391	7.9 (7.0,8.7)	87	27.7 (21.9,33.6)	70	41.6 (32.8,50.4)
Adjusted prevalence difference* (95% CI; q-value)	Referent		16.5 (10.8,22.3; <0.001)		31.2 (22.7,39.7; <0.001)	
To oppose Americans who do not share my beliefs						
Never justified	4560	92.2 (91.2,93.1)	251	73.7 (68.0,79.5)	106	58.2 (49.1,67.4)
Sometimes justified	254	6.0 (5.2,6.8)	57	18.0 (13.3,22.8)	20	13.5 (7.1,19.9)
Usually or always justified	55	1.6 (1.1,2.1)	16	8.2 (4.0,12.4)	34	26.9 (18.3,35.5)
Adjusted prevalence difference* (95% CI; q-value)	Referent		5.4 (1.4,9.5; 0.05)		22.9 (14.8,31.0; <0.001)	
To oppose the government when it does not share my beliefs						
Never justified	4250	85.3 (84.1,86.5)	195	56.5 (50.2,62.8)	82	47.2 (38.3,56.1)
Sometimes justified	530	12.4 (11.3,13.5)	101	32.3 (26.3,38.3)	38	21.5 (14.2,28.9)
Usually or always justified	79	1.8 (1.4,2.3)	25	10.5 (6.1,14.9)	39	29.2 (20.7,37.7)
Adjusted prevalence difference* (95% CI; q-value)	Referent		7.6 (3.3,11.9; 0.005)		25.7 (17.5,33.8; <0.001)	
To oppose the government when it tries to take private land for public purposes						
Never justified	3452	69.4 (67.9,70.8)	104	32.9 (27.1,38.8)	53	29.4 (21.7,37.1)
Sometimes justified	1156	24.4 (23.0,25.8)	146	40.4 (34.4,46.5)	43	24.9 (17.4,32.3)
Usually or always justified	244	5.7 (4.9,6.4)	72	26.2 (20.4,32.1)	63	43.7 (34.6,52.7)
Adjusted prevalence difference* (95% CI; q-value)	Referent		17.2 (11.4,23.0; <0.001)		35.0 (26.2,43.7; <0.001)	

Table S7, continued.

What do you think about the use of force or violence in the following situations?	Oath Keepers					
	Do Not Approve		Somewhat Approve		Strongly/Very Strongly Approve	
	Unweighted n	Weighted % (95% CI)	Unweighted n	Weighted % (95% CI)	Unweighted n	Weighted % (95% CI)
In general...to advance an important political objective that you support						
Never justified	2969	83.7 (82.3,85.2)	223	66.3 (60.2,72.5)	122	50.3 (42.6,57.9)
Sometimes justified	444	14.7 (13.3,16.1)	79	24.9 (19.5,30.3)	55	26.3 (19.4,33.2)
Usually or always justified	31	1.5 (0.9,2.1)	19	8.8 (4.2,13.3)	41	23.5 (16.4,30.5)
Adjusted prevalence difference* (95% CI; q-value)	Referent		5.1 (1.2,9.1; 0.06)		18.8 (12.3,25.4; <0.001)	
Usually or always justified to advance at least 1 of 17 objectives	768	22.7 (21.1,24.2)	173	55.7 (49.5,62.0)	156	72.2 (65.5,79.0)
Adjusted prevalence difference* (95% CI; q-value)	Referent		27.2 (20.8,33.6; <0.001)		45.5 (38.3,52.6; <0.001)	
To return Donald Trump to the presidency this year						
Never justified	3289	94.5 (93.5,95.5)	245	74.9 (69.5,80.4)	131	54.5 (46.8,62.2)
Sometimes justified	71	2.6 (1.9,3.3)	39	13.1 (8.8,17.3)	32	15.5 (9.7,21.4)
Usually or always justified	66	2.3 (1.7,3.0)	36	11.8 (7.6,15.9)	51	27.5 (20.4,34.7)
Adjusted prevalence difference* (95% CI; q-value)	Referent		7.0 (3.2,10.8; 0.004)		23.9 (16.9,30.9; <0.001)	
To stop an election from being stolen						
Never justified	2874	82.8 (81.3,84.2)	166	51.2 (44.9,57.5)	93	40.8 (33.4,48.2)
Sometimes justified	415	12.2 (11.0,13.4)	90	27.9 (22.3,33.5)	48	22.7 (16.2,29.3)
Usually or always justified	138	4.5 (3.7,5.4)	63	20.5 (15.2,25.7)	73	34.4 (27.1,41.7)
Adjusted prevalence difference* (95% CI; q-value)	Referent		13.5 (8.4,18.6; <0.001)		28.2 (20.8,35.6; <0.001)	
To stop people who do not share my beliefs from voting						
Never justified	3341	95.9 (95.0,96.8)	267	79.3 (73.7,84.8)	158	64.1 (56.3,71.8)
Sometimes justified	58	2.4 (1.7,3.1)	31	11.4 (7.1,15.7)	24	13.2 (7.9,18.5)
Usually or always justified	33	1.3 (0.8,1.8)	22	9.1 (5.0,13.2)	33	20.9 (13.9,28.0)
Adjusted prevalence difference* (95% CI; q-value)	Referent		5.4 (1.7,9.0; 0.03)		17.1 (10.4,23.7; <0.001)	
To prevent discrimination based on race or ethnicity						
Never justified	2340	66.4 (64.6,68.1)	180	51.8 (45.5,58.1)	110	45.8 (38.2,53.3)
Sometimes justified	882	26.4 (24.8,28.1)	101	32.7 (26.8,38.7)	41	18.0 (12.0,24.0)
Usually or always justified	202	6.6 (5.6,7.6)	38	14.9 (9.8,19.9)	63	34.0 (26.6,41.4)
Adjusted prevalence difference* (95% CI; q-value)	Referent		5.4 (0.8,10.0; 0.12)		23.6 (16.2,31.0; <0.001)	
To preserve an American way of life based on Western European traditions						
Never justified	2870	83.7 (82.4,85.1)	151	47.4 (41.1,53.7)	88	41.5 (34.0,49.0)
Sometimes justified	464	12.6 (11.5,13.8)	125	36.1 (30.3,42.0)	70	28.4 (21.7,35.2)
Usually or always justified	93	3.1 (2.3,3.9)	39	14.2 (9.2,19.3)	56	27.7 (20.8,34.6)
Adjusted prevalence difference* (95% CI; q-value)	Referent		8.1 (3.6,12.6; 0.006)		23.4 (16.3,30.5; <0.001)	
To preserve the American way of life I believe in						
Never justified	2252	65.8 (64.0,67.5)	93	30.8 (24.9,36.6)	54	24.5 (18.1,30.9)
Sometimes justified	952	27.2 (25.6,28.9)	148	42.4 (36.2,48.5)	68	34.2 (26.7,41.7)
Usually or always justified	228	6.6 (5.7,7.6)	80	26.8 (21.0,32.7)	94	40.1 (32.7,47.4)
Adjusted prevalence difference* (95% CI; q-value)	Referent		15.8 (10.3,21.2; <0.001)		32.3 (24.9,39.7; <0.001)	
To oppose Americans who do not share my beliefs						
Never justified	3242	92.6 (91.5,93.7)	245	73.9 (68.2,79.7)	142	57.1 (49.4,64.9)
Sometimes justified	161	5.6 (4.7,6.6)	64	20.2 (15.1,25.2)	30	15.6 (9.9,21.4)
Usually or always justified	34	1.5 (0.9,2.1)	12	5.9 (2.1,9.6)	44	26.0 (18.7,33.3)
Adjusted prevalence difference* (95% CI; q-value)	Referent		2.4 (-1.0,5.8; 0.49)		21.6 (14.7,28.6; <0.001)	
To oppose the government when it does not share my beliefs						
Never justified	3061	86.9 (85.5,88.3)	204	62.3 (56.1,68.4)	111	46.7 (39.2,54.3)
Sometimes justified	316	10.8 (9.6,12.1)	90	26.9 (21.5,32.3)	61	27.8 (20.9,34.7)
Usually or always justified	50	1.7 (1.2,2.2)	27	10.8 (6.2,15.5)	42	23.3 (16.5,30.1)
Adjusted prevalence difference* (95% CI; q-value)	Referent		7.0 (2.9,11.1; 0.008)		20.0 (13.5,26.6; <0.001)	
To oppose the government when it tries to take private land for public purposes						
Never justified	2562	73.0 (71.3,74.6)	121	38.5 (32.4,44.7)	65	28.8 (22.0,35.5)
Sometimes justified	720	21.6 (20.0,23.1)	142	41.2 (35.1,47.3)	69	28.0 (21.4,34.5)
Usually or always justified	141	4.9 (4.0,5.7)	54	19.4 (13.9,24.8)	80	40.9 (33.3,48.6)
Adjusted prevalence difference* (95% CI; q-value)	Referent		10.2 (5.0,15.4; 0.002)		32.5 (24.9,40.1; <0.001)	

Table S7, continued.

What do you think about the use of force or violence in the following situations?	Three Percenters					
	Do Not Approve		Somewhat Approve		Strongly/Very Strongly Approve	
	Unweighted n	Weighted % (95% CI)	Unweighted n	Weighted % (95% CI)	Unweighted n	Weighted % (95% CI)
In general...to advance an important political objective that you support						
Never justified	2048	82.1 (80.3,83.9)	120	53.1 (45.2,61.1)	48	38.1 (28.4,47.8)
Sometimes justified	337	15.6 (13.9,17.2)	57	32.3 (24.9,39.8)	37	30.0 (20.7,39.2)
Usually or always justified	35	2.2 (1.4,3.1)	20	14.5 (7.9,21.2)	33	31.9 (22.0,41.9)
Adjusted prevalence difference* (95% CI; q-value)	Referent		9.9 (3.6,16.2; 0.02)		25.5 (15.8,35.1; <0.001)	
Usually or always justified to advance at least 1 of 17 objectives	637	27.5 (25.5,29.6)	113	58.9 (51.2,66.6)	88	77.3 (69.2,85.3)
Adjusted prevalence difference* (95% CI; q-value)	Referent		25.0 (16.7,33.4; <0.001)		44.4 (35.7,53.2; <0.001)	
To return Donald Trump to the presidency this year						
Never justified	2263	92.4 (91.0,93.7)	138	66.8 (59.2,74.5)	56	40.4 (30.7,50.1)
Sometimes justified	64	3.2 (2.3,4.1)	28	15.3 (9.5,21.1)	24	21.4 (12.6,30.2)
Usually or always justified	76	3.7 (2.7,4.7)	31	17.9 (11.5,24.3)	36	35.6 (25.5,45.6)
Adjusted prevalence difference* (95% CI; q-value)	Referent		11.2 (4.9,17.5; 0.005)		29.1 (19.2,39.0; <0.001)	
To stop an election from being stolen						
Never justified	1971	80.5 (78.6,82.3)	95	45.1 (37.3,53.0)	45	35.7 (26.1,45.3)
Sometimes justified	287	11.6 (10.2,13.0)	59	32.0 (24.5,39.6)	28	24.3 (15.3,33.3)
Usually or always justified	149	7.4 (6.1,8.8)	43	22.8 (16.2,29.4)	44	38.3 (28.4,48.3)
Adjusted prevalence difference* (95% CI; q-value)	Referent		11.7 (4.9,18.5; 0.01)		27.9 (17.7,38.1; <0.001)	
To stop people who do not share my beliefs from voting						
Never justified	2315	94.5 (93.4,95.7)	146	66.6 (58.7,74.6)	65	47.0 (36.9,57.1)
Sometimes justified	55	2.9 (2.0,3.8)	34	21.2 (14.2,28.2)	16	15.6 (8.1,23.1)
Usually or always justified	40	2.1 (1.4,2.9)	17	12.1 (6.3,18.0)	36	35.7 (25.5,46.0)
Adjusted prevalence difference* (95% CI; q-value)	Referent		7.7 (2.2,13.3; 0.04)		29.7 (19.9,39.5; <0.001)	
To prevent discrimination based on race or ethnicity						
Never justified	1616	65.1 (63.0,67.3)	89	40.5 (33.0,48.1)	41	29.0 (20.3,37.7)
Sometimes justified	612	25.4 (23.5,27.4)	68	36.7 (28.9,44.4)	33	28.2 (18.7,37.6)
Usually or always justified	177	8.8 (7.4,10.2)	39	22.3 (15.5,29.1)	42	40.5 (30.3,50.6)
Adjusted prevalence difference* (95% CI; q-value)	Referent		10.0 (3.2,16.8; 0.03)		27.1 (16.8,37.4; <0.001)	
To preserve an American way of life based on Western European traditions						
Never justified	1989	82.1 (80.3,83.8)	92	44.0 (36.3,51.8)	48	39.6 (29.7,49.5)
Sometimes justified	325	12.7 (11.2,14.1)	74	38.8 (31.1,46.5)	31	25.4 (16.4,34.4)
Usually or always justified	93	4.7 (3.6,5.9)	30	15.6 (9.6,21.6)	37	32.4 (22.8,42.0)
Adjusted prevalence difference* (95% CI; q-value)	Referent		7.8 (1.6,13.9; 0.13)		25.5 (15.7,35.2; <0.001)	
To preserve the American way of life I believe in						
Never justified	1545	63.6 (61.4,65.7)	60	28.7 (21.9,35.5)	28	22.8 (14.6,31.0)
Sometimes justified	646	26.2 (24.3,28.2)	86	44.7 (36.9,52.6)	33	31.9 (22.0,41.9)
Usually or always justified	222	9.9 (8.4,11.4)	51	26.5 (19.4,33.7)	56	43.6 (33.5,53.7)
Adjusted prevalence difference* (95% CI; q-value)	Referent		11.5 (4.2,18.8; 0.01)		30.1 (20.1,40.2; <0.001)	
To oppose Americans who do not share my beliefs						
Never justified	2246	90.9 (89.4,92.4)	138	65.6 (57.9,73.4)	59	44.6 (34.6,54.7)
Sometimes justified	138	6.6 (5.4,7.8)	42	23.7 (16.7,30.7)	23	18.5 (10.9,26.1)
Usually or always justified	32	2.3 (1.4,3.2)	17	10.7 (5.4,16.0)	35	35.2 (25.0,45.5)
Adjusted prevalence difference* (95% CI; q-value)	Referent		4.9 (-0.3,10.2; 0.21)		28.1 (18.5,37.8; <0.001)	
To oppose the government when it does not share my beliefs						
Never justified	2111	85.4 (83.7,87.1)	113	54.4 (46.5,62.3)	48	35.2 (25.8,44.6)
Sometimes justified	243	11.3 (9.8,12.8)	60	31.1 (23.8,38.4)	30	27.0 (17.7,36.4)
Usually or always justified	54	2.6 (1.8,3.4)	23	14.0 (7.9,20.1)	39	36.1 (26.2,46.0)
Adjusted prevalence difference* (95% CI; q-value)	Referent		8.7 (2.8,14.6; 0.04)		30.8 (21.2,40.3; <0.001)	
To oppose the government when it tries to take private land for public purposes						
Never justified	1741	70.1 (67.9,72.2)	66	33.3 (26.0,40.7)	32	22.9 (15.0,30.9)
Sometimes justified	516	21.8 (19.9,23.7)	88	43.6 (35.8,51.4)	33	26.3 (17.6,35.0)
Usually or always justified	151	7.6 (6.3,9.0)	42	22.7 (15.9,29.5)	52	49.1 (38.8,59.3)
Adjusted prevalence difference* (95% CI; q-value)	Referent		10.8 (3.7,18.0; 0.03)		36.4 (26.0,46.8; <0.001)	

Table S7, continued.

What do you think about the use of force or violence in the following situations?	QAnon					
	Do Not Approve		Somewhat Approve		Strongly/Very Strongly Approve	
	Unweighted n	Weighted % (95% CI)	Unweighted n	Weighted % (95% CI)	Unweighted n	Weighted % (95% CI)
In general...to advance an important political objective that you support						
Never justified	3982	81.3 (80.0,82.6)	128	58.1 (50.5,65.7)	58	41.6 (31.5,51.8)
Sometimes justified	699	16.9 (15.7,18.2)	69	35.2 (27.8,42.6)	26	21.7 (12.9,30.4)
Usually or always justified	54	1.7 (1.2,2.2)	12	6.7 (2.8,10.6)	34	36.7 (25.9,47.5)
Adjusted prevalence difference* (95% CI; q-value)	Referent		2.8 (-0.9,6.5; 0.67)		32.2 (21.8,42.6; <0.001)	
Usually or always justified to advance at least 1 of 17 objectives	1181	25.4 (24.0,26.8)	118	60.2 (52.8,67.5)	80	72.1 (63.2,81.0)
Adjusted prevalence difference* (95% CI; q-value)	Referent		27.8 (20.2,35.4; <0.001)		40.4 (31.2,49.5; <0.001)	
To return Donald Trump to the presidency this year						
Never justified	4492	94.4 (93.6,95.2)	144	65.1 (57.5,72.7)	55	42.7 (32.2,53.1)
Sometimes justified	114	2.6 (2.1,3.1)	36	19.0 (12.5,25.5)	19	17.8 (9.4,26.3)
Usually or always justified	100	2.4 (1.8,3.0)	28	15.3 (9.4,21.2)	42	36.7 (26.5,47.0)
Adjusted prevalence difference* (95% CI; q-value)	Referent		10.5 (5.0,16.1; 0.003)		33.4 (23.3,43.6; <0.001)	
To stop an election from being stolen						
Never justified	3852	81.0 (79.8,82.3)	90	43.4 (35.8,50.9)	47	34.9 (25.2,44.6)
Sometimes justified	621	13.2 (12.1,14.2)	68	30.6 (23.8,37.5)	24	21.9 (12.6,31.1)
Usually or always justified	235	5.2 (4.5,6.0)	49	25.1 (18.4,31.8)	46	41.5 (31.0,52.1)
Adjusted prevalence difference* (95% CI; q-value)	Referent		16.4 (10.0,22.8; <0.001)		34.6 (23.9,45.3; <0.001)	
To stop people who do not share my beliefs from voting						
Never justified	4585	96.2 (95.5,96.8)	164	74.4 (67.6,81.3)	67	48.2 (37.6,58.8)
Sometimes justified	82	2.1 (1.6,2.6)	32	17.3 (11.5,23.1)	17	16.4 (8.2,24.5)
Usually or always justified	49	1.3 (0.9,1.8)	12	7.7 (3.1,12.3)	33	33.7 (23.2,44.2)
Adjusted prevalence difference* (95% CI; q-value)	Referent		5.0 (0.8,9.1; 0.11)		30.1 (19.9,40.3; <0.001)	
To prevent discrimination based on race or ethnicity						
Never justified	3152	64.6 (63.0,66.1)	111	49.6 (42.0,57.2)	47	34.1 (24.6,43.6)
Sometimes justified	1247	27.4 (25.9,28.8)	64	30.1 (23.2,37.0)	29	24.6 (15.0,34.2)
Usually or always justified	305	7.4 (6.5,8.3)	33	19.7 (13.1,26.2)	40	38.9 (28.3,49.4)
Adjusted prevalence difference* (95% CI; q-value)	Referent		7.8 (1.8,13.8; 0.07)		27.3 (16.8,37.9; <0.001)	
To preserve an American way of life based on Western European traditions						
Never justified	3830	81.7 (80.5,83.0)	96	45.7 (38.1,53.3)	46	35.1 (25.2,45.0)
Sometimes justified	725	14.4 (13.3,15.5)	84	37.0 (29.9,44.2)	31	24.1 (15.3,33.0)
Usually or always justified	145	3.1 (2.5,3.7)	27	15.3 (9.4,21.2)	38	37.6 (27.1,48.2)
Adjusted prevalence difference* (95% CI; q-value)	Referent		10.3 (4.7,15.8; 0.004)		34.2 (23.7,44.7; <0.001)	
To preserve the American way of life I believe in						
Never justified	2959	63.4 (61.9,64.9)	58	27.3 (20.4,34.1)	28	20.3 (12.6,28.0)
Sometimes justified	1377	28.3 (26.9,29.7)	94	43.9 (36.4,51.4)	38	34.4 (24.0,44.7)
Usually or always justified	384	8.0 (7.1,8.9)	57	28.8 (21.8,35.8)	51	43.6 (33.0,54.2)
Adjusted prevalence difference* (95% CI; q-value)	Referent		16.7 (9.7,23.8; <0.001)		33.5 (22.9,44.0; <0.001)	
To oppose Americans who do not share my beliefs						
Never justified	4427	92.4 (91.5,93.3)	160	73.6 (66.8,80.5)	63	44.2 (33.9,54.5)
Sometimes justified	248	6.0 (5.2,6.8)	34	17.0 (11.3,22.7)	19	17.0 (8.9,25.0)
Usually or always justified	50	1.4 (1.0,1.9)	15	9.3 (4.5,14.2)	35	37.2 (26.4,47.9)
Adjusted prevalence difference* (95% CI; q-value)	Referent		6.0 (1.5,10.5; 0.07)		33.5 (23.2,43.8; <0.001)	
To oppose the government when it does not share my beliefs						
Never justified	4079	84.1 (82.9,85.4)	115	54.9 (47.3,62.5)	48	34.1 (24.6,43.6)
Sometimes justified	548	13.4 (12.2,14.5)	78	36.1 (28.7,43.4)	30	23.8 (14.8,32.8)
Usually or always justified	81	1.8 (1.4,2.3)	15	8.5 (4.2,12.8)	39	40.4 (29.6,51.1)
Adjusted prevalence difference* (95% CI; q-value)	Referent		5.4 (1.2,9.7; 0.09)		36.8 (26.5,47.1; <0.001)	
To oppose the government when it tries to take private land for public purposes						
Never justified	3281	67.7 (66.2,69.2)	71	32.8 (25.7,39.9)	36	26.8 (17.9,35.7)
Sometimes justified	1158	25.1 (23.7,26.5)	101	45.8 (38.3,53.4)	29	21.8 (13.8,29.8)
Usually or always justified	263	6.5 (5.6,7.3)	36	20.7 (14.3,27.2)	52	49.6 (39.0,60.3)
Adjusted prevalence difference* (95% CI; q-value)	Referent		9.2 (2.8,15.6; 0.03)		39.1 (28.6,49.7; <0.001)	

Table S7, continued.

What do you think about the use of force or violence in the following situations?	Christian Nationalist Movement					
	Do Not Approve		Somewhat Approve		Strongly/Very Strongly Approve	
	Unweighted n	Weighted % (95% CI)	Unweighted n	Weighted % (95% CI)	Unweighted n	Weighted % (95% CI)
In general...to advance an important political objective that you support						
Never justified	2708	79.3 (77.7,81.0)	449	75.0 (70.7,79.3)	238	59.2 (53.1,65.4)
Sometimes justified	537	19.0 (17.4,20.6)	103	19.2 (15.5,22.8)	76	25.4 (19.8,31.1)
Usually or always justified	36	1.6 (1.0,2.2)	19	5.8 (2.8,8.9)	42	15.3 (10.5,20.2)
Adjusted prevalence difference* (95% CI; q-value)	Referent		3.8 (0.9,6.6; 0.05)		12.4 (7.8,17.0; <0.001)	
Usually or always justified to advance at least 1 of 17 objectives	771	24.6 (22.9,26.3)	293	50.9 (46.3,55.6)	254	70.5 (65.0,76.1)
Adjusted prevalence difference* (95% CI; q-value)	Referent		20.2 (15.1,25.2; <0.001)		38.4 (32.4,44.5; <0.001)	
To return Donald Trump to the presidency this year						
Never justified	3128	94.8 (93.9,95.7)	439	73.5 (69.2,77.9)	223	59.2 (53.2,65.3)
Sometimes justified	62	2.1 (1.5,2.6)	70	14.1 (10.5,17.7)	49	14.2 (9.8,18.6)
Usually or always justified	77	2.7 (2.0,3.3)	54	10.7 (7.7,13.6)	78	24.7 (19.3,30.2)
Adjusted prevalence difference* (95% CI; q-value)	Referent		6.9 (3.9,9.8; <0.001)		20.5 (15.2,25.9; <0.001)	
To stop an election from being stolen						
Never justified	2704	81.8 (80.3,83.3)	350	59.7 (55.1,64.3)	161	44.8 (38.9,50.8)
Sometimes justified	411	12.6 (11.3,13.8)	137	24.5 (20.4,28.5)	73	20.6 (15.6,25.5)
Usually or always justified	147	4.9 (4.0,5.8)	80	14.9 (11.4,18.4)	120	33.6 (27.9,39.2)
Adjusted prevalence difference* (95% CI; q-value)	Referent		7.9 (4.3,11.4; <0.001)		25.9 (20.2,31.7; <0.001)	
To stop people who do not share my beliefs from voting						
Never justified	3181	96.2 (95.4,97.0)	498	83.1 (79.2,87.0)	278	72.2 (66.4,78.0)
Sometimes justified	55	2.0 (1.4,2.5)	52	11.7 (8.3,15.1)	33	10.6 (6.8,14.4)
Usually or always justified	37	1.6 (1.0,2.2)	19	4.4 (2.2,6.6)	43	16.2 (11.1,21.2)
Adjusted prevalence difference* (95% CI; q-value)	Referent		2.2 (-0.1,4.4; 0.16)		13.0 (8.3,17.7; <0.001)	
To prevent discrimination based on race or ethnicity						
Never justified	2123	62.3 (60.5,64.2)	346	57.6 (52.9,62.3)	187	48.6 (42.6,54.6)
Sometimes justified	916	28.8 (27.1,30.6)	148	26.3 (22.2,30.5)	78	22.1 (17.0,27.1)
Usually or always justified	228	8.3 (7.2,9.5)	73	15.0 (11.4,18.6)	87	28.0 (22.4,33.5)
Adjusted prevalence difference* (95% CI; q-value)	Referent		3.9 (0.1,7.6; 0.15)		15.5 (9.9,21.1; <0.001)	
To preserve an American way of life based on Western European traditions						
Never justified	2777	85.2 (83.9,86.6)	311	53.4 (48.7,58.0)	163	44.2 (38.2,50.1)
Sometimes justified	398	11.5 (10.3,12.7)	200	34.0 (29.7,38.4)	108	28.2 (22.8,33.5)
Usually or always justified	88	2.7 (2.0,3.3)	53	11.1 (7.7,14.6)	79	25.3 (19.9,30.8)
Adjusted prevalence difference* (95% CI; q-value)	Referent		7.7 (4.3,11.0; <0.001)		22.2 (16.6,27.8; <0.001)	
To preserve the American way of life I believe in						
Never justified	2151	65.8 (64.0,67.7)	206	37.3 (32.7,41.9)	97	26.2 (21.1,31.4)
Sometimes justified	889	26.6 (24.9,28.3)	249	42.4 (37.9,47.0)	107	30.7 (25.0,36.3)
Usually or always justified	232	7.3 (6.3,8.3)	115	19.8 (16.0,23.6)	150	42.1 (36.2,48.0)
Adjusted prevalence difference* (95% CI; q-value)	Referent		8.9 (5.0,12.9; <0.001)		31.5 (25.4,37.6; <0.001)	
To oppose Americans who do not share my beliefs						
Never justified	3066	92.0 (90.9,93.2)	467	77.4 (73.3,81.6)	251	65.7 (59.8,71.7)
Sometimes justified	174	6.3 (5.3,7.3)	80	15.9 (12.4,19.4)	53	15.1 (10.7,19.4)
Usually or always justified	37	1.5 (1.0,2.1)	23	6.2 (3.4,8.9)	50	18.2 (13.1,23.3)
Adjusted prevalence difference* (95% CI; q-value)	Referent		3.9 (1.3,6.5; 0.02)		14.8 (9.9,19.6; <0.001)	
To oppose the government when it does not share my beliefs						
Never justified	2838	84.1 (82.6,85.6)	415	69.3 (64.7,73.8)	219	59.4 (53.4,65.4)
Sometimes justified	366	13.2 (11.8,14.6)	119	21.0 (17.1,24.9)	82	22.3 (17.3,27.3)
Usually or always justified	62	2.2 (1.6,2.7)	33	8.6 (5.3,11.8)	51	16.9 (12.1,21.6)
Adjusted prevalence difference* (95% CI; q-value)	Referent		5.9 (2.9,9.0; 0.001)		13.1 (8.5,17.7; <0.001)	
To oppose the government when it tries to take private land for public purposes						
Never justified	2326	69.1 (67.3,70.9)	261	45.3 (40.6,49.9)	120	32.4 (26.9,38.0)
Sometimes justified	752	23.8 (22.1,25.5)	210	34.8 (30.5,39.2)	120	32.0 (26.4,37.5)
Usually or always justified	181	6.4 (5.4,7.3)	95	18.8 (14.9,22.8)	114	34.6 (28.8,40.4)
Adjusted prevalence difference* (95% CI; q-value)	Referent		10.1 (6.1,14.1; <0.001)		24.2 (18.4,30.1; <0.001)	

Table S7, continued.

What do you think about the use of force or violence in the following situations?	White Supremacy Movement					
	Do Not Approve		Somewhat Approve		Strongly/Very Strongly Approve	
	Unweighted n	Weighted % (95% CI)	Unweighted n	Weighted % (95% CI)	Unweighted n	Weighted % (95% CI)
In general...to advance an important political objective that you support						
Never justified	5755	82.1 (81.0,83.1)	85	45.5 (36.7,54.2)	25	25.6 (15.0,36.1)
Sometimes justified	999	16.4 (15.4,17.4)	55	31.7 (23.6,39.8)	20	33.0 (20.1,45.8)
Usually or always justified	72	1.4 (1.1,1.8)	23	22.8 (13.9,31.7)	28	41.5 (28.4,54.6)
Adjusted prevalence difference* (95% CI; q-value)	Referent		20.5 (11.9,29.2; <0.001)		38.2 (25.3,51.1; <0.001)	
Usually or always justified to advance at least 1 of 17 objectives	1971	29.2 (28.0,30.4)	113	68.9 (60.6,77.2)	60	82.0 (71.3,92.7)
Adjusted prevalence difference* (95% CI; q-value)	Referent		33.4 (24.9,42.0; <0.001)		48.0 (37.1,59.0; <0.001)	
To return Donald Trump to the presidency this year						
Never justified	6344	92.6 (91.8,93.3)	80	46.6 (37.7,55.6)	19	24.9 (13.7,36.1)
Sometimes justified	235	3.6 (3.1,4.1)	34	20.9 (13.8,28.0)	17	23.4 (11.7,35.0)
Usually or always justified	203	3.2 (2.7,3.7)	45	29.7 (21.3,38.0)	35	47.6 (34.4,60.8)
Adjusted prevalence difference* (95% CI; q-value)	Referent		25.2 (16.7,33.7; <0.001)		44.7 (31.5,57.9; <0.001)	
To stop an election from being stolen						
Never justified	5367	78.6 (77.6,79.7)	54	32.0 (23.7,40.2)	14	17.8 (8.0,27.7)
Sometimes justified	979	14.3 (13.4,15.2)	60	35.6 (27.1,44.1)	18	27.9 (15.4,40.3)
Usually or always justified	437	6.4 (5.8,7.1)	45	29.7 (21.3,38.0)	39	50.2 (36.9,63.4)
Adjusted prevalence difference* (95% CI; q-value)	Referent		21.6 (13.1,30.0; <0.001)		44.7 (31.5,57.9; <0.001)	
To stop people who do not share my beliefs from voting						
Never justified	6585	95.9 (95.3,96.5)	102	55.5 (46.4,64.5)	33	35.4 (23.5,47.3)
Sometimes justified	144	2.4 (2.0,2.9)	37	24.8 (16.8,32.7)	12	19.5 (9.0,30.0)
Usually or always justified	71	1.3 (1.0,1.6)	20	17.0 (9.5,24.4)	26	40.9 (27.6,54.2)
Adjusted prevalence difference* (95% CI; q-value)	Referent		15.4 (8.0,22.8; <0.001)		39.5 (26.5,52.6; <0.001)	
To prevent discrimination based on race or ethnicity						
Never justified	4538	64.6 (63.3,65.9)	64	33.2 (25.2,41.2)	22	26.2 (15.0,37.4)
Sometimes justified	1769	26.9 (25.7,28.1)	59	37.4 (28.7,46.1)	17	22.9 (11.5,34.3)
Usually or always justified	478	7.9 (7.1,8.7)	35	26.4 (18.0,34.8)	32	46.8 (33.6,59.9)
Adjusted prevalence difference* (95% CI; q-value)	Referent		15.2 (6.8,23.7; 0.004)		36.6 (23.5,49.8; <0.001)	
To preserve an American way of life based on Western European traditions						
Never justified	5322	79.1 (78.0,80.1)	53	31.7 (23.4,39.9)	18	21.9 (11.5,32.4)
Sometimes justified	1175	16.2 (15.2,17.1)	73	43.0 (34.2,51.8)	21	27.8 (15.7,39.9)
Usually or always justified	265	3.9 (3.4,4.5)	33	21.2 (13.6,28.8)	32	46.1 (33.0,59.3)
Adjusted prevalence difference* (95% CI; q-value)	Referent		17.8 (9.9,25.7; <0.001)		43.7 (30.5,56.9; <0.001)	
To preserve the American way of life I believe in						
Never justified	3968	59.4 (58.1,60.7)	39	23.2 (16.1,30.3)	9	12.9 (3.9,22.0)
Sometimes justified	2138	30.3 (29.1,31.5)	72	45.3 (36.3,54.2)	19	29.8 (17.1,42.6)
Usually or always justified	700	10.0 (9.2,10.8)	51	30.2 (21.8,38.6)	43	53.1 (39.7,66.4)
Adjusted prevalence difference* (95% CI; q-value)	Referent		18.5 (10.0,27.0; <0.001)		44.1 (30.7,57.6; <0.001)	
To oppose Americans who do not share my beliefs						
Never justified	6352	92.1 (91.3,92.8)	93	52.0 (43.0,61.0)	30	36.8 (24.4,49.1)
Sometimes justified	386	6.5 (5.8,7.1)	47	28.9 (20.8,36.9)	15	20.5 (9.8,31.2)
Usually or always justified	77	1.3 (1.0,1.7)	22	17.8 (10.0,25.5)	26	38.6 (25.5,51.7)
Adjusted prevalence difference* (95% CI; q-value)	Referent		15.8 (8.3,23.3; <0.001)		37.6 (24.6,50.7; <0.001)	
To oppose the government when it does not share my beliefs						
Never justified	5806	83.6 (82.6,84.6)	82	45.2 (36.4,53.9)	21	25.0 (14.1,35.9)
Sometimes justified	863	13.8 (12.8,14.8)	51	30.4 (22.2,38.6)	18	24.6 (12.7,36.4)
Usually or always justified	123	2.1 (1.7,2.5)	26	21.7 (13.4,29.9)	31	44.6 (31.5,57.7)
Adjusted prevalence difference* (95% CI; q-value)	Referent		19.0 (10.8,27.1; <0.001)		43.2 (30.2,56.2; <0.001)	
To oppose the government when it tries to take private land for public purposes						
Never justified	4464	64.1 (62.9,65.4)	43	26.0 (18.3,33.6)	14	17.5 (7.8,27.2)
Sometimes justified	1841	27.3 (26.1,28.5)	69	38.4 (29.9,46.9)	22	28.9 (17.4,40.4)
Usually or always justified	471	7.9 (7.1,8.6)	47	32.8 (24.0,41.6)	35	49.4 (36.2,62.7)
Adjusted prevalence difference* (95% CI; q-value)	Referent		22.3 (13.3,31.3; <0.001)		39.4 (26.5,52.3; <0.001)	

Table S7, continued.

What do you think about the use of force or violence in the following situations?	Militia Movement					
	Do Not Approve		Somewhat Approve		Strongly/Very Strongly Approve	
	Unweighted n	Weighted % (95% CI)	Unweighted n	Weighted % (95% CI)	Unweighted n	Weighted % (95% CI)
In general...to advance an important political objective that you support						
Never justified	3728	84.5 (83.2,85.8)	247	58.4 (52.7,64.2)	62	39.0 (30.0,48.0)
Sometimes justified	523	14.0 (12.7,15.2)	127	34.1 (28.6,39.6)	45	28.5 (20.2,36.8)
Usually or always justified	48	1.5 (1.0,2.0)	20	7.5 (3.8,11.1)	35	32.5 (22.8,42.3)
Adjusted prevalence difference* (95% CI; q-value)	Referent		5.1 (1.5,8.6; 0.03)		28.5 (19.4,37.7; <0.001)	
Usually or always justified to advance at least 1 of 17 objectives	1125	26.9 (25.4,28.4)	232	57.4 (51.6,63.1)	108	80.4 (73.6,87.1)
Adjusted prevalence difference* (95% CI; q-value)	Referent		27.2 (21.2,33.2; <0.001)		48.0 (40.7,55.3; <0.001)	
To return Donald Trump to the presidency this year						
Never justified	4034	93.1 (92.2,94.0)	301	74.1 (68.8,79.5)	81	54.6 (45.0,64.2)
Sometimes justified	106	2.8 (2.2,3.4)	47	12.0 (8.0,15.9)	17	11.2 (4.9,17.5)
Usually or always justified	136	3.5 (2.8,4.2)	42	12.7 (8.4,17.0)	44	34.2 (25.0,43.5)
Adjusted prevalence difference* (95% CI; q-value)	Referent		7.5 (3.4,11.6; 0.004)		28.0 (18.9,37.0; <0.001)	
To stop an election from being stolen						
Never justified	3539	81.9 (80.6,83.2)	177	47.3 (41.5,53.1)	45	27.7 (19.8,35.7)
Sometimes justified	502	11.8 (10.7,12.9)	140	32.9 (27.7,38.1)	33	25.8 (17.0,34.7)
Usually or always justified	234	5.7 (4.9,6.4)	74	18.6 (14.1,23.2)	64	46.4 (36.8,56.0)
Adjusted prevalence difference* (95% CI; q-value)	Referent		12.1 (7.5,16.7; <0.001)		39.2 (29.9,48.5; <0.001)	
To stop people who do not share my beliefs from voting						
Never justified	4149	95.5 (94.7,96.3)	345	84.8 (80.3,89.3)	88	56.9 (47.3,66.5)
Sometimes justified	87	2.5 (1.9,3.1)	35	9.8 (6.3,13.3)	15	12.7 (5.8,19.7)
Usually or always justified	50	1.6 (1.1,2.2)	11	4.3 (1.4,7.2)	39	30.4 (21.3,39.4)
Adjusted prevalence difference* (95% CI; q-value)	Referent		1.9 (-1.0,4.8; 0.46)		25.9 (17.2,34.5; <0.001)	
To prevent discrimination based on race or ethnicity						
Never justified	2903	65.6 (64.0,67.2)	209	52.5 (46.7,58.3)	65	39.9 (30.7,49.1)
Sometimes justified	1069	25.7 (24.2,27.1)	130	31.7 (26.4,37.0)	31	23.2 (14.7,31.7)
Usually or always justified	300	8.1 (7.1,9.1)	52	14.8 (10.5,19.1)	46	36.8 (27.5,46.2)
Adjusted prevalence difference* (95% CI; q-value)	Referent		5.4 (0.9,9.9; 0.11)		24.1 (15.0,33.1; <0.001)	
To preserve an American way of life based on Western European traditions						
Never justified	3482	81.7 (80.4,82.9)	171	46.4 (40.7,52.2)	63	38.9 (29.9,47.9)
Sometimes justified	640	14.0 (12.9,15.2)	164	38.8 (33.3,44.3)	39	26.7 (18.2,35.2)
Usually or always justified	147	3.6 (2.9,4.2)	55	13.5 (9.6,17.3)	40	34.4 (24.8,44.0)
Adjusted prevalence difference* (95% CI; q-value)	Referent		9.5 (5.5,13.4; <0.001)		29.7 (20.3,39.0; <0.001)	
To preserve the American way of life I believe in						
Never justified	2664	62.8 (61.2,64.4)	101	28.7 (23.3,34.2)	29	16.3 (10.2,22.5)
Sometimes justified	1239	28.1 (26.6,29.6)	180	44.1 (38.4,49.8)	49	37.6 (28.2,47.0)
Usually or always justified	387	8.9 (7.9,9.8)	112	26.5 (21.5,31.5)	64	46.1 (36.5,55.7)
Adjusted prevalence difference* (95% CI; q-value)	Referent		16.4 (11.3,21.4; <0.001)		35.4 (26.0,44.9; <0.001)	
To oppose Americans who do not share my beliefs						
Never justified	4029	92.3 (91.3,93.3)	313	77.9 (72.9,82.8)	83	52.2 (42.6,61.8)
Sometimes justified	213	5.9 (5.1,6.8)	64	15.8 (11.7,20.0)	22	16.9 (9.7,24.1)
Usually or always justified	51	1.6 (1.1,2.1)	16	5.7 (2.5,8.8)	37	30.9 (21.6,40.1)
Adjusted prevalence difference* (95% CI; q-value)	Referent		3.5 (0.4,6.6; 0.15)		26.8 (18.0,35.6; <0.001)	
To oppose the government when it does not share my beliefs						
Never justified	3811	87.0 (85.8,88.2)	221	55.7 (49.9,61.4)	56	37.3 (28.3,46.4)
Sometimes justified	392	10.5 (9.4,11.6)	150	37.3 (31.8,42.9)	42	26.7 (18.4,35.1)
Usually or always justified	78	2.0 (1.5,2.5)	20	6.0 (3.1,8.9)	42	34.5 (25.0,43.9)
Adjusted prevalence difference* (95% CI; q-value)	Referent		3.3 (0.3,6.2; 0.21)		30.4 (21.5,39.4; <0.001)	
To oppose the government when it tries to take private land for public purposes						
Never justified	3069	70.4 (68.8,71.9)	116	31.0 (25.6,36.5)	34	22.4 (14.9,29.8)
Sometimes justified	970	22.6 (21.2,24.0)	194	47.0 (41.2,52.7)	41	24.9 (17.2,32.7)
Usually or always justified	236	6.4 (5.5,7.3)	80	20.7 (16.1,25.2)	67	52.7 (43.2,62.1)
Adjusted prevalence difference* (95% CI; q-value)	Referent		12.0 (7.2,16.8; <0.001)		41.2 (31.9,50.6; <0.001)	

Table S7, continued.

What do you think about the use of force or violence in the following situations?	Boogaloo Movement					
	Do Not Approve		Somewhat Approve		Strongly/Very Strongly Approve	
	Unweighted n	Weighted % (95% CI)	Unweighted n	Weighted % (95% CI)	Unweighted n	Weighted % (95% CI)
In general...to advance an important political objective that you support						
Never justified	2191	80.7 (79.0,82.5)	43	43.5 (32.1,55.0)	13	20.2 (8.6,31.8)
Sometimes justified	396	16.8 (15.1,18.4)	38	33.4 (23.2,43.5)	16	29.2 (15.9,42.5)
Usually or always justified	46	2.4 (1.6,3.2)	16	23.1 (12.1,34.1)	28	50.6 (36.0,65.1)
Adjusted prevalence difference* (95% CI; q-value)	Referent		17.5 (7.4,27.5; 0.01)		44.4 (30.1,58.7; <0.001)	
Usually or always justified to advance at least 1 of 17 objectives	690	27.3 (25.3,29.2)	46	51.6 (40.0,63.1)	51	90.5 (81.9,99.1)
Adjusted prevalence difference* (95% CI; q-value)	Referent		16.7 (4.8,28.6; 0.02)		53.6 (43.0,64.2; <0.001)	
To return Donald Trump to the presidency this year						
Never justified	2461	92.5 (91.2,93.7)	63	59.2 (47.6,70.9)	13	26.7 (13.0,40.5)
Sometimes justified	71	3.1 (2.3,3.9)	20	22.5 (12.3,32.7)	9	12.1 (3.8,20.3)
Usually or always justified	88	3.8 (2.9,4.8)	11	14.6 (5.8,23.5)	35	61.2 (46.8,75.5)
Adjusted prevalence difference* (95% CI; q-value)	Referent		8.5 (-0.7,17.7; 0.29)		53.6 (39.7,67.4; <0.001)	
To stop an election from being stolen						
Never justified	2113	79.2 (77.4,81.0)	42	44.1 (32.6,55.7)	6	12.1 (1.6,22.6)
Sometimes justified	343	13.1 (11.6,14.5)	35	32.4 (22.1,42.7)	18	33.7 (19.6,47.9)
Usually or always justified	164	7.1 (5.9,8.3)	17	19.8 (10.0,29.7)	33	54.2 (39.5,68.9)
Adjusted prevalence difference* (95% CI; q-value)	Referent		10.2 (0.3,20.2; 0.21)		43.0 (28.4,57.7; <0.001)	
To stop people who do not share my beliefs from voting						
Never justified	2519	94.6 (93.5,95.6)	63	62.8 (51.6,74.0)	14	21.3 (9.7,32.9)
Sometimes justified	60	2.7 (2.0,3.5)	23	22.5 (13.1,31.9)	11	21.1 (8.7,33.6)
Usually or always justified	48	2.4 (1.6,3.2)	8	11.0 (3.1,18.9)	32	57.6 (43.1,72.0)
Adjusted prevalence difference* (95% CI; q-value)	Referent		6.3 (-1.7,14.4; 0.32)		49.9 (36.2,63.6; <0.001)	
To prevent discrimination based on race or ethnicity						
Never justified	1718	63.8 (61.7,65.9)	40	37.1 (26.0,48.2)	14	22.4 (10.4,34.4)
Sometimes justified	694	26.3 (24.4,28.2)	40	39.7 (28.6,50.7)	12	24.3 (11.1,37.6)
Usually or always justified	208	9.3 (8.0,10.6)	14	19.6 (9.4,29.8)	30	52.0 (37.4,66.6)
Adjusted prevalence difference* (95% CI; q-value)	Referent		5.1 (-5.0,15.2; 0.63)		35.4 (20.7,50.1; <0.001)	
To preserve an American way of life based on Western European traditions						
Never justified	2136	81.3 (79.6,83.0)	46	50.5 (38.9,62.0)	8	12.8 (3.0,22.6)
Sometimes justified	389	14.0 (12.6,15.5)	34	31.3 (21.1,41.6)	20	33.3 (19.9,46.8)
Usually or always justified	97	4.2 (3.2,5.1)	14	14.5 (6.1,23.0)	27	48.6 (34.0,63.1)
Adjusted prevalence difference* (95% CI; q-value)	Referent		8.5 (0.1,16.9; 0.41)		44.0 (29.3,58.7; <0.001)	
To preserve the American way of life I believe in						
Never justified	1639	62.2 (60.1,64.2)	35	37.3 (26.1,48.4)	9	13.1 (3.6,22.5)
Sometimes justified	735	27.3 (25.4,29.1)	40	39.7 (28.3,51.0)	15	30.9 (17.2,44.7)
Usually or always justified	253	10.3 (8.9,11.7)	21	21.1 (11.6,30.5)	33	56.0 (41.5,70.5)
Adjusted prevalence difference* (95% CI; q-value)	Referent		6.9 (-2.4,16.3; 0.39)		40.9 (26.2,55.6; <0.001)	
To oppose Americans who do not share my beliefs						
Never justified	2417	90.1 (88.7,91.5)	57	56.8 (45.2,68.4)	16	26.8 (13.6,40.1)
Sometimes justified	170	7.5 (6.3,8.7)	27	25.3 (15.5,35.2)	12	22.9 (10.6,35.3)
Usually or always justified	43	2.2 (1.5,3.0)	12	15.8 (6.5,25.2)	29	50.3 (35.7,64.8)
Adjusted prevalence difference* (95% CI; q-value)	Referent		11.3 (2.7,20.0; 0.06)		44.0 (30.5,57.5; <0.001)	
To oppose the government when it does not share my beliefs						
Never justified	2275	84.6 (83.0,86.2)	45	43.5 (32.2,54.8)	11	19.8 (7.6,32.0)
Sometimes justified	284	12.0 (10.5,13.4)	40	40.4 (29.0,51.9)	10	15.7 (5.9,25.6)
Usually or always justified	65	3.0 (2.2,3.8)	9	12.4 (3.9,20.9)	34	61.0 (46.9,75.2)
Adjusted prevalence difference* (95% CI; q-value)	Referent		6.4 (-1.5,14.3; 0.40)		54.7 (40.6,68.8; <0.001)	
To oppose the government when it tries to take private land for public purposes						
Never justified	1882	69.7 (67.7,71.7)	32	34.5 (23.3,45.7)	9	15.7 (5.2,26.2)
Sometimes justified	564	22.0 (20.2,23.8)	42	36.1 (25.5,46.7)	14	24.7 (12.0,37.4)
Usually or always justified	176	7.8 (6.5,9.0)	20	25.7 (15.0,36.5)	34	59.6 (45.3,74.0)
Adjusted prevalence difference* (95% CI; q-value)	Referent		12.7 (1.9,23.4; 0.09)		43.3 (28.6,58.0; <0.001)	

*	Prevalence differences are adjusted for age, race and ethnicity, gender, education, income, Census division, and rurality and are for the usually or always justified comparison. They are expressed in absolute percentage points. Q-values represent the probability that the given difference would be a false discovery; they represent the expected proportion of “false positives” that would be seen among the collection of all differences whose q-values were at or below the given q-value.

Table S8. Approval of individual organizations and movements and justification for political violence to advance 8 additional specific objectives

What do you think about the use of force or violence in the following situations?	Proud Boys					
	Do Not Approve		Somewhat Approve		Strongly/Very Strongly Approve	
	Unweighted n	Weighted % (95% CI)	Unweighted n	Weighted % (95% CI)	Unweighted n	Weighted % (95% CI)
To stop voter fraud						
Never justified	2034	82.5 (80.8,84.2)	86	47.2 (38.7,55.7)	28	31.6 (19.7,43.5)
Sometimes justified	279	11.5 (10.1,12.9)	46	28.3 (20.7,36.0)	17	21.6 (11.5,31.7)
Usually or always justified	143	5.6 (4.6,6.6)	40	24.1 (16.5,31.8)	35	46.8 (33.8,59.8)
Adjusted prevalence difference* (95% CI; q-value)	Referent		15.7 (8.4,23.0; <0.001)		37.7 (25.6,49.7; <0.001)	
To stop voter intimidation						
Never justified	1507	63.0 (60.8,65.1)	73	45.9 (36.7,55.0)	32	37.2 (25.5,48.8)
Sometimes justified	683	28.0 (26.0,30.0)	56	37.2 (28.1,46.3)	24	32.4 (20.6,44.3)
Usually or always justified	220	8.9 (7.6,10.1)	22	16.9 (9.2,24.7)	24	27.6 (16.8,38.4)
Adjusted prevalence difference* (95% CI; q-value)	Referent		5.9 (-1.9,13.7; 0.57)		18.4 (7.5,29.2; 0.03)	
To reinforce the police						
Never justified	1195	51.3 (49.1,53.5)	27	19.1 (11.9,26.2)	13	18.0 (8.1,28.0)
Sometimes justified	944	37.8 (35.7,40.0)	67	38.2 (29.5,46.8)	26	38.7 (24.9,52.5)
Usually or always justified	292	10.6 (9.3,11.9)	66	42.8 (33.6,51.9)	31	43.3 (29.6,56.9)
Adjusted prevalence difference* (95% CI; q-value)	Referent		29.1 (20.4,37.9; <0.001)		30.5 (16.5,44.5; <0.001)	
To stop police violence						
Never justified	1191	46.9 (44.7,49.1)	72	41.3 (33.0,49.6)	33	32.4 (22.0,42.8)
Sometimes justified	1005	41.9 (39.7,44.1)	66	40.8 (32.2,49.5)	36	40.5 (29.0,52.0)
Usually or always justified	239	10.9 (9.5,12.4)	26	17.9 (11.0,24.8)	21	24.8 (14.0,35.5)
Adjusted prevalence difference* (95% CI; q-value)	Referent		4.2 (-2.5,10.9; 0.37)		9.4 (-0.9,19.6; 0.27)	
To stop illegal immigration						
Never justified	1796	74.7 (72.8,76.6)	50	29.2 (21.6,36.9)	23	27.8 (17.1,38.6)
Sometimes justified	459	18.3 (16.6,20.0)	83	46.2 (37.6,54.8)	22	31.6 (19.4,43.7)
Usually or always justified	172	7.0 (5.8,8.1)	42	24.6 (16.7,32.5)	33	37.9 (26.1,49.7)
Adjusted prevalence difference* (95% CI; q-value)	Referent		15.3 (7.6,23.0; 0.001)		29.1 (17.4,40.7; <0.001)	
To keep borders open						
Never justified	1697	68.8 (66.7,70.8)	86	50.4 (41.3,59.5)	53	54.7 (41.5,67.8)
Sometimes justified	604	24.8 (22.9,26.8)	38	28.8 (20.2,37.4)	9	15.6 (4.8,26.4)
Usually or always justified	140	6.0 (4.9,7.1)	24	20.4 (12.3,28.4)	20	29.8 (17.1,42.4)
Adjusted prevalence difference* (95% CI; q-value)	Referent		13.4 (5.4,21.3; 0.01)		20.6 (8.6,32.5; 0.01)	
To stop a protest						
Never justified	1586	68.4 (66.3,70.4)	60	35.6 (27.7,43.6)	16	20.4 (10.4,30.5)
Sometimes justified	699	28.2 (26.3,30.2)	93	51.2 (42.8,59.7)	35	43.9 (30.8,56.9)
Usually or always justified	73	3.0 (2.2,3.7)	19	13.1 (7.0,19.3)	21	32.8 (19.3,46.2)
Adjusted prevalence difference* (95% CI; q-value)	Referent		7.6 (1.4,13.7; 0.19)		27.5 (14.9,40.0; <0.001)	
To support a protest						
Never justified	2080	81.0 (79.2,82.8)	94	54.5 (45.0,64.0)	47	45.4 (33.7,57.1)
Sometimes justified	362	15.9 (14.3,17.6)	41	32.8 (23.4,42.2)	19	22.6 (12.7,32.4)
Usually or always justified	66	2.9 (2.2,3.7)	17	12.7 (6.5,18.9)	22	32.1 (19.9,44.2)
Adjusted prevalence difference* (95% CI; q-value)	Referent		8.4 (2.2,14.6; 0.11)		25.8 (14.3,37.3; <0.001)	

Table S8, continued.

What do you think about the use of force or violence in the following situations?	Oath Keepers					
	Do Not Approve		Somewhat Approve		Strongly/Very Strongly Approve	
	Unweighted n	Weighted % (95% CI)	Unweighted n	Weighted % (95% CI)	Unweighted n	Weighted % (95% CI)
To stop voter fraud						
Never justified	1442	83.8 (81.8,85.8)	93	53.8 (45.0,62.6)	50	38.8 (29.1,48.5)
Sometimes justified	170	10.1 (8.5,11.7)	47	29.1 (21.2,37.1)	25	19.7 (11.9,27.5)
Usually or always justified	92	5.6 (4.3,6.8)	26	17.1 (9.7,24.5)	49	41.1 (31.3,50.9)
Adjusted prevalence difference* (95% CI; q-value)	Referent		5.7 (-0.6,12.0; 0.24)		30.2 (20.9,39.6; <0.001)	
To stop voter intimidation						
Never justified	1075	62.6 (60.0,65.1)	67	43.3 (34.3,52.2)	35	36.1 (24.9,47.3)
Sometimes justified	509	29.5 (27.1,31.8)	58	36.7 (28.0,45.4)	24	24.6 (14.4,34.8)
Usually or always justified	145	7.8 (6.5,9.2)	30	20.1 (12.6,27.5)	33	37.0 (25.5,48.6)
Adjusted prevalence difference* (95% CI; q-value)	Referent		10.2 (3.1,17.3; 0.07)		30.6 (19.2,42.1; <0.001)	
To reinforce the police						
Never justified	889	53.1 (50.5,55.7)	27	19.4 (12.0,26.8)	15	13.8 (6.8,20.9)
Sometimes justified	647	36.4 (33.9,38.9)	80	49.5 (40.7,58.4)	38	37.1 (26.1,48.1)
Usually or always justified	183	10.2 (8.6,11.7)	56	31.1 (23.3,38.9)	52	48.6 (37.5,59.7)
Adjusted prevalence difference* (95% CI; q-value)	Referent		17.3 (9.8,24.8; <0.001)		38.2 (26.9,49.5; <0.001)	
To stop police violence						
Never justified	840	47.1 (44.5,49.7)	75	44.1 (35.3,53.0)	42	35.7 (25.8,45.5)
Sometimes justified	707	41.4 (38.8,44.0)	59	37.6 (28.9,46.3)	44	40.6 (30.2,51.0)
Usually or always justified	168	11.2 (9.4,13.0)	24	18.3 (10.7,25.8)	25	21.8 (13.4,30.2)
Adjusted prevalence difference* (95% CI; q-value)	Referent		2.4 (-4.7,9.5; 0.67)		5.3 (-3.6,14.2; 0.48)	
To stop illegal immigration						
Never justified	1329	76.9 (74.7,79.2)	52	35.8 (27.3,44.2)	24	20.3 (12.1,28.4)
Sometimes justified	304	17.4 (15.4,19.5)	64	35.9 (27.9,43.9)	28	32.5 (21.7,43.3)
Usually or always justified	94	5.5 (4.2,6.7)	42	28.3 (19.9,36.7)	54	45.2 (34.7,55.8)
Adjusted prevalence difference* (95% CI; q-value)	Referent		19.8 (11.6,28.0; <0.001)		38.3 (27.7,48.9; <0.001)	
To keep borders open						
Never justified	1218	71.0 (68.6,73.4)	95	54.2 (45.2,63.3)	67	55.1 (44.2,66.1)
Sometimes justified	400	22.6 (20.4,24.8)	41	25.9 (18.0,33.8)	22	24.3 (14.0,34.6)
Usually or always justified	90	5.9 (4.5,7.3)	27	19.9 (12.2,27.6)	20	19.6 (10.8,28.4)
Adjusted prevalence difference* (95% CI; q-value)	Referent		12.3 (4.8,19.7; 0.04)		12.2 (3.0,21.3; 0.09)	
To stop a protest						
Never justified	1168	69.5 (67.0,72.0)	58	35.9 (27.5,44.2)	35	33.1 (23.0,43.2)
Sometimes justified	460	27.2 (24.8,29.6)	93	47.6 (39.1,56.1)	46	38.3 (27.9,48.8)
Usually or always justified	46	2.8 (1.8,3.7)	23	16.5 (9.3,23.8)	23	26.4 (16.2,36.7)
Adjusted prevalence difference* (95% CI; q-value)	Referent		8.1 (2.0,14.3; 0.09)		17.6 (8.5,26.8; 0.005)	
To support a protest						
Never justified	1485	82.2 (80.2,84.3)	104	66.3 (57.1,75.5)	58	44.1 (33.8,54.4)
Sometimes justified	240	15.2 (13.2,17.2)	28	22.8 (14.2,31.4)	21	23.0 (13.5,32.5)
Usually or always justified	34	2.4 (1.5,3.2)	15	10.9 (5.1,16.7)	33	32.4 (22.1,42.8)
Adjusted prevalence difference* (95% CI; q-value)	Referent		6.8 (1.1,12.6; 0.11)		26.9 (17.2,36.5; <0.001)	

Table S8, continued.

What do you think about the use of force or violence in the following situations?	Three Percenters					
	Do Not Approve		Somewhat Approve		Strongly/Very Strongly Approve	
	Unweighted n	Weighted % (95% CI)	Unweighted n	Weighted % (95% CI)	Unweighted n	Weighted % (95% CI)
To stop voter fraud						
Never justified	998	79.8 (77.0,82.5)	55	48.3 (37.6,58.9)	23	35.1 (21.5,48.8)
Sometimes justified	130	11.1 (9.1,13.2)	32	34.9 (24.4,45.3)	14	23.4 (11.6,35.2)
Usually or always justified	94	8.7 (6.7,10.8)	17	16.9 (8.6,25.1)	25	41.5 (27.5,55.4)
Adjusted prevalence difference* (95% CI; q-value)	Referent		2.6 (-6.4,11.6; 0.79)		25.2 (12.0,38.5; 0.006)	
To stop voter intimidation						
Never justified	730	61.8 (58.7,64.9)	44	44.1 (32.7,55.4)	21	32.1 (18.8,45.3)
Sometimes justified	340	27.8 (25.0,30.6)	29	35.1 (23.4,46.9)	20	36.4 (22.4,50.4)
Usually or always justified	123	10.4 (8.4,12.3)	20	20.8 (11.6,30.0)	14	28.1 (14.1,42.1)
Adjusted prevalence difference* (95% CI; q-value)	Referent		10.1 (0.7,19.6; 0.52)		19.0 (4.8,33.2; 0.26)	
To reinforce the police						
Never justified	595	49.5 (46.4,52.7)	21	25.7 (15.1,36.3)	10	17.7 (6.8,28.6)
Sometimes justified	469	37.8 (34.7,40.8)	42	46.1 (34.4,57.8)	16	27.8 (13.8,41.7)
Usually or always justified	153	12.4 (10.3,14.6)	29	28.2 (18.4,38.0)	30	54.6 (39.5,69.6)
Adjusted prevalence difference* (95% CI; q-value)	Referent		14.2 (4.5,23.9; 0.04)		41.4 (26.6,56.3; <0.001)	
To stop police violence						
Never justified	606	48.8 (45.6,52.0)	33	28.0 (19.0,37.0)	21	29.7 (17.5,41.9)
Sometimes justified	456	38.1 (35.0,41.2)	53	52.5 (41.9,63.1)	24	39.1 (25.4,52.8)
Usually or always justified	136	12.9 (10.6,15.3)	19	19.5 (11.0,28.0)	16	28.0 (15.6,40.4)
Adjusted prevalence difference* (95% CI; q-value)	Referent		1.5 (-7.8,10.9; 0.77)		9.0 (-3.3,21.3; 0.44)	
To stop illegal immigration						
Never justified	883	72.3 (69.3,75.3)	31	32.0 (21.6,42.3)	18	30.8 (17.6,44.0)
Sometimes justified	226	18.9 (16.3,21.5)	40	38.3 (27.6,49.0)	13	29.7 (14.9,44.5)
Usually or always justified	96	8.8 (6.8,10.8)	22	29.7 (18.3,41.1)	24	36.0 (22.5,49.5)
Adjusted prevalence difference* (95% CI; q-value)	Referent		15.6 (5.1,26.0; 0.03)		24.1 (10.1,38.1; 0.02)	
To keep borders open						
Never justified	841	69.0 (66.0,71.9)	46	39.9 (29.5,50.3)	28	40.4 (26.8,53.9)
Sometimes justified	286	22.8 (20.2,25.3)	35	38.4 (27.3,49.4)	17	31.4 (17.6,45.1)
Usually or always justified	83	7.8 (5.9,9.7)	23	21.8 (13.0,30.6)	17	28.3 (15.5,41.0)
Adjusted prevalence difference* (95% CI; q-value)	Referent		11.5 (1.8,21.2; 0.14)		17.6 (4.9,30.3; 0.10)	
To stop a protest						
Never justified	803	67.5 (64.5,70.6)	44	41.7 (31.2,52.3)	19	33.8 (19.9,47.8)
Sometimes justified	336	27.8 (25.0,30.7)	40	38.9 (28.3,49.4)	23	35.6 (21.6,49.7)
Usually or always justified	47	4.2 (2.8,5.7)	18	19.4 (10.4,28.4)	12	27.1 (12.8,41.3)
Adjusted prevalence difference* (95% CI; q-value)	Referent		9.0 (0.1,17.9; 0.29)		17.4 (4.3,30.5; 0.27)	
To support a protest						
Never justified	999	79.1 (76.4,81.7)	57	52.4 (40.8,64.1)	24	33.4 (20.9,46.0)
Sometimes justified	189	17.2 (14.7,19.6)	26	32.2 (20.5,44.0)	15	26.2 (13.9,38.5)
Usually or always justified	40	3.7 (2.4,5.0)	12	15.3 (6.7,24.0)	24	40.4 (26.4,54.3)
Adjusted prevalence difference* (95% CI; q-value)	Referent		9.4 (0.9,17.8; 0.38)		31.4 (18.1,44.7; <0.001)	

Table S8, continued.

What do you think about the use of force or violence in the following situations?	QAnon					
	Do Not Approve		Somewhat Approve		Strongly/Very Strongly Approve	
	Unweighted n	Weighted % (95% CI)	Unweighted n	Weighted % (95% CI)	Unweighted n	Weighted % (95% CI)
To stop voter fraud						
Never justified	1963	82.6 (80.9,84.4)	57	48.1 (38.0,58.3)	22	32.0 (18.2,45.9)
Sometimes justified	262	11.4 (10.0,12.9)	30	22.6 (14.5,30.7)	17	30.1 (16.3,43.8)
Usually or always justified	129	5.4 (4.4,6.5)	34	29.3 (20.2,38.5)	21	37.9 (22.9,52.8)
Adjusted prevalence difference* (95% CI; q-value)	Referent		19.5 (10.7,28.3; <0.001)		28.4 (14.3,42.5; 0.001)	
To stop voter intimidation						
Never justified	1450	61.6 (59.4,63.7)	44	48.8 (37.2,60.3)	21	30.5 (17.8,43.2)
Sometimes justified	690	29.1 (27.0,31.1)	28	31.0 (20.4,41.6)	20	36.5 (21.8,51.1)
Usually or always justified	227	9.2 (7.9,10.5)	16	20.3 (10.2,30.3)	16	29.6 (15.6,43.7)
Adjusted prevalence difference* (95% CI; q-value)	Referent		8.9 (-0.4,18.1; 0.31)		21.5 (6.5,36.6; 0.15)	
To reinforce the police						
Never justified	1154	51.0 (48.7,53.3)	22	23.0 (13.2,32.9)	11	15.8 (6.3,25.3)
Sometimes justified	910	37.3 (35.1,39.5)	35	36.0 (25.2,46.8)	23	39.4 (23.4,55.4)
Usually or always justified	284	11.4 (10.0,12.9)	37	40.9 (29.6,52.3)	24	44.8 (29.1,60.6)
Adjusted prevalence difference* (95% CI; q-value)	Referent		23.8 (12.7,34.8; <0.001)		32.8 (17.2,48.4; <0.001)	
To stop police violence						
Never justified	1158	46.5 (44.3,48.7)	49	41.1 (31.3,51.0)	20	27.2 (15.4,38.9)
Sometimes justified	980	42.1 (39.9,44.3)	49	44.9 (34.6,55.1)	21	34.2 (20.6,47.8)
Usually or always justified	235	11.1 (9.6,12.6)	17	14.0 (7.3,20.7)	18	35.6 (21.4,49.7)
Adjusted prevalence difference* (95% CI; q-value)	Referent		-0.6 (-7.8,6.6; 0.96)		18.8 (5.8,31.8; 0.06)	
To stop illegal immigration						
Never justified	1697	72.2 (70.2,74.2)	36	31.2 (21.8,40.7)	18	25.7 (13.7,37.7)
Sometimes justified	508	20.7 (18.9,22.5)	41	33.6 (23.6,43.7)	12	27.3 (12.6,42.1)
Usually or always justified	172	7.0 (5.8,8.2)	33	35.1 (24.7,45.6)	24	43.2 (27.9,58.6)
Adjusted prevalence difference* (95% CI; q-value)	Referent		24.5 (14.4,34.5; <0.001)		35.3 (19.5,51.2; <0.001)	
To keep borders open						
Never justified	1674	70.7 (68.6,72.8)	54	50.1 (39.2,61.0)	28	36.1 (23.0,49.2)
Sometimes justified	545	23.1 (21.2,25.0)	25	24.9 (15.6,34.2)	17	31.9 (17.5,46.3)
Usually or always justified	127	5.7 (4.6,6.9)	19	24.4 (14.6,34.3)	18	32.0 (17.9,46.1)
Adjusted prevalence difference* (95% CI; q-value)	Referent		19.2 (9.4,28.9; 0.003)		23.3 (9.8,36.8; 0.01)	
To stop a protest						
Never justified	1514	66.9 (64.7,69.0)	47	36.3 (26.8,45.7)	24	35.6 (21.9,49.4)
Sometimes justified	700	29.7 (27.6,31.7)	60	47.1 (37.2,57.0)	19	33.4 (18.8,48.0)
Usually or always justified	72	3.0 (2.2,3.8)	20	16.6 (9.1,24.0)	12	27.5 (12.4,42.5)
Adjusted prevalence difference* (95% CI; q-value)	Referent		10.2 (3.4,17.0; 0.05)		22.2 (7.6,36.7; 0.05)	
To support a protest						
Never justified	2012	80.7 (78.9,82.5)	51	55.3 (43.3,67.3)	27	35.6 (22.5,48.7)
Sometimes justified	357	16.1 (14.4,17.8)	22	30.9 (19.5,42.3)	14	24.5 (11.1,37.9)
Usually or always justified	67	3.1 (2.3,3.9)	9	13.8 (4.7,22.8)	21	39.9 (25.2,54.6)
Adjusted prevalence difference* (95% CI; q-value)	Referent		8.8 (0.4,17.3; 0.19)		32.4 (18.3,46.5; <0.001)	

Table S8, continued.

What do you think about the use of force or violence in the following situations?	Christian Nationalist Movement					
	Do Not Approve		Somewhat Approve		Strongly/Very Strongly Approve	
	Unweighted n	Weighted % (95% CI)	Unweighted n	Weighted % (95% CI)	Unweighted n	Weighted % (95% CI)
To stop voter fraud						
Never justified	1377	83.3 (81.2,85.4)	159	55.3 (48.8,61.8)	76	43.3 (34.6,51.9)
Sometimes justified	171	11.1 (9.4,12.9)	65	23.4 (17.9,29.0)	38	21.4 (14.6,28.2)
Usually or always justified	83	5.0 (3.8,6.2)	63	21.3 (15.9,26.7)	56	35.1 (26.6,43.6)
Adjusted prevalence difference* (95% CI; q-value)	Referent		12.7 (7.4,17.9; <0.001)		23.8 (15.7,31.9; <0.001)	
To stop voter intimidation						
Never justified	994	60.7 (58.1,63.3)	165	56.6 (49.9,63.3)	81	43.3 (35.1,51.5)
Sometimes justified	490	30.2 (27.7,32.7)	82	30.6 (24.2,36.9)	44	24.2 (17.0,31.5)
Usually or always justified	158	9.1 (7.6,10.5)	36	11.8 (7.7,16.0)	59	30.9 (23.3,38.4)
Adjusted prevalence difference* (95% CI; q-value)	Referent		0.6 (-3.8,5.0; 0.91)		21.9 (14.0,29.8; <0.001)	
To reinforce the police						
Never justified	902	56.7 (54.0,59.4)	65	24.1 (18.4,29.8)	30	20.6 (13.4,27.9)
Sometimes justified	570	33.7 (31.1,36.3)	133	48.6 (42.0,55.2)	56	30.9 (22.9,38.9)
Usually or always justified	152	9.3 (7.7,10.9)	83	27.3 (21.6,33.0)	91	48.2 (39.6,56.8)
Adjusted prevalence difference* (95% CI; q-value)	Referent		14.8 (9.0,20.6; <0.001)		36.5 (27.5,45.4; <0.001)	
To stop police violence						
Never justified	784	44.8 (42.2,47.5)	121	38.7 (32.4,45.0)	63	34.5 (26.6,42.5)
Sometimes justified	687	42.4 (39.8,45.1)	113	40.5 (34.0,47.0)	72	39.2 (31.0,47.5)
Usually or always justified	180	12.6 (10.7,14.5)	55	19.9 (14.4,25.3)	42	24.5 (17.4,31.7)
Adjusted prevalence difference* (95% CI; q-value)	Referent		3.8 (-1.8,9.4; 0.42)		5.7 (-1.9,13.3; 0.42)	
To stop illegal immigration						
Never justified	1272	78.5 (76.2,80.7)	103	36.1 (29.9,42.3)	47	24.1 (17.4,30.9)
Sometimes justified	258	15.7 (13.7,17.6)	116	40.3 (33.9,46.8)	70	36.9 (29.0,44.9)
Usually or always justified	92	5.8 (4.4,7.1)	66	22.6 (17.1,28.2)	73	37.3 (29.5,45.1)
Adjusted prevalence difference* (95% CI; q-value)	Referent		14.8 (9.0,20.5; <0.001)		28.8 (20.9,36.7; <0.001)	
To keep borders open						
Never justified	1186	71.2 (68.8,73.7)	147	49.2 (42.5,55.8)	88	49.4 (40.5,58.4)
Sometimes justified	393	23.7 (21.3,26.0)	88	30.3 (24.3,36.3)	36	25.3 (17.1,33.5)
Usually or always justified	74	4.7 (3.5,5.8)	50	20.5 (14.7,26.3)	39	24.7 (16.8,32.7)
Adjusted prevalence difference* (95% CI; q-value)	Referent		14.1 (8.3,19.9; <0.001)		18.2 (10.3,26.0; <0.001)	
To stop a protest						
Never justified	1130	71.6 (69.1,74.0)	114	41.8 (35.1,48.5)	67	37.7 (29.3,46.1)
Sometimes justified	422	25.4 (23.1,27.8)	121	44.2 (37.4,50.9)	64	34.5 (26.3,42.6)
Usually or always justified	42	2.5 (1.7,3.3)	33	13.1 (8.2,17.9)	37	26.0 (17.6,34.4)
Adjusted prevalence difference* (95% CI; q-value)	Referent		8.2 (3.7,12.7; 0.005)		19.4 (11.8,27.0; <0.001)	
To support a protest						
Never justified	1373	79.5 (77.3,81.7)	220	68.5 (62.2,74.8)	112	54.7 (46.5,63.0)
Sometimes justified	267	17.6 (15.5,19.7)	60	22.8 (16.9,28.6)	39	22.4 (15.4,29.3)
Usually or always justified	39	2.8 (1.9,3.8)	22	8.7 (4.8,12.6)	35	22.6 (15.3,30.0)
Adjusted prevalence difference* (95% CI; q-value)	Referent		4.5 (0.6,8.4; 0.14)		17.5 (10.4,24.5; <0.001)	

Table S8, continued.

What do you think about the use of force or violence in the following situations?	White Supremacy Movement					
	Do Not Approve		Somewhat Approve		Strongly/Very Strongly Approve	
	Unweighted n	Weighted % (95% CI)	Unweighted n	Weighted % (95% CI)	Unweighted n	Weighted % (95% CI)
To stop voter fraud						
Never justified	2714	79.1 (77.5,80.6)	20	24.0 (13.4,34.7)	9	20.3 (4.6,35.9)
Sometimes justified	456	13.6 (12.3,14.9)	26	37.6 (24.7,50.4)	11	28.2 (13.1,43.4)
Usually or always justified	251	7.0 (6.0,8.0)	30	37.6 (24.9,50.3)	23	50.5 (33.5,67.5)
Adjusted prevalence difference* (95% CI; q-value)	Referent		28.1 (15.6,40.7; <0.001)		41.7 (25.9,57.5; <0.001)	
To stop voter intimidation						
Never justified	2116	63.2 (61.4,65.0)	32	34.6 (23.5,45.8)	10	28.7 (10.4,47.0)
Sometimes justified	954	27.9 (26.2,29.6)	30	34.1 (22.2,46.0)	10	38.5 (18.1,58.9)
Usually or always justified	320	8.7 (7.7,9.7)	23	28.7 (17.4,40.0)	8	24.7 (7.8,41.6)
Adjusted prevalence difference* (95% CI; q-value)	Referent		21.1 (9.2,33.1; 0.008)		20.0 (2.4,37.5; 0.25)	
To reinforce the police						
Never justified	1462	45.3 (43.4,47.2)	8	12.4 (3.5,21.3)	3	10.1 (-1.9,22.1)
Sometimes justified	1348	38.9 (37.1,40.8)	30	37.3 (24.5,50.0)	12	39.1 (17.7,60.6)
Usually or always justified	569	15.6 (14.2,16.9)	39	50.3 (37.3,63.3)	14	49.2 (28.0,70.5)
Adjusted prevalence difference* (95% CI; q-value)	Referent		33.4 (20.9,46.0; <0.001)		37.2 (15.3,59.1; 0.004)	
To stop police violence						
Never justified	1673	46.9 (45.0,48.8)	20	18.9 (10.3,27.6)	15	32.0 (16.5,47.4)
Sometimes justified	1410	42.0 (40.1,43.9)	43	50.4 (38.1,62.8)	12	31.7 (15.8,47.5)
Usually or always justified	348	10.9 (9.7,12.1)	22	28.0 (16.6,39.4)	15	30.6 (15.8,45.5)
Adjusted prevalence difference* (95% CI; q-value)	Referent		12.6 (1.2,24.0; 0.18)		15.8 (0.6,31.0; 0.20)	
To stop illegal immigration						
Never justified	2255	66.5 (64.7,68.2)	14	12.8 (6.0,19.7)	6	17.1 (3.9,30.4)
Sometimes justified	843	24.1 (22.5,25.6)	38	44.8 (32.3,57.2)	11	30.3 (13.4,47.1)
Usually or always justified	341	9.3 (8.3,10.4)	30	39.6 (27.1,52.2)	20	46.1 (28.4,63.7)
Adjusted prevalence difference* (95% CI; q-value)	Referent		29.8 (17.3,42.2; <0.001)		39.0 (21.4,56.5; <0.001)	
To keep borders open						
Never justified	2354	69.0 (67.3,70.8)	29	35.1 (23.3,47.0)	12	25.0 (10.7,39.4)
Sometimes justified	808	24.1 (22.5,25.7)	29	36.3 (23.9,48.8)	9	33.7 (13.3,54.0)
Usually or always justified	207	6.4 (5.5,7.4)	22	28.5 (16.9,40.2)	13	40.1 (21.1,59.0)
Adjusted prevalence difference* (95% CI; q-value)	Referent		20.9 (9.3,32.5; 0.01)		31.9 (13.2,50.7; 0.01)	
To stop a protest						
Never justified	2040	62.7 (60.9,64.6)	14	15.3 (7.1,23.5)	6	23.5 (6.8,40.2)
Sometimes justified	1133	32.9 (31.1,34.7)	44	51.4 (38.6,64.2)	14	35.4 (17.3,53.4)
Usually or always justified	141	4.1 (3.3,4.8)	19	30.6 (17.9,43.2)	12	33.5 (15.9,51.1)
Adjusted prevalence difference* (95% CI; q-value)	Referent		24.0 (12.0,36.0; 0.003)		30.5 (12.4,48.6; 0.01)	
To support a protest						
Never justified	2897	81.0 (79.5,82.5)	36	34.3 (23.3,45.3)	12	27.4 (11.7,43.1)
Sometimes justified	498	15.5 (14.2,16.9)	32	43.8 (31.0,56.6)	14	29.0 (14.1,43.9)
Usually or always justified	104	3.3 (2.6,4.0)	17	22.0 (11.6,32.3)	13	42.5 (24.0,61.1)
Adjusted prevalence difference* (95% CI; q-value)	Referent		17.5 (7.0,28.0; 0.01)		36.9 (18.5,55.3; 0.003)	

Table S8, continued.

What do you think about the use of force or violence in the following situations?	Militia Movement					
	Do Not Approve		Somewhat Approve		Strongly/Very Strongly Approve	
	Unweighted n	Weighted % (95% CI)	Unweighted n	Weighted % (95% CI)	Unweighted n	Weighted % (95% CI)
To stop voter fraud						
Never justified	1756	81.1 (79.3,83.0)	96	50.0 (41.7,58.4)	25	35.2 (22.0,48.3)
Sometimes justified	244	11.9 (10.3,13.5)	50	28.3 (20.8,35.9)	16	20.9 (10.5,31.3)
Usually or always justified	148	6.6 (5.4,7.7)	45	21.6 (14.8,28.5)	31	44.0 (30.1,57.9)
Adjusted prevalence difference* (95% CI; q-value)	Referent		13.8 (7.5,20.1; <0.001)		34.6 (21.4,47.8; <0.001)	
To stop voter intimidation						
Never justified	1352	63.7 (61.4,65.9)	91	46.1 (38.1,54.1)	27	32.3 (20.7,43.9)
Sometimes justified	588	27.3 (25.1,29.4)	67	31.6 (24.4,38.7)	21	37.8 (24.4,51.2)
Usually or always justified	202	9.1 (7.7,10.4)	44	21.1 (14.6,27.5)	22	29.9 (18.0,41.8)
Adjusted prevalence difference* (95% CI; q-value)	Referent		12.7 (5.9,19.4; 0.006)		21.4 (9.6,33.3; 0.006)	
To reinforce the police						
Never justified	1029	49.8 (47.5,52.2)	23	12.9 (7.5,18.3)	13	14.9 (6.3,23.6)
Sometimes justified	804	36.1 (33.8,38.4)	83	43.8 (35.7,52.0)	23	36.5 (22.6,50.3)
Usually or always justified	306	13.8 (12.2,15.5)	81	43.3 (35.0,51.5)	33	48.6 (34.7,62.5)
Adjusted prevalence difference* (95% CI; q-value)	Referent		28.2 (20.1,36.3; <0.001)		35.2 (21.4,48.9; <0.001)	
To stop police violence						
Never justified	1057	47.2 (44.9,49.6)	81	38.9 (31.1,46.6)	25	28.5 (17.5,39.5)
Sometimes justified	867	40.6 (38.2,42.9)	94	44.7 (36.6,52.7)	26	38.7 (25.8,51.7)
Usually or always justified	229	12.1 (10.5,13.7)	31	15.3 (9.7,20.9)	22	32.7 (19.9,45.5)
Adjusted prevalence difference* (95% CI; q-value)	Referent		0.5 (-5.7,6.6; 0.97)		16.2 (4.0,28.4; 0.09)	
To stop illegal immigration						
Never justified	1520	71.8 (69.7,73.9)	61	31.3 (23.9,38.7)	15	16.2 (7.9,24.5)
Sometimes justified	425	19.6 (17.8,21.5)	85	42.2 (34.1,50.3)	20	30.8 (18.3,43.4)
Usually or always justified	177	8.4 (7.1,9.8)	56	25.2 (18.4,32.1)	36	53.0 (39.8,66.2)
Adjusted prevalence difference* (95% CI; q-value)	Referent		15.7 (9.0,22.5; <0.001)		41.3 (28.1,54.4; <0.001)	
To keep borders open						
Never justified	1501	69.0 (66.8,71.2)	111	55.2 (47.0,63.4)	34	38.0 (25.4,50.6)
Sometimes justified	533	24.0 (22.0,26.0)	50	28.0 (20.5,35.5)	15	24.6 (11.9,37.2)
Usually or always justified	133	6.6 (5.4,7.8)	28	16.2 (9.9,22.5)	22	37.5 (23.5,51.4)
Adjusted prevalence difference* (95% CI; q-value)	Referent		9.0 (2.4,15.5; 0.10)		28.7 (15.4,41.9; <0.001)	
To stop a protest						
Never justified	1387	66.5 (64.3,68.7)	75	39.8 (31.8,47.9)	24	30.1 (18.1,42.0)
Sometimes justified	647	29.4 (27.2,31.5)	95	44.6 (36.4,52.8)	24	35.4 (22.4,48.5)
Usually or always justified	86	3.8 (2.9,4.7)	24	14.2 (7.7,20.7)	19	34.5 (20.6,48.5)
Adjusted prevalence difference* (95% CI; q-value)	Referent		10.2 (4.1,16.2; 0.01)		27.5 (14.8,40.3; <0.001)	
To support a protest						
Never justified	1809	81.3 (79.4,83.2)	140	67.8 (60.2,75.5)	32	38.6 (26.2,51.1)
Sometimes justified	303	15.4 (13.7,17.2)	43	23.2 (16.2,30.3)	19	27.1 (15.1,39.1)
Usually or always justified	59	3.2 (2.3,4.1)	16	9.0 (4.3,13.6)	24	34.3 (21.3,47.3)
Adjusted prevalence difference* (95% CI; q-value)	Referent		4.4 (-0.2,8.9; 0.40)		27.4 (14.6,40.3; <0.001)	

Table S8, continued.

What do you think about the use of force or violence in the following situations?	Boogaloo Movement					
	Do Not Approve		Somewhat Approve		Strongly/Very Strongly Approve	
	Unweighted n	Weighted % (95% CI)	Unweighted n	Weighted % (95% CI)	Unweighted n	Weighted % (95% CI)
To stop voter fraud						
Never justified	1082	80.1 (77.6,82.6)	29	44.7 (30.1,59.3)	4	15.5 (0.4,30.6)
Sometimes justified	144	11.4 (9.4,13.3)	19	32.9 (19.0,46.7)	11	34.8 (17.0,52.6)
Usually or always justified	101	8.0 (6.3,9.8)	11	21.4 (8.4,34.4)	16	49.7 (30.6,68.7)
Adjusted prevalence difference* (95% CI; q-value)	Referent		6.9 (-4.8,18.6; 0.48)		31.6 (13.1,50.1; 0.02)	
To stop voter intimidation						
Never justified	789	61.1 (58.1,64.0)	15	39.5 (21.7,57.2)	10	28.7 (9.6,47.8)
Sometimes justified	375	28.4 (25.7,31.1)	14	35.5 (17.9,53.2)	7	38.2 (15.2,61.2)
Usually or always justified	137	10.5 (8.6,12.3)	7	20.1 (4.4,35.7)	9	33.1 (12.8,53.4)
Adjusted prevalence difference* (95% CI; q-value)	Referent		12.3 (-4.3,29.0; 0.79)		22.4 (2.1,42.7; 0.79)	
To reinforce the police						
Never justified	653	51.0 (48.0,54.0)	11	25.9 (11.7,40.1)	7	25.7 (6.2,45.2)
Sometimes justified	471	34.6 (31.8,37.5)	19	41.5 (25.0,57.9)	7	30.7 (8.6,52.7)
Usually or always justified	183	14.1 (11.9,16.3)	13	32.6 (16.4,48.8)	11	43.7 (21.6,65.8)
Adjusted prevalence difference* (95% CI; q-value)	Referent		15.7 (-0.3,31.6; 0.16)		29.8 (9.5,50.1; 0.05)	
To stop police violence						
Never justified	645	47.0 (44.0,50.0)	15	23.5 (11.4,35.6)	5	14.4 (0.4,28.5)
Sometimes justified	532	40.3 (37.3,43.2)	26	50.2 (34.5,65.9)	13	45.1 (26.0,64.2)
Usually or always justified	145	12.6 (10.4,14.7)	12	22.9 (9.4,36.3)	14	40.5 (22.3,58.7)
Adjusted prevalence difference* (95% CI; q-value)	Referent		2.4 (-10.8,15.7; 0.81)		18.8 (-0.6,38.1; 0.21)	
To stop illegal immigration						
Never justified	969	73.4 (70.7,76.1)	22	42.1 (26.7,57.6)	4	8.6 (-0.6,17.9)
Sometimes justified	233	17.5 (15.3,19.8)	17	35.0 (19.1,50.9)	9	36.7 (17.1,56.3)
Usually or always justified	110	9.1 (7.2,10.9)	9	18.9 (7.1,30.7)	17	54.6 (34.7,74.5)
Adjusted prevalence difference* (95% CI; q-value)	Referent		5.3 (-6.6,17.1; 0.74)		37.8 (18.3,57.2; 0.004)	
To keep borders open						
Never justified	932	69.6 (66.8,72.4)	25	46.9 (30.3,63.4)	6	14.9 (3.0,26.8)
Sometimes justified	304	22.5 (20.0,25.0)	15	34.5 (18.1,50.9)	8	35.0 (14.2,55.8)
Usually or always justified	80	7.4 (5.6,9.2)	8	18.6 (4.5,32.7)	13	50.1 (29.3,70.9)
Adjusted prevalence difference* (95% CI; q-value)	Referent		6.1 (-6.4,18.5; 0.65)		39.7 (19.7,59.7; 0.003)	
To stop a protest						
Never justified	880	68.6 (65.7,71.4)	18	31.4 (17.2,45.5)	3	12.4 (-1.3,26.0)
Sometimes justified	347	26.2 (23.5,28.8)	24	47.2 (31.0,63.5)	9	36.8 (15.7,58.0)
Usually or always justified	58	4.8 (3.4,6.2)	8	17.6 (4.2,30.9)	12	50.8 (28.9,72.7)
Adjusted prevalence difference* (95% CI; q-value)	Referent		8.5 (-2.8,19.8; 0.56)		38.5 (18.7,58.3; 0.004)	
To support a protest						
Never justified	1099	79.3 (76.7,81.8)	20	38.8 (23.2,54.4)	10	26.2 (9.6,42.8)
Sometimes justified	198	16.5 (14.2,18.8)	20	47.1 (30.7,63.6)	8	27.6 (9.0,46.2)
Usually or always justified	46	4.2 (2.9,5.5)	6	14.1 (2.4,25.7)	15	46.2 (27.0,65.5)
Adjusted prevalence difference* (95% CI; q-value)	Referent		6.5 (-4.8,17.8; 0.54)		36.7 (18.0,55.3; 0.004)	

* Prevalence differences are adjusted for age, race and ethnicity, gender, education, income, Census division, and rurality and are for the usually or always justified comparison. They are expressed in absolute percentage points. Q-values represent the probability that the given difference would be a false discovery; they represent the expected proportion of “false positives” that would be seen among the collection of all differences whose q-values were at or below the given q-value.

Table S9. Approval of individual organizations and movements and personal willingness to engage in political violence, by type of violence

In a situation where you think force or violence is justified to advance an important political objective…How willing would you personally be to use force or violence in each of these ways?	Proud Boys					
	Do Not Approve		Somewhat Approve		Strongly/Very Strongly Approve	
	Unweighted n	Weighted % (95% CI)	Unweighted n	Weighted % (95% CI)	Unweighted n	Weighted % (95% CI)
To damage property						
Not asked the question	1253	25.8 (24.5,27.2)	20	6.5 (3.6,9.4)	11	6.3 (2.2,10.4)
Not willing	3182	63.7 (62.2,65.2)	239	69.7 (63.7,75.7)	96	57.3 (48.4,66.2)
Sometimes willing	345	7.8 (6.9,8.6)	42	13.8 (9.4,18.3)	20	15.3 (8.1,22.5)
Very or completely willing	90	2.4 (1.8,2.9)	21	9.5 (5.1,13.9)	32	19.7 (12.7,26.8)
Adjusted prevalence difference* (95% CI; q-value)	Referent		6.4 (2.3,10.5; 0.01)		16.0 (9.1,23.0; <0.001)	
To threaten or intimidate a person						
Not asked the question	1253	25.8 (24.5,27.2)	20	6.5 (3.6,9.4)	11	6.3 (2.2,10.4)
Not willing	3323	66.8 (65.3,68.3)	232	67.6 (61.5,73.7)	100	56.9 (47.9,65.9)
Sometimes willing	238	5.7 (4.9,6.5)	57	19.5 (14.2,24.7)	22	17.5 (9.9,25.1)
Very or completely willing	55	1.4 (1.0,1.8)	14	6.2 (2.7,9.8)	26	17.3 (10.4,24.1)
Adjusted prevalence difference* (95% CI; q-value)	Referent		4.3 (0.8,7.8; 0.15)		15.0 (8.2,21.7; <0.001)	
To injure a person						
Not asked the question	1253	25.8 (24.5,27.2)	20	6.5 (3.6,9.4)	11	6.3 (2.2,10.4)
Not willing	3357	67.7 (66.2,69.2)	237	69.0 (62.9,75.1)	102	59.2 (50.3,68.2)
Sometimes willing	200	4.8 (4.0,5.5)	47	15.9 (10.9,20.8)	19	13.9 (7.0,20.8)
Very or completely willing	52	1.3 (0.9,1.7)	19	8.4 (4.4,12.3)	27	19.3 (11.9,26.7)
Adjusted prevalence difference* (95% CI; q-value)	Referent		6.5 (2.6,10.4; 0.01)		17.0 (10.0,24.0; <0.001)	
To kill a person						
Not asked the question	1253	25.8 (24.5,27.2)	20	6.5 (3.6,9.4)	11	6.3 (2.2,10.4)
Not willing	3461	70.0 (68.5,71.5)	253	73.2 (67.2,79.2)	108	65.4 (56.8,73.9)
Sometimes willing	96	2.4 (1.9,3.0)	31	11.3 (6.8,15.7)	13	8.3 (3.2,13.4)
Very or completely willing	56	1.3 (1.0,1.7)	19	7.7 (4.0,11.5)	27	18.0 (11.0,24.9)
Adjusted prevalence difference* (95% CI; q-value)	Referent		5.8 (2.1,9.5; 0.03)		15.9 (9.2,22.6; <0.001)	

Table S9, continued.

In a situation where you think force or violence is justified to advance an important political objective…How willing would you personally be to use force or violence in each of these ways?	Oath Keepers					
	Do Not Approve		Somewhat Approve		Strongly/Very Strongly Approve	
	Unweighted n	Weighted % (95% CI)	Unweighted n	Weighted % (95% CI)	Unweighted n	Weighted % (95% CI)
To damage property						
Not asked the question	953	27.4 (25.7,29.0)	25	8.5 (4.9,12.2)	13	5.9 (2.5,9.4)
Not willing	2175	62.2 (60.3,64.0)	232	67.3 (61.0,73.6)	143	61.8 (54.3,69.4)
Sometimes willing	258	8.4 (7.3,9.5)	40	13.3 (9.0,17.5)	22	12.3 (6.7,18.0)
Very or completely willing	54	1.9 (1.3,2.4)	22	10.6 (5.7,15.5)	37	18.5 (12.5,24.5)
Adjusted prevalence difference* (95% CI; q-value)	Referent		8.1 (3.9,12.4; 0.002)		15.8 (9.8,21.8; <0.001)	
To threaten or intimidate a person						
Not asked the question	953	27.4 (25.7,29.0)	25	8.5 (4.9,12.2)	13	5.9 (2.5,9.4)
Not willing	2308	66.3 (64.5,68.0)	224	65.4 (59.1,71.6)	138	56.1 (48.3,63.8)
Sometimes willing	150	5.0 (4.1,5.9)	53	18.3 (13.2,23.4)	29	18.0 (11.3,24.6)
Very or completely willing	30	1.1 (0.7,1.6)	18	7.6 (3.6,11.7)	34	17.7 (11.7,23.7)
Adjusted prevalence difference* (95% CI; q-value)	Referent		5.8 (2.1,9.5; 0.02)		15.1 (9.1,21.1; <0.001)	
To injure a person						
Not asked the question	953	27.4 (25.7,29.0)	25	8.5 (4.9,12.2)	13	5.9 (2.5,9.4)
Not willing	2317	66.5 (64.8,68.3)	233	67.8 (61.6,74.0)	143	59.7 (52.0,67.4)
Sometimes willing	130	4.5 (3.6,5.4)	47	16.7 (11.7,21.7)	30	15.8 (9.7,22.0)
Very or completely willing	32	1.2 (0.7,1.6)	14	6.3 (2.6,10.1)	30	17.4 (11.2,23.6)
Adjusted prevalence difference* (95% CI; q-value)	Referent		4.3 (0.9,7.8; 0.10)		14.9 (8.8,20.9; <0.001)	
To kill a person						
Not asked the question	953	27.4 (25.7,29.0)	25	8.5 (4.9,12.2)	13	5.9 (2.5,9.4)
Not willing	2377	68.5 (66.7,70.2)	247	72.5 (66.5,78.5)	151	66.8 (59.6,74.1)
Sometimes willing	70	2.6 (1.9,3.2)	30	10.8 (6.6,15.1)	15	6.9 (3.0,10.8)
Very or completely willing	36	1.2 (0.7,1.6)	17	7.5 (3.6,11.5)	36	18.2 (12.2,24.2)
Adjusted prevalence difference* (95% CI; q-value)	Referent		5.3 (1.7,8.9; 0.04)		16.3 (10.3,22.3; <0.001)	

Table S9, continued.

**In a situation where you think force or violence is justified to advance an important political objective…How willing would you personally be to use force or violence in each of these ways?**	**Three Percenters**					
	**Do Not Approve**		**Somewhat Approve**		**Strongly/Very Strongly Approve**	
	**Unweighted n**	**Weighted % (95% CI)**	**Unweighted n**	**Weighted % (95% CI)**	**Unweighted n**	**Weighted % (95% CI)**
To damage property						
Not asked the question	634	25.8 (23.8,27.7)	15	7.4 (3.6,11.3)	11	7.6 (2.7,12.6)
Not willing	1534	62.1 (59.9,64.3)	135	64.4 (56.5,72.3)	56	47.1 (36.9,57.3)
Sometimes willing	195	9.0 (7.6,10.3)	31	16.3 (10.4,22.3)	19	18.7 (10.0,27.4)
Very or completely willing	52	2.8 (1.9,3.6)	15	11.5 (5.3,17.8)	32	26.6 (17.8,35.4)
Adjusted prevalence difference* (95% CI; q-value)	Referent		6.9 (1.4,12.3; 0.08)		21.4 (12.5,30.2; <0.001)	
To threaten or intimidate a person						
Not asked the question	634	25.8 (23.8,27.7)	15	7.4 (3.6,11.3)	11	7.6 (2.7,12.6)
Not willing	1634	66.6 (64.4,68.7)	130	62.0 (54.1,69.9)	52	39.9 (30.1,49.7)
Sometimes willing	117	5.5 (4.4,6.6)	35	19.7 (12.9,26.5)	29	31.3 (21.2,41.4)
Very or completely willing	29	1.8 (1.0,2.6)	17	10.9 (5.4,16.4)	25	19.6 (11.8,27.3)
Adjusted prevalence difference* (95% CI; q-value)	Referent		7.5 (2.2,12.8; 0.07)		15.8 (7.8,23.9; 0.003)	
To injure a person						
Not asked the question	634	25.8 (23.8,27.7)	15	7.4 (3.6,11.3)	11	7.6 (2.7,12.6)
Not willing	1648	67.1 (65.0,69.2)	135	63.9 (56.1,71.8)	51	40.4 (30.5,50.2)
Sometimes willing	97	4.7 (3.7,5.7)	34	20.7 (13.6,27.7)	28	25.9 (16.4,35.3)
Very or completely willing	31	1.9 (1.1,2.6)	13	8.0 (3.5,12.5)	28	26.1 (17.0,35.2)
Adjusted prevalence difference* (95% CI; q-value)	Referent		4.4 (-0.2,9.0; 0.29)		21.5 (12.8,30.2; <0.001)	
To kill a person						
Not asked the question	634	25.8 (23.8,27.7)	15	7.4 (3.6,11.3)	11	7.6 (2.7,12.6)
Not willing	1682	68.7 (66.6,70.8)	141	67.6 (59.9,75.3)	61	52.2 (42.0,62.4)
Sometimes willing	57	2.8 (2.0,3.7)	23	14.6 (8.1,21.0)	13	11.1 (4.7,17.6)
Very or completely willing	37	2.0 (1.3,2.8)	18	10.4 (5.4,15.4)	32	27.4 (18.5,36.4)
Adjusted prevalence difference* (95% CI; q-value)	Referent		7.0 (2.1,11.9; 0.08)		23.9 (15.0,32.8; <0.001)	

Table S9, continued.

In a situation where you think force or violence is justified to advance an important political objective…How willing would you personally be to use force or violence in each of these ways?	QAnon					
	Do Not Approve		Somewhat Approve		Strongly/Very Strongly Approve	
	Unweighted n	Weighted % (95% CI)	Unweighted n	Weighted % (95% CI)	Unweighted n	Weighted % (95% CI)
To damage property						
Not asked the question	1194	24.9 (23.6,26.3)	20	8.1 (4.4,11.8)	7	5.9 (1.1,10.7)
Not willing	3103	64.5 (62.9,66.0)	138	64.0 (56.6,71.4)	63	47.4 (36.8,57.9)
Sometimes willing	346	8.1 (7.2,9.0)	28	13.4 (8.3,18.5)	18	17.6 (8.9,26.3)
Very or completely willing	84	2.2 (1.7,2.7)	20	13.3 (7.5,19.2)	29	28.4 (18.3,38.4)
Adjusted prevalence difference* (95% CI; q-value)	Referent		10.5 (4.9,16.1; 0.002)		24.3 (14.9,33.8; <0.001)	
To threaten or intimidate a person						
Not asked the question	1194	24.9 (23.6,26.3)	20	8.1 (4.4,11.8)	7	5.9 (1.1,10.7)
Not willing	3246	67.7 (66.1,69.2)	141	65.3 (58.0,72.6)	60	42.1 (31.9,52.2)
Sometimes willing	237	5.8 (5.0,6.7)	35	17.8 (11.9,23.6)	26	29.0 (18.5,39.5)
Very or completely willing	48	1.2 (0.8,1.6)	12	8.2 (3.4,13.1)	24	21.3 (12.4,30.2)
Adjusted prevalence difference* (95% CI; q-value)	Referent		5.6 (1.3,9.9; 0.10)		19.1 (10.3,28.0; <0.001)	
To injure a person						
Not asked the question	1194	24.9 (23.6,26.3)	20	8.1 (4.4,11.8)	7	5.9 (1.1,10.7)
Not willing	3283	68.6 (67.1,70.1)	146	69.6 (62.6,76.5)	63	45.5 (35.1,55.9)
Sometimes willing	202	5.0 (4.2,5.8)	29	14.0 (8.8,19.3)	16	17.2 (7.9,26.6)
Very or completely willing	43	1.1 (0.7,1.4)	12	7.1 (2.9,11.3)	31	30.6 (20.6,40.7)
Adjusted prevalence difference* (95% CI; q-value)	Referent		5.6 (1.5,9.7; 0.08)		28.6 (18.8,38.3; <0.001)	
To kill a person						
Not asked the question	1194	24.9 (23.6,26.3)	20	8.1 (4.4,11.8)	7	5.9 (1.1,10.7)
Not willing	3374	70.6 (69.2,72.1)	151	71.0 (63.9,78.1)	69	54.8 (44.1,65.5)
Sometimes willing	105	2.8 (2.2,3.4)	21	10.4 (5.7,15.1)	11	10.2 (3.0,17.4)
Very or completely willing	52	1.2 (0.9,1.6)	14	7.4 (3.2,11.5)	30	27.4 (17.8,36.9)
Adjusted prevalence difference* (95% CI; q-value)	Referent		6.0 (1.8,10.1; 0.07)		25.7 (16.3,35.1; <0.001)	

Table S9, continued.

In a situation where you think force or violence is justified to advance an important political objective…How willing would you personally be to use force or violence in each of these ways?	Christian Nationalist Movement					
	Do Not Approve		Somewhat Approve		Strongly/Very Strongly Approve	
	Unweighted n	Weighted % (95% CI)	Unweighted n	Weighted % (95% CI)	Unweighted n	Weighted % (95% CI)
To damage property						
Not asked the question	884	26.3 (24.7,28.0)	64	11.2 (8.2,14.1)	26	9.1 (5.2,13.1)
Not willing	2062	62.1 (60.3,64.0)	430	72.4 (68.0,76.8)	247	64.6 (58.7,70.6)
Sometimes willing	260	8.6 (7.5,9.7)	52	10.0 (7.1,13.0)	39	12.4 (8.2,16.6)
Very or completely willing	72	2.7 (2.1,3.4)	21	5.7 (2.9,8.5)	44	13.8 (9.6,18.1)
Adjusted prevalence difference* (95% CI; q-value)	Referent		2.9 (0.4,5.5; 0.12)		10.6 (6.4,14.8; <0.001)	
To threaten or intimidate a person						
Not asked the question	884	26.3 (24.7,28.0)	64	11.2 (8.2,14.1)	26	9.1 (5.2,13.1)
Not willing	2173	65.7 (63.9,67.6)	427	71.2 (66.7,75.6)	259	66.8 (60.8,72.8)
Sometimes willing	186	6.4 (5.4,7.4)	61	13.0 (9.5,16.4)	33	11.6 (7.2,16.0)
Very or completely willing	35	1.3 (0.8,1.8)	16	4.2 (1.9,6.6)	37	12.2 (8.1,16.3)
Adjusted prevalence difference* (95% CI; q-value)	Referent		2.7 (0.5,5.0; 0.10)		10.1 (6.1,14.2; <0.001)	
To injure a person						
Not asked the question	884	26.3 (24.7,28.0)	64	11.2 (8.2,14.1)	26	9.1 (5.2,13.1)
Not willing	2208	66.8 (65.0,68.6)	444	74.7 (70.4,79.1)	265	69.9 (64.0,75.7)
Sometimes willing	148	5.2 (4.3,6.1)	37	7.5 (4.7,10.3)	31	9.8 (5.8,13.8)
Very or completely willing	31	1.2 (0.7,1.7)	22	5.9 (3.2,8.6)	33	11.0 (7.1,14.9)
Adjusted prevalence difference* (95% CI; q-value)	Referent		4.6 (2.1,7.2; 0.004)		9.1 (5.2,13.0; <0.001)	
To kill a person						
Not asked the question	884	26.3 (24.7,28.0)	64	11.2 (8.2,14.1)	26	9.1 (5.2,13.1)
Not willing	2277	69.1 (67.4,70.9)	452	76.0 (71.6,80.4)	276	73.9 (68.4,79.4)
Sometimes willing	77	2.8 (2.1,3.5)	29	6.3 (3.6,9.1)	20	6.1 (3.1,9.1)
Very or completely willing	36	1.3 (0.8,1.8)	22	5.2 (2.7,7.7)	34	10.9 (7.1,14.6)
Adjusted prevalence difference* (95% CI; q-value)	Referent		3.8 (1.4,6.2; 0.02)		8.6 (4.9,12.4; <0.001)	

Table S9, continued.

**In a situation where you think force or violence is justified to advance an important political objective…How willing would you personally be to use force or violence in each of these ways?**	**White Supremacy Movement**					
	**Do Not Approve**		**Somewhat Approve**		**Strongly/Very Strongly Approve**	
	**Unweighted n**	**Weighted % (95% CI)**	**Unweighted n**	**Weighted % (95% CI)**	**Unweighted n**	**Weighted % (95% CI)**
To damage property						
Not asked the question	1542	22.5 (21.4,23.6)	6	5.1 (0.9,9.2)	5	7.3 (-0.3,14.9)
Not willing	4694	67.8 (66.5,69.0)	101	56.6 (47.5,65.6)	32	36.5 (24.3,48.7)
Sometimes willing	457	7.3 (6.6,8.0)	37	20.9 (13.9,27.8)	11	21.3 (9.0,33.6)
Very or completely willing	119	2.1 (1.7,2.5)	18	16.8 (8.7,24.9)	25	34.9 (22.6,47.2)
Adjusted prevalence difference* (95% CI; q-value)	Referent		13.4 (5.8,21.1; 0.006)		31.3 (19.2,43.3; <0.001)	
To threaten or intimidate a person						
Not asked the question	1542	22.5 (21.4,23.6)	6	5.1 (0.9,9.2)	5	7.3 (-0.3,14.9)
Not willing	4812	69.6 (68.4,70.8)	100	54.2 (45.1,63.2)	31	34.7 (22.7,46.7)
Sometimes willing	381	6.2 (5.5,6.9)	39	25.6 (17.6,33.7)	16	30.1 (17.0,43.2)
Very or completely willing	75	1.3 (1.0,1.7)	16	14.1 (6.7,21.4)	21	27.8 (16.5,39.2)
Adjusted prevalence difference* (95% CI; q-value)	Referent		11.7 (4.5,18.9; 0.02)		25.6 (14.4,36.8; <0.001)	
To injure a person						
Not asked the question	1542	22.5 (21.4,23.6)	6	5.1 (0.9,9.2)	5	7.3 (-0.3,14.9)
Not willing	4872	70.5 (69.3,71.7)	104	56.4 (47.3,65.5)	33	39.8 (27.2,52.5)
Sometimes willing	312	5.2 (4.5,5.8)	33	21.8 (14.1,29.5)	13	21.1 (9.1,33.0)
Very or completely willing	77	1.4 (1.0,1.7)	19	16.0 (8.6,23.5)	22	31.8 (19.7,43.8)
Adjusted prevalence difference* (95% CI; q-value)	Referent		13.6 (6.4,20.9; 0.003)		29.3 (17.4,41.2; <0.001)	
To kill a person						
Not asked the question	1542	22.5 (21.4,23.6)	6	5.1 (0.9,9.2)	5	7.3 (-0.3,14.9)
Not willing	5020	73.1 (71.9,74.3)	111	61.5 (52.4,70.5)	34	44.4 (31.2,57.5)
Sometimes willing	158	2.6 (2.2,3.0)	23	15.8 (8.7,22.8)	11	17.3 (6.9,27.7)
Very or completely willing	90	1.4 (1.1,1.7)	22	17.0 (9.7,24.4)	23	31.0 (19.3,42.8)
Adjusted prevalence difference* (95% CI; q-value)	Referent		14.9 (7.6,22.2; <0.001)		29.0 (17.4,40.6; <0.001)	

Table S9, continued.

In a situation where you think force or violence is justified to advance an important political objective…How willing would you personally be to use force or violence in each of these ways?	Militia Movement					
	Do Not Approve		Somewhat Approve		Strongly/Very Strongly Approve	
	Unweighted n	Weighted % (95% CI)	Unweighted n	Weighted % (95% CI)	Unweighted n	Weighted % (95% CI)
To damage property						
Not asked the question	1081	25.2 (23.8,26.7)	23	6.7 (3.5,9.8)	9	4.9 (1.6,8.1)
Not willing	2853	65.2 (63.6,66.8)	304	74.6 (69.3,79.9)	76	51.3 (41.7,60.9)
Sometimes willing	284	7.1 (6.2,8.0)	50	14.1 (9.8,18.4)	18	14.1 (6.8,21.5)
Very or completely willing	72	2.2 (1.6,2.7)	13	3.7 (1.3,6.0)	38	28.8 (19.9,37.7)
Adjusted prevalence difference* (95% CI; q-value)	Referent		0.2 (-2.5,3.0; 0.90)		25.0 (16.2,33.8; <0.001)	
To threaten or intimidate a person						
Not asked the question	1081	25.2 (23.8,26.7)	23	6.7 (3.5,9.8)	9	4.9 (1.6,8.1)
Not willing	2959	67.7 (66.1,69.3)	293	72.6 (67.4,77.9)	69	45.7 (36.2,55.1)
Sometimes willing	201	5.3 (4.5,6.1)	63	15.5 (11.5,19.5)	31	26.0 (16.9,35.1)
Very or completely willing	48	1.5 (1.0,2.0)	11	3.8 (1.3,6.3)	32	22.6 (14.7,30.6)
Adjusted prevalence difference* (95% CI; q-value)	Referent		1.7 (-1.1,4.4; 0.67)		20.0 (11.7,28.2; <0.001)	
To injure a person						
Not asked the question	1081	25.2 (23.8,26.7)	23	6.7 (3.5,9.8)	9	4.9 (1.6,8.1)
Not willing	3008	69.1 (67.6,70.7)	291	72.4 (67.1,77.6)	74	49.2 (39.7,58.8)
Sometimes willing	149	3.9 (3.2,4.6)	65	15.7 (11.6,19.8)	24	19.0 (10.8,27.2)
Very or completely willing	46	1.3 (0.9,1.7)	10	3.8 (1.2,6.4)	34	26.1 (17.5,34.7)
Adjusted prevalence difference* (95% CI; q-value)	Referent		2.0 (-0.8,4.8; 0.45)		24.2 (15.6,32.8; <0.001)	
To kill a person						
Not asked the question	1081	25.2 (23.8,26.7)	23	6.7 (3.5,9.8)	9	4.9 (1.6,8.1)
Not willing	3084	71.0 (69.5,72.5)	308	76.2 (71.0,81.4)	82	55.4 (45.9,65.0)
Sometimes willing	74	2.1 (1.6,2.6)	43	10.6 (7.1,14.0)	13	11.3 (4.5,18.1)
Very or completely willing	49	1.3 (0.9,1.8)	14	3.7 (1.4,6.0)	37	27.5 (19.0,36.1)
Adjusted prevalence difference* (95% CI; q-value)	Referent		1.8 (-0.8,4.4; 0.51)		25.8 (17.2,34.4; <0.001)	

Table S9, continued.

**In a situation where you think force or violence is justified to advance an important political objective…How willing would you personally be to use force or violence in each of these ways?**	**Boogaloo Movement**					
	**Do Not Approve**		**Somewhat Approve**		**Strongly/Very Strongly Approve**	
	**Unweighted n**	**Weighted % (95% CI)**	**Unweighted n**	**Weighted % (95% CI)**	**Unweighted n**	**Weighted % (95% CI)**
To damage property						
Not asked the question	661	24.8 (23.0,26.7)	13	11.9 (5.1,18.6)	1	1.1 (-1.0,3.2)
Not willing	1688	63.1 (61.0,65.1)	51	50.1 (38.6,61.7)	17	32.1 (18.3,45.9)
Sometimes willing	213	8.7 (7.5,9.9)	20	20.3 (11.1,29.4)	13	23.5 (11.3,35.7)
Very or completely willing	66	3.1 (2.3,3.9)	12	16.7 (7.0,26.4)	26	43.3 (28.9,57.7)
Adjusted prevalence difference* (95% CI; q-value)	Referent		11.5 (2.4,20.6; 0.07)		36.0 (22.2,49.8; <0.001)	
To threaten or intimidate a person						
Not asked the question	661	24.8 (23.0,26.7)	13	11.9 (5.1,18.6)	1	1.1 (-1.0,3.2)
Not willing	1774	66.2 (64.2,68.2)	50	49.0 (37.4,60.5)	17	27.2 (14.6,39.9)
Sometimes willing	150	6.6 (5.4,7.7)	20	20.9 (11.6,30.1)	17	38.1 (23.4,52.7)
Very or completely willing	43	2.1 (1.4,2.8)	11	14.7 (5.5,23.9)	22	33.6 (20.3,46.9)
Adjusted prevalence difference* (95% CI; q-value)	Referent		11.3 (2.5,20.2; 0.16)		28.8 (15.8,41.8; <0.001)	
To injure a person						
Not asked the question	661	24.8 (23.0,26.7)	13	11.9 (5.1,18.6)	1	1.1 (-1.0,3.2)
Not willing	1800	67.4 (65.3,69.4)	49	47.2 (35.7,58.7)	19	33.0 (19.2,46.8)
Sometimes willing	125	5.5 (4.5,6.6)	25	25.8 (15.9,35.7)	9	19.0 (6.4,31.6)
Very or completely willing	35	1.7 (1.1,2.3)	9	14.0 (4.7,23.4)	28	46.9 (32.5,61.4)
Adjusted prevalence difference* (95% CI; q-value)	Referent		10.8 (2.2,19.4; 0.18)		42.6 (28.5,56.8; <0.001)	
To kill a person						
Not asked the question	661	24.8 (23.0,26.7)	13	11.9 (5.1,18.6)	1	1.1 (-1.0,3.2)
Not willing	1853	69.8 (67.8,71.8)	53	48.4 (36.9,59.9)	18	33.7 (19.6,47.8)
Sometimes willing	71	3.2 (2.3,4.0)	18	18.0 (9.4,26.6)	11	23.8 (10.3,37.2)
Very or completely willing	39	1.8 (1.2,2.4)	10	15.3 (5.7,25.0)	27	41.5 (27.6,55.3)
Adjusted prevalence difference* (95% CI; q-value)	Referent		13.9 (4.7,23.1; 0.04)		38.7 (25.3,52.1; <0.001)	

* Prevalence differences are adjusted for age, race and ethnicity, gender, education, income, Census division, and rurality and are for the very or completely willing comparison. They are expressed in absolute percentage points. Q-values represent the probability that the given difference would be a false discovery; they represent the expected proportion of "false positives" that would be seen among the collection of all differences whose q-values were at or below the given q-value.

Table S10. Approval of individual organizations and movements and personal willingness to engage in political violence, by target of violence

In a situation where you think force or violence is justified to advance an important political objective…How willing would you personally be to use force or violence against a person because they are…	Proud Boys					
	Do Not Approve		Somewhat Approve		Strongly/Very Strongly Approve	
	Unweighted n	Weighted % (95% CI)	Unweighted n	Weighted % (95% CI)	Unweighted n	Weighted % (95% CI)
An elected federal or state government official						
Not asked the question	1253	25.8 (24.5,27.2)	20	6.5 (3.6,9.4)	11	6.3 (2.2,10.4)
Not willing	3416	69.0 (67.6,70.5)	235	67.8 (61.6,73.9)	99	57.7 (48.7,66.7)
Sometimes willing	153	3.5 (2.9,4.1)	53	19.9 (14.5,25.3)	17	13.6 (6.7,20.5)
Very or completely willing	41	1.1 (0.7,1.5)	11	4.7 (1.4,8.1)	31	20.8 (13.1,28.4)
Adjusted prevalence difference* (95% CI; q-value)	Referent		2.9 (-0.5,6.2; 0.52)		18.3 (10.8,25.8; <0.001)	
An elected local government official						
Not asked the question	1253	25.8 (24.5,27.2)	20	6.5 (3.6,9.4)	11	6.3 (2.2,10.4)
Not willing	3434	69.4 (68.0,70.9)	241	71.3 (65.5,77.0)	99	57.7 (48.7,66.7)
Sometimes willing	129	3.1 (2.6,3.7)	47	16.5 (11.7,21.2)	23	16.6 (9.3,24.0)
Very or completely willing	40	1.0 (0.7,1.4)	11	4.7 (1.6,7.8)	25	17.0 (10.1,23.8)
Adjusted prevalence difference* (95% CI; q-value)	Referent		3.3 (0.2,6.5; 0.29)		15.3 (8.5,22.2; <0.001)	
An election worker, such as a poll worker or vote counter						
Not asked the question	1253	25.8 (24.5,27.2)	20	6.5 (3.6,9.4)	11	6.3 (2.2,10.4)
Not willing	3519	71.6 (70.1,73.0)	257	74.5 (68.7,80.4)	104	60.1 (51.1,69.0)
Sometimes willing	56	1.3 (0.9,1.7)	33	12.4 (7.8,17.0)	17	12.6 (6.0,19.2)
Very or completely willing	30	0.7 (0.4,1.0)	10	5.6 (1.9,9.3)	27	20.0 (12.4,27.6)
Adjusted prevalence difference* (95% CI; q-value)	Referent		4.5 (0.9,8.1; 0.09)		18.3 (11.0,25.5; <0.001)	
A public health official						
Not asked the question	1253	25.8 (24.5,27.2)	20	6.5 (3.6,9.4)	11	6.3 (2.2,10.4)
Not willing	3499	71.1 (69.6,72.5)	249	73.4 (67.6,79.2)	102	59.1 (50.1,68.0)
Sometimes willing	74	1.8 (1.3,2.3)	41	14.1 (9.5,18.8)	19	13.3 (7.0,19.7)
Very or completely willing	29	0.7 (0.4,1.0)	10	5.0 (1.7,8.3)	25	18.5 (10.9,26.1)
Adjusted prevalence difference* (95% CI; q-value)	Referent		3.9 (0.6,7.1; 0.18)		17.4 (10.0,24.7; <0.001)	
A member of the military or National Guard						
Not asked the quesion	1253	25.8 (24.5,27.2)	20	6.5 (3.6,9.4)	11	6.3 (2.2,10.4)
Not willing	3422	69.3 (67.8,70.7)	254	73.6 (67.8,79.4)	106	60.5 (51.5,69.5)
Sometimes willing	142	3.3 (2.7,3.8)	36	12.7 (8.4,17.0)	15	10.9 (5.1,16.6)
Very or completely willing	39	1.1 (0.7,1.5)	12	6.8 (2.7,10.8)	27	21.3 (13.1,29.5)
Adjusted prevalence difference* (95% CI; q-value)	Referent		4.5 (0.7,8.4; 0.11)		18.3 (10.4,26.2; <0.001)	
A police officer						
Not asked the quesion	1253	25.8 (24.5,27.2)	20	6.5 (3.6,9.4)	11	6.3 (2.2,10.4)
Not willing	3363	67.6 (66.1,69.1)	253	73.6 (67.8,79.4)	112	66.5 (57.8,75.1)
Sometimes willing	183	4.4 (3.7,5.1)	37	13.8 (9.1,18.4)	11	7.7 (2.8,12.6)
Very or completely willing	60	1.7 (1.2,2.1)	10	5.2 (1.8,8.7)	25	18.5 (11.0,26.0)
Adjusted prevalence difference* (95% CI; q-value)	Referent		1.9 (-1.7,5.6; 0.73)		14.7 (7.0,22.3; 0.003)	
A person who does not share your race or ethnicity						
Not asked the quesion	1253	25.8 (24.5,27.2)	20	6.5 (3.6,9.4)	11	6.3 (2.2,10.4)
Not willing	3520	71.4 (69.9,72.8)	254	73.8 (67.9,79.6)	109	60.4 (51.3,69.4)
Sometimes willing	55	1.4 (1.0,1.8)	37	13.4 (8.7,18.0)	13	13.0 (6.0,20.0)
Very or completely willing	30	0.8 (0.5,1.1)	11	5.9 (2.1,9.6)	26	19.3 (11.6,26.9)
Adjusted prevalence difference* (95% CI; q-value)	Referent		4.2 (0.7,7.8; 0.14)		17.1 (9.8,24.4; <0.001)	
A person who does not share your religion						
Not asked the quesion	1253	25.8 (24.5,27.2)	20	6.5 (3.6,9.4)	11	6.3 (2.2,10.4)
Not willing	3518	71.4 (69.9,72.8)	255	74.5 (68.8,80.2)	112	64.5 (55.7,73.4)
Sometimes willing	48	1.3 (0.9,1.7)	36	13.8 (9.2,18.5)	16	13.0 (6.1,19.9)
Very or completely willing	33	0.8 (0.5,1.2)	9	4.3 (1.2,7.4)	21	15.6 (8.8,22.4)
Adjusted prevalence difference* (95% CI; q-value)	Referent		3.0 (-0.1,6.1; 0.24)		13.6 (7.1,20.0; 0.001)	
A person who does not share your political beliefs						
Not asked the quesion	1253	25.8 (24.5,27.2)	20	6.5 (3.6,9.4)	11	6.3 (2.2,10.4)
Not willing	3474	70.4 (68.9,71.8)	251	73.5 (67.6,79.4)	112	66.2 (57.5,74.9)
Sometimes willing	110	2.7 (2.1,3.3)	40	15.7 (10.5,21.0)	12	7.6 (2.9,12.3)
Very or completely willing	25	0.6 (0.4,0.9)	7	2.6 (0.5,4.6)	24	18.9 (11.2,26.6)
Adjusted prevalence difference* (95% CI; q-value)	Referent		1.6 (-0.5,3.6; 0.46)		17.6 (10.2,25.0; <0.001)	

Table S10, continued.

In a situation where you think force or violence is justified to advance an important political objective...How willing would <u>you personally</u> be to use force or violence against a person because they are...	Oath Keepers					
	Do Not Approve		Somewhat Approve		Strongly/Very Strongly Approve	
	Unweighted n	Weighted % (95% CI)	Unweighted n	Weighted % (95% CI)	Unweighted n	Weighted % (95% CI)
An elected federal or state government official						
Not asked the question	953	27.4 (25.7,29.0)	25	8.5 (4.9,12.2)	13	5.9 (2.5,9.4)
Not willing	2362	67.9 (66.1,69.6)	235	68.5 (62.3,74.7)	138	60.5 (53.0,68.1)
Sometimes willing	95	3.3 (2.6,4.0)	41	14.7 (10.2,19.2)	29	15.7 (9.6,21.7)
Very or completely willing	26	1.1 (0.6,1.6)	15	7.2 (2.9,11.6)	35	16.4 (10.8,21.9)
Adjusted prevalence difference* (95% CI; q-value)	Referent		5.2 (1.2,9.1; 0.07)		13.9 (8.3,19.5; <0.001)	
An elected local government official						
Not asked the question	953	27.4 (25.7,29.0)	25	8.5 (4.9,12.2)	13	5.9 (2.5,9.4)
Not willing	2379	68.4 (66.7,70.1)	232	68.8 (62.7,74.8)	140	60.3 (52.7,67.9)
Sometimes willing	76	2.9 (2.2,3.6)	44	14.9 (10.5,19.3)	28	15.0 (9.0,21.1)
Very or completely willing	23	0.8 (0.5,1.2)	14	6.6 (2.6,10.5)	31	15.4 (9.9,20.9)
Adjusted prevalence difference* (95% CI; q-value)	Referent		5.3 (1.8,8.8; 0.03)		14.0 (8.2,19.7; <0.001)	
An election worker, such as a poll worker or vote counter						
Not asked the question	953	27.4 (25.7,29.0)	25	8.5 (4.9,12.2)	13	5.9 (2.5,9.4)
Not willing	2417	70.0 (68.3,71.7)	252	74.5 (68.7,80.4)	151	63.0 (55.4,70.7)
Sometimes willing	39	1.4 (0.9,1.9)	28	10.3 (6.3,14.3)	21	11.7 (6.2,17.1)
Very or completely willing	20	0.7 (0.4,1.1)	13	6.0 (2.3,9.7)	30	17.9 (11.4,24.3)
Adjusted prevalence difference* (95% CI; q-value)	Referent		4.4 (1.1,7.8; 0.06)		15.9 (9.8,22.0; <0.001)	
A public health official						
Not asked the question	953	27.4 (25.7,29.0)	25	8.5 (4.9,12.2)	13	5.9 (2.5,9.4)
Not willing	2412	69.7 (68.0,71.4)	239	70.6 (64.5,76.6)	148	63.8 (56.3,71.3)
Sometimes willing	47	1.6 (1.1,2.2)	38	13.9 (9.2,18.6)	25	13.5 (8.0,19.1)
Very or completely willing	17	0.8 (0.4,1.2)	13	5.5 (2.0,9.0)	27	14.1 (8.4,19.8)
Adjusted prevalence difference* (95% CI; q-value)	Referent		3.9 (0.7,7.1; 0.16)		12.6 (6.9,18.3; <0.001)	
A member of the military or National Guard						
Not asked the quesion	953	27.4 (25.7,29.0)	25	8.5 (4.9,12.2)	13	5.9 (2.5,9.4)
Not willing	2359	67.9 (66.2,69.7)	244	71.6 (65.5,77.7)	147	62.5 (54.9,70.0)
Sometimes willing	93	3.1 (2.4,3.8)	31	10.3 (6.6,14.1)	24	13.0 (7.7,18.3)
Very or completely willing	25	1.1 (0.6,1.6)	16	8.4 (3.8,13.0)	32	17.6 (11.3,23.9)
Adjusted prevalence difference* (95% CI; q-value)	Referent		5.5 (1.5,9.5; 0.06)		13.5 (7.5,19.5; <0.001)	
A police officer						
Not asked the quesion	953	27.4 (25.7,29.0)	25	8.5 (4.9,12.2)	13	5.9 (2.5,9.4)
Not willing	2324	66.7 (64.9,68.4)	247	71.4 (65.3,77.6)	153	68.9 (61.9,76.0)
Sometimes willing	114	3.9 (3.1,4.6)	30	11.5 (7.1,15.9)	17	7.5 (3.7,11.4)
Very or completely willing	39	1.5 (1.0,2.1)	15	7.7 (3.4,11.9)	32	15.9 (10.2,21.5)
Adjusted prevalence difference* (95% CI; q-value)	Referent		4.5 (0.9,8.2; 0.11)		11.4 (5.8,16.9; 0.001)	
A person who does not share your race or ethnicity						
Not asked the quesion	953	27.4 (25.7,29.0)	25	8.5 (4.9,12.2)	13	5.9 (2.5,9.4)
Not willing	2424	69.9 (68.2,71.7)	246	72.0 (66.0,78.0)	149	62.0 (54.3,69.7)
Sometimes willing	38	1.4 (0.9,1.9)	29	11.2 (6.9,15.6)	21	12.4 (7.0,17.9)
Very or completely willing	17	0.8 (0.4,1.2)	16	7.1 (3.3,10.9)	30	17.7 (11.1,24.2)
Adjusted prevalence difference* (95% CI; q-value)	Referent		5.1 (1.7,8.6; 0.05)		14.7 (8.5,20.9; <0.001)	
A person who does not share your religion						
Not asked the quesion	953	27.4 (25.7,29.0)	25	8.5 (4.9,12.2)	13	5.9 (2.5,9.4)
Not willing	2420	69.7 (68.0,71.4)	253	74.9 (69.1,80.6)	154	65.8 (58.3,73.3)
Sometimes willing	37	1.5 (1.0,2.0)	27	10.5 (6.5,14.5)	19	11.7 (5.9,17.4)
Very or completely willing	19	0.8 (0.4,1.2)	13	5.5 (2.0,9.0)	28	14.9 (9.3,20.5)
Adjusted prevalence difference* (95% CI; q-value)	Referent		3.7 (0.5,6.9; 0.17)		12.0 (6.5,17.5; <0.001)	
A person who does not share your political beliefs						
Not asked the quesion	953	27.4 (25.7,29.0)	25	8.5 (4.9,12.2)	13	5.9 (2.5,9.4)
Not willing	2387	69.0 (67.2,70.7)	243	70.7 (64.5,76.8)	151	64.6 (57.0,72.1)
Sometimes willing	77	2.6 (2.0,3.3)	32	11.8 (7.3,16.2)	24	14.0 (8.1,19.9)
Very or completely willing	16	0.6 (0.3,0.9)	15	7.1 (3.2,11.1)	27	14.1 (8.5,19.8)
Adjusted prevalence difference* (95% CI; q-value)	Referent		5.9 (2.3,9.5; 0.02)		12.0 (6.7,17.3; <0.001)	

Table S10, continued.

In a situation where you think force or violence is justified to advance an important political objective…How willing would you personally be to use force or violence against a person because they are…	Three Percenters					
	Do Not Approve		Somewhat Approve		Strongly/Very Strongly Approve	
	Unweighted n	Weighted % (95% CI)	Unweighted n	Weighted % (95% CI)	Unweighted n	Weighted % (95% CI)
An elected federal or state government official						
Not asked the question	634	25.8 (23.8,27.7)	15	7.4 (3.6,11.3)	11	7.6 (2.7,12.6)
Not willing	1667	67.7 (65.6,69.9)	128	59.1 (51.1,67.0)	59	50.2 (40.0,60.4)
Sometimes willing	80	4.1 (3.1,5.1)	30	17.4 (11.2,23.7)	20	20.7 (11.8,29.7)
Very or completely willing	33	2.0 (1.2,2.8)	19	13.2 (6.9,19.5)	26	20.2 (12.2,28.1)
Adjusted prevalence difference* (95% CI; q-value)	Referent		9.6 (3.5,15.7; 0.03)		15.7 (7.6,23.9; 0.005)	
An elected local government official						
Not asked the question	634	25.8 (23.8,27.7)	15	7.4 (3.6,11.3)	11	7.6 (2.7,12.6)
Not willing	1681	68.7 (66.6,70.8)	130	62.1 (54.3,69.9)	56	45.4 (35.2,55.5)
Sometimes willing	66	3.5 (2.6,4.5)	37	21.3 (14.6,28.0)	21	22.1 (12.8,31.3)
Very or completely willing	28	1.4 (0.8,2.1)	13	8.5 (3.5,13.5)	27	22.0 (13.9,30.2)
Adjusted prevalence difference* (95% CI; q-value)	Referent		6.0 (1.2,10.8; 0.16)		19.5 (11.1,28.0; <0.001)	
An election worker, such as a poll worker or vote counter						
Not asked the question	634	25.8 (23.8,27.7)	15	7.4 (3.6,11.3)	11	7.6 (2.7,12.6)
Not willing	1721	70.9 (68.9,73.0)	138	65.5 (57.8,73.2)	55	42.6 (32.7,52.6)
Sometimes willing	33	1.6 (1.0,2.3)	25	15.4 (9.2,21.6)	23	21.4 (12.6,30.2)
Very or completely willing	20	1.1 (0.5,1.7)	17	11.0 (5.7,16.4)	26	26.1 (16.6,35.5)
Adjusted prevalence difference* (95% CI; q-value)	Referent		8.8 (3.4,14.2; 0.02)		23.3 (14.2,32.5; <0.001)	
A public health official						
Not asked the question	634	25.8 (23.8,27.7)	15	7.4 (3.6,11.3)	11	7.6 (2.7,12.6)
Not willing	1710	70.3 (68.3,72.4)	133	63.6 (55.8,71.4)	57	46.6 (36.4,56.8)
Sometimes willing	44	2.1 (1.4,2.7)	29	19.2 (12.2,26.1)	19	18.4 (10.1,26.6)
Very or completely willing	19	1.2 (0.6,1.8)	16	8.5 (4.1,12.9)	27	24.0 (15.1,33.0)
Adjusted prevalence difference* (95% CI; q-value)	Referent		6.5 (1.7,11.3; 0.11)		22.2 (13.2,31.3; <0.001)	
A member of the military or National Guard						
Not asked the quesion	634	25.8 (23.8,27.7)	15	7.4 (3.6,11.3)	11	7.6 (2.7,12.6)
Not willing	1680	68.8 (66.7,70.9)	135	65.6 (57.8,73.4)	53	43.3 (33.2,53.4)
Sometimes willing	67	3.1 (2.3,3.9)	22	11.5 (6.2,16.7)	28	25.2 (16.4,33.9)
Very or completely willing	29	1.9 (1.0,2.7)	22	14.4 (8.1,20.8)	23	21.5 (12.5,30.4)
Adjusted prevalence difference* (95% CI; q-value)	Referent		9.6 (3.7,15.6; 0.02)		16.9 (8.1,25.8; 0.005)	
A police officer						
Not asked the quesion	634	25.8 (23.8,27.7)	15	7.4 (3.6,11.3)	11	7.6 (2.7,12.6)
Not willing	1649	67.3 (65.2,69.4)	129	60.8 (52.8,68.7)	61	54.0 (43.8,64.1)
Sometimes willing	81	3.7 (2.8,4.5)	29	18.0 (11.3,24.7)	22	18.2 (10.6,25.9)
Very or completely willing	44	2.6 (1.7,3.4)	22	13.1 (7.3,18.9)	22	18.9 (10.9,26.9)
Adjusted prevalence difference* (95% CI; q-value)	Referent		7.9 (2.3,13.4; 0.06)		12.8 (4.6,21.0; 0.03)	
A person who does not share your race or ethnicity						
Not asked the quesion	634	25.8 (23.8,27.7)	15	7.4 (3.6,11.3)	11	7.6 (2.7,12.6)
Not willing	1726	70.9 (68.8,72.9)	134	63.3 (55.5,71.2)	58	44.0 (34.0,54.0)
Sometimes willing	29	1.3 (0.8,1.9)	32	21.1 (14.0,28.3)	22	22.2 (13.4,31.0)
Very or completely willing	21	1.4 (0.7,2.2)	12	6.2 (2.5,9.9)	26	25.5 (15.9,35.0)
Adjusted prevalence difference* (95% CI; q-value)	Referent		3.2 (-0.7,7.1; 0.53)		21.3 (12.1,30.6; <0.001)	
A person who does not share your religion						
Not asked the quesion	634	25.8 (23.8,27.7)	15	7.4 (3.6,11.3)	11	7.6 (2.7,12.6)
Not willing	1717	70.4 (68.3,72.4)	139	67.3 (59.7,74.8)	58	46.6 (36.5,56.8)
Sometimes willing	35	1.9 (1.2,2.6)	24	14.2 (8.4,20.1)	23	23.6 (14.2,33.0)
Very or completely willing	20	1.2 (0.6,1.9)	15	9.3 (4.4,14.2)	22	19.1 (11.1,27.0)
Adjusted prevalence difference* (95% CI; q-value)	Referent		7.1 (2.2,11.9; 0.03)		15.9 (7.8,24.0; 0.004)	
A person who does not share your political beliefs						
Not asked the quesion	634	25.8 (23.8,27.7)	15	7.4 (3.6,11.3)	11	7.6 (2.7,12.6)
Not willing	1701	70.0 (67.9,72.0)	137	64.9 (57.0,72.7)	52	43.1 (33.0,53.2)
Sometimes willing	60	2.7 (2.0,3.5)	29	18.9 (11.9,25.9)	24	22.9 (13.8,32.0)
Very or completely willing	17	1.1 (0.5,1.7)	12	6.7 (2.6,10.7)	29	25.1 (16.3,34.0)
Adjusted prevalence difference* (95% CI; q-value)	Referent		4.9 (0.7,9.1; 0.32)		23.3 (14.6,32.0; <0.001)	

Table S10, continued.

In a situation where you think force or violence is justified to advance an important political objective…How willing would you personally be to use force or violence against a person because they are…	QAnon					
	Do Not Approve		Somewhat Approve		Strongly/Very Strongly Approve	
	Unweighted n	Weighted % (95% CI)	Unweighted n	Weighted % (95% CI)	Unweighted n	Weighted % (95% CI)
An elected federal or state government official						
Not asked the question	1194	24.9 (23.6,26.3)	20	8.1 (4.4,11.8)	7	5.9 (1.1,10.7)
Not willing	3324	69.5 (68.1,71.0)	134	62.2 (54.7,69.6)	65	50.0 (39.4,60.6)
Sometimes willing	158	3.8 (3.2,4.5)	42	22.4 (15.9,28.8)	20	21.4 (12.1,30.7)
Very or completely willing	43	1.2 (0.8,1.6)	12	7.1 (2.8,11.5)	25	22.0 (12.7,31.2)
Adjusted prevalence difference* (95% CI; q-value)	Referent		5.2 (1.0,9.3; 0.10)		19.2 (10.2,28.2; <0.001)	
An elected local government official						
Not asked the question	1194	24.9 (23.6,26.3)	20	8.1 (4.4,11.8)	7	5.9 (1.1,10.7)
Not willing	3345	70.0 (68.6,71.5)	135	64.0 (56.7,71.3)	68	51.5 (40.8,62.2)
Sometimes willing	137	3.5 (2.8,4.1)	40	20.0 (13.9,26.0)	16	17.8 (8.8,26.9)
Very or completely willing	38	0.9 (0.6,1.3)	13	7.7 (3.2,12.2)	24	22.0 (13.0,31.0)
Adjusted prevalence difference* (95% CI; q-value)	Referent		6.2 (1.9,10.4; 0.05)		20.5 (11.5,29.5; <0.001)	
An election worker, such as a poll worker or vote counter						
Not asked the question	1194	24.9 (23.6,26.3)	20	8.1 (4.4,11.8)	7	5.9 (1.1,10.7)
Not willing	3432	72.3 (70.9,73.8)	154	72.8 (66.0,79.6)	65	47.2 (36.7,57.8)
Sometimes willing	58	1.4 (1.0,1.9)	29	15.5 (9.8,21.3)	18	17.4 (8.9,25.9)
Very or completely willing	30	0.8 (0.5,1.1)	6	3.6 (0.6,6.6)	26	27.7 (17.6,37.8)
Adjusted prevalence difference* (95% CI; q-value)	Referent		2.5 (-0.5,5.5; 0.33)		26.3 (16.5,36.1; <0.001)	
A public health official						
Not asked the question	1194	24.9 (23.6,26.3)	20	8.1 (4.4,11.8)	7	5.9 (1.1,10.7)
Not willing	3418	72.0 (70.6,73.5)	145	69.3 (62.5,76.2)	64	46.3 (35.8,56.8)
Sometimes willing	75	1.8 (1.3,2.2)	34	17.7 (11.9,23.6)	18	18.5 (9.7,27.2)
Very or completely willing	27	0.7 (0.4,1.0)	8	4.3 (1.2,7.3)	26	26.4 (16.4,36.4)
Adjusted prevalence difference* (95% CI; q-value)	Referent		3.0 (-0.1,6.0; 0.39)		25.4 (15.6,35.3; <0.001)	
A member of the military or National Guard						
Not asked the quesion	1194	24.9 (23.6,26.3)	20	8.1 (4.4,11.8)	7	5.9 (1.1,10.7)
Not willing	3348	70.2 (68.8,71.7)	142	66.3 (59.1,73.6)	68	50.3 (39.6,60.9)
Sometimes willing	136	3.3 (2.7,3.9)	31	15.2 (9.7,20.6)	17	16.2 (8.5,23.9)
Very or completely willing	38	1.1 (0.7,1.4)	15	9.7 (4.6,14.7)	25	26.9 (16.5,37.3)
Adjusted prevalence difference* (95% CI; q-value)	Referent		6.5 (2.0,11.0; 0.07)		23.8 (13.8,33.7; <0.001)	
A police officer						
Not asked the quesion	1194	24.9 (23.6,26.3)	20	8.1 (4.4,11.8)	7	5.9 (1.1,10.7)
Not willing	3291	68.6 (67.1,70.1)	145	67.2 (59.9,74.4)	70	57.0 (46.4,67.6)
Sometimes willing	174	4.3 (3.6,5.0)	30	15.5 (10.0,21.1)	15	14.4 (6.5,22.2)
Very or completely willing	57	1.6 (1.1,2.1)	14	9.2 (4.3,14.2)	25	22.0 (13.0,31.0)
Adjusted prevalence difference* (95% CI; q-value)	Referent		5.2 (0.8,9.5; 0.12)		18.1 (9.1,27.1; 0.001)	
A person who does not share your race or ethnicity						
Not asked the quesion	1194	24.9 (23.6,26.3)	20	8.1 (4.4,11.8)	7	5.9 (1.1,10.7)
Not willing	3436	72.3 (70.8,73.7)	154	72.1 (65.3,78.8)	64	45.2 (34.8,55.6)
Sometimes willing	57	1.5 (1.0,1.9)	25	13.5 (8.3,18.6)	17	19.1 (10.0,28.2)
Very or completely willing	27	0.7 (0.4,1.0)	10	6.4 (2.4,10.4)	29	29.1 (18.8,39.4)
Adjusted prevalence difference* (95% CI; q-value)	Referent		4.1 (0.4,7.7; 0.16)		27.3 (17.4,37.1; <0.001)	
A person who does not share your religion						
Not asked the quesion	1194	24.9 (23.6,26.3)	20	8.1 (4.4,11.8)	7	5.9 (1.1,10.7)
Not willing	3435	72.1 (70.7,73.6)	153	72.4 (65.7,79.1)	70	52.1 (41.5,62.8)
Sometimes willing	53	1.5 (1.0,1.9)	26	14.2 (8.8,19.6)	17	18.8 (9.6,28.0)
Very or completely willing	31	0.8 (0.5,1.1)	8	4.8 (1.4,8.2)	22	21.5 (12.4,30.6)
Adjusted prevalence difference* (95% CI; q-value)	Referent		2.4 (-0.6,5.4; 0.34)		19.7 (10.8,28.6; <0.001)	
A person who does not share your political beliefs						
Not asked the quesion	1194	24.9 (23.6,26.3)	20	8.1 (4.4,11.8)	7	5.9 (1.1,10.7)
Not willing	3381	71.1 (69.7,72.6)	150	70.6 (63.7,77.6)	68	49.7 (39.1,60.4)
Sometimes willing	117	2.8 (2.2,3.3)	28	15.0 (9.4,20.6)	17	20.9 (11.2,30.6)
Very or completely willing	26	0.7 (0.4,0.9)	8	4.8 (1.3,8.3)	25	22.8 (13.5,32.0)
Adjusted prevalence difference* (95% CI; q-value)	Referent		2.7 (-0.5,5.9; 0.41)		21.5 (12.6,30.4; <0.001)	

Table S10, continued.

In a situation where you think force or violence is justified to advance an important political objective…How willing would you personally be to use force or violence against a person because they are…	Christian Nationalist Movement					
	Do Not Approve		Somewhat Approve		Strongly/Very Strongly Approve	
	Unweighted n	Weighted % (95% CI)	Unweighted n	Weighted % (95% CI)	Unweighted n	Weighted % (95% CI)
**An elected federal or state government official**						
Not asked the question	884	26.3 (24.7,28.0)	64	11.2 (8.2,14.1)	26	9.1 (5.2,13.1)
Not willing	2229	67.3 (65.5,69.1)	432	72.4 (68.0,76.8)	253	65.5 (59.4,71.5)
Sometimes willing	120	4.4 (3.6,5.2)	57	11.9 (8.7,15.1)	36	11.5 (7.3,15.7)
Very or completely willing	35	1.4 (0.9,1.9)	17	4.4 (1.9,6.9)	36	12.7 (8.3,17.0)
Adjusted prevalence difference* (95% CI; q-value)	Referent		2.4 (-0.0,4.7; 0.25)		10.1 (6.0,14.3; <0.001)	
**An elected local government official**						
Not asked the question	884	26.3 (24.7,28.0)	64	11.2 (8.2,14.1)	26	9.1 (5.2,13.1)
Not willing	2250	68.1 (66.3,69.8)	430	72.8 (68.4,77.1)	262	69.2 (63.3,75.1)
Sometimes willing	102	3.8 (3.0,4.6)	53	10.8 (7.7,13.9)	32	10.2 (6.2,14.3)
Very or completely willing	31	1.2 (0.8,1.7)	19	4.7 (2.2,7.1)	34	11.2 (7.3,15.1)
Adjusted prevalence difference* (95% CI; q-value)	Referent		3.2 (0.9,5.6; 0.07)		9.1 (5.3,12.9; <0.001)	
**An election worker, such as a poll worker or vote counter**						
Not asked the question	884	26.3 (24.7,28.0)	64	11.2 (8.2,14.1)	26	9.1 (5.2,13.1)
Not willing	2319	70.8 (69.1,72.5)	452	75.5 (71.2,79.8)	268	70.2 (64.3,76.0)
Sometimes willing	39	1.4 (0.9,1.9)	41	9.3 (6.3,12.3)	29	9.5 (5.5,13.4)
Very or completely willing	25	1.0 (0.6,1.5)	12	3.7 (1.3,6.0)	31	10.8 (6.8,14.8)
Adjusted prevalence difference* (95% CI; q-value)	Referent		2.4 (0.2,4.6; 0.17)		9.0 (5.1,12.9; <0.001)	
**A public health official**						
Not asked the question	884	26.3 (24.7,28.0)	64	11.2 (8.2,14.1)	26	9.1 (5.2,13.1)
Not willing	2314	70.7 (69.0,72.4)	431	71.4 (66.9,75.9)	259	68.7 (62.8,74.5)
Sometimes willing	45	1.4 (0.9,1.8)	51	11.9 (8.4,15.3)	35	10.1 (6.4,13.9)
Very or completely willing	22	1.0 (0.5,1.4)	17	4.2 (1.9,6.6)	33	11.5 (7.4,15.6)
Adjusted prevalence difference* (95% CI; q-value)	Referent		2.9 (0.7,5.1; 0.10)		9.6 (5.6,13.6; <0.001)	
**A member of the military or National Guard**						
Not asked the quesion	884	26.3 (24.7,28.0)	64	11.2 (8.2,14.1)	26	9.1 (5.2,13.1)
Not willing	2235	67.9 (66.1,69.6)	441	73.9 (69.5,78.3)	269	69.0 (63.0,75.0)
Sometimes willing	117	4.1 (3.3,4.9)	41	7.8 (5.2,10.4)	25	8.5 (5.1,12.0)
Very or completely willing	30	1.1 (0.7,1.6)	22	6.6 (3.5,9.6)	33	12.9 (8.2,17.6)
Adjusted prevalence difference* (95% CI; q-value)	Referent		4.5 (1.7,7.4; 0.02)		9.8 (5.4,14.1; <0.001)	
**A police officer**						
Not asked the quesion	884	26.3 (24.7,28.0)	64	11.2 (8.2,14.1)	26	9.1 (5.2,13.1)
Not willing	2198	66.2 (64.4,68.0)	436	72.8 (68.4,77.2)	268	71.0 (65.2,76.7)
Sometimes willing	140	5.0 (4.1,5.9)	40	9.3 (6.2,12.4)	31	8.9 (5.6,12.2)
Very or completely willing	46	1.9 (1.3,2.5)	25	5.6 (3.1,8.1)	28	10.4 (6.3,14.5)
Adjusted prevalence difference* (95% CI; q-value)	Referent		2.9 (0.6,5.3; 0.11)		6.7 (2.8,10.5; 0.01)	
**A person who does not share your race or ethnicity**						
Not asked the quesion	884	26.3 (24.7,28.0)	64	11.2 (8.2,14.1)	26	9.1 (5.2,13.1)
Not willing	2326	70.8 (69.1,72.5)	450	75.4 (71.1,79.8)	264	68.6 (62.7,74.5)
Sometimes willing	38	1.4 (0.9,1.9)	43	9.8 (6.6,12.9)	24	7.8 (4.4,11.2)
Very or completely willing	20	0.9 (0.4,1.3)	12	3.4 (1.2,5.6)	37	13.3 (8.9,17.8)
Adjusted prevalence difference* (95% CI; q-value)	Referent		1.7 (-0.4,3.7; 0.34)		11.1 (6.8,15.4; <0.001)	
**A person who does not share your religion**						
Not asked the quesion	884	26.3 (24.7,28.0)	64	11.2 (8.2,14.1)	26	9.1 (5.2,13.1)
Not willing	2328	70.8 (69.1,72.5)	453	76.2 (72.0,80.4)	267	70.4 (64.7,76.2)
Sometimes willing	33	1.3 (0.8,1.8)	35	7.8 (5.0,10.5)	28	10.0 (5.8,14.1)
Very or completely willing	21	0.9 (0.4,1.3)	16	4.5 (2.0,7.0)	27	8.4 (5.2,11.7)
Adjusted prevalence difference* (95% CI; q-value)	Referent		3.2 (1.0,5.5; 0.04)		6.9 (3.5,10.3; 0.001)	
**A person who does not share your political beliefs**						
Not asked the quesion	884	26.3 (24.7,28.0)	64	11.2 (8.2,14.1)	26	9.1 (5.2,13.1)
Not willing	2280	69.4 (67.6,71.1)	441	74.2 (69.9,78.6)	272	71.4 (65.6,77.2)
Sometimes willing	86	3.0 (2.3,3.7)	48	10.2 (7.1,13.4)	27	9.5 (5.6,13.5)
Very or completely willing	19	0.7 (0.4,1.1)	12	3.3 (1.2,5.5)	29	9.6 (5.9,13.4)
Adjusted prevalence difference* (95% CI; q-value)	Referent		2.2 (0.2,4.2; 0.29)		8.1 (4.5,11.8; <0.001)	

Table S10, continued.

In a situation where you think force or violence is justified to advance an important political objective…How willing would you personally be to use force or violence against a person because they are…	White Supremacy Movement					
	Do Not Approve		Somewhat Approve		Strongly/Very Strongly Approve	
	Unweighted n	Weighted % (95% CI)	Unweighted n	Weighted % (95% CI)	Unweighted n	Weighted % (95% CI)
An elected federal or state government official						
Not asked the question	1542	22.5 (21.4,23.6)	6	5.1 (0.9,9.2)	5	7.3 (-0.3,14.9)
Not willing	4939	71.8 (70.6,73.0)	107	57.5 (48.4,66.6)	30	34.5 (22.5,46.5)
Sometimes willing	240	3.8 (3.3,4.4)	33	20.9 (13.7,28.0)	14	28.6 (15.5,41.8)
Very or completely willing	68	1.3 (0.9,1.6)	16	15.8 (7.8,23.7)	24	29.6 (18.2,41.0)
Adjusted prevalence difference* (95% CI; q-value)	Referent		13.9 (6.2,21.7; 0.006)		26.8 (15.4,38.3; <0.001)	
An elected local government official						
Not asked the question	1542	22.5 (21.4,23.6)	6	5.1 (0.9,9.2)	5	7.3 (-0.3,14.9)
Not willing	4980	72.5 (71.3,73.7)	100	53.6 (44.5,62.6)	29	33.1 (21.2,44.9)
Sometimes willing	203	3.3 (2.8,3.8)	42	27.6 (19.6,35.7)	14	24.6 (12.1,37.1)
Very or completely willing	60	1.0 (0.7,1.3)	15	13.8 (6.6,21.0)	25	35.0 (22.6,47.3)
Adjusted prevalence difference* (95% CI; q-value)	Referent		12.6 (5.5,19.6; 0.007)		33.2 (20.9,45.5; <0.001)	
An election worker, such as a poll worker or vote counter						
Not asked the question	1542	22.5 (21.4,23.6)	6	5.1 (0.9,9.2)	5	7.3 (-0.3,14.9)
Not willing	5107	74.5 (73.4,75.7)	112	61.5 (52.5,70.6)	34	40.7 (28.0,53.3)
Sometimes willing	96	1.6 (1.2,1.9)	26	17.0 (10.1,23.9)	16	27.2 (14.4,40.0)
Very or completely willing	45	0.8 (0.5,1.1)	19	16.4 (8.8,24.0)	18	24.8 (14.0,35.7)
Adjusted prevalence difference* (95% CI; q-value)	Referent		15.2 (7.7,22.7; 0.001)		23.1 (12.3,33.9; <0.001)	
A public health official						
Not asked the question	1542	22.5 (21.4,23.6)	6	5.1 (0.9,9.2)	5	7.3 (-0.3,14.9)
Not willing	5049	73.7 (72.5,74.9)	109	58.4 (49.3,67.5)	32	37.8 (25.3,50.2)
Sometimes willing	146	2.3 (1.9,2.7)	32	23.3 (15.3,31.3)	13	19.5 (9.1,30.0)
Very or completely willing	45	0.7 (0.5,1.0)	16	13.3 (6.4,20.2)	22	34.6 (21.6,47.6)
Adjusted prevalence difference* (95% CI; q-value)	Referent		12.3 (5.5,19.1; 0.005)		33.0 (20.3,45.7; <0.001)	
A member of the military or National Guard						
Not asked the quesion	1542	22.5 (21.4,23.6)	6	5.1 (0.9,9.2)	5	7.3 (-0.3,14.9)
Not willing	4987	72.6 (71.4,73.8)	108	58.6 (49.5,67.7)	32	36.3 (24.1,48.4)
Sometimes willing	201	3.3 (2.8,3.7)	29	18.3 (11.5,25.0)	14	20.9 (10.2,31.6)
Very or completely willing	58	1.1 (0.8,1.4)	18	16.8 (8.9,24.8)	22	35.5 (22.4,48.6)
Adjusted prevalence difference* (95% CI; q-value)	Referent		15.3 (7.5,23.1; 0.002)		32.6 (19.8,45.5; <0.001)	
A police officer						
Not asked the quesion	1542	22.5 (21.4,23.6)	6	5.1 (0.9,9.2)	5	7.3 (-0.3,14.9)
Not willing	4932	71.5 (70.3,72.7)	104	56.8 (47.7,65.9)	32	42.6 (29.4,55.7)
Sometimes willing	228	3.8 (3.2,4.3)	32	21.3 (13.7,28.9)	14	20.9 (10.2,31.5)
Very or completely willing	87	1.6 (1.2,2.0)	19	16.2 (8.7,23.8)	22	29.2 (17.6,40.8)
Adjusted prevalence difference* (95% CI; q-value)	Referent		13.7 (6.3,21.1; 0.004)		26.0 (14.4,37.6; <0.001)	
A person who does not share your race or ethnicity						
Not asked the quesion	1542	22.5 (21.4,23.6)	6	5.1 (0.9,9.2)	5	7.3 (-0.3,14.9)
Not willing	5109	74.4 (73.2,75.6)	103	56.8 (47.8,65.9)	30	36.4 (24.1,48.7)
Sometimes willing	102	1.8 (1.4,2.2)	35	24.8 (16.6,33.0)	14	19.2 (9.0,29.5)
Very or completely willing	37	0.7 (0.4,0.9)	17	12.6 (6.1,19.1)	24	37.1 (24.0,50.2)
Adjusted prevalence difference* (95% CI; q-value)	Referent		11.6 (5.2,18.1; 0.006)		35.4 (22.5,48.3; <0.001)	
A person who does not share your religion						
Not asked the quesion	1542	22.5 (21.4,23.6)	6	5.1 (0.9,9.2)	5	7.3 (-0.3,14.9)
Not willing	5107	74.4 (73.2,75.5)	116	65.7 (56.9,74.5)	32	38.4 (25.9,50.9)
Sometimes willing	89	1.6 (1.2,1.9)	23	15.4 (8.8,21.9)	15	25.6 (12.9,38.2)
Very or completely willing	44	0.8 (0.5,1.1)	14	11.7 (5.0,18.4)	20	28.0 (16.5,39.5)
Adjusted prevalence difference* (95% CI; q-value)	Referent		10.7 (4.0,17.4; 0.03)		26.2 (14.7,37.6; <0.001)	
A person who does not share your political beliefs						
Not asked the quesion	1542	22.5 (21.4,23.6)	6	5.1 (0.9,9.2)	5	7.3 (-0.3,14.9)
Not willing	5056	73.6 (72.5,74.8)	107	59.7 (50.7,68.8)	32	40.6 (27.9,53.4)
Sometimes willing	159	2.7 (2.2,3.1)	34	23.1 (15.0,31.3)	15	19.8 (9.4,30.1)
Very or completely willing	33	0.6 (0.4,0.8)	16	12.1 (5.8,18.4)	21	32.3 (19.6,45.0)
Adjusted prevalence difference* (95% CI; q-value)	Referent		11.5 (5.3,17.7; 0.004)		31.3 (18.7,43.8; <0.001)	

Table S10, continued.

In a situation where you think force or violence is justified to advance an important political objective…How willing would you personally be to use force or violence against a person because they are…	Militia Movement					
	Do Not Approve		Somewhat Approve		Strongly/Very Strongly Approve	
	Unweighted n	Weighted % (95% CI)	Unweighted n	Weighted % (95% CI)	Unweighted n	Weighted % (95% CI)
An elected federal or state government official						
Not asked the question	1081	25.2 (23.8,26.7)	23	6.7 (3.5,9.8)	9	4.9 (1.6,8.1)
Not willing	3039	69.9 (68.4,71.5)	299	73.6 (68.4,78.9)	71	46.7 (37.3,56.2)
Sometimes willing	130	3.4 (2.7,4.0)	50	13.8 (9.6,17.9)	25	21.2 (12.9,29.6)
Very or completely willing	33	1.1 (0.6,1.5)	16	4.9 (2.3,7.6)	33	24.8 (16.0,33.6)
Adjusted prevalence difference* (95% CI; q-value)	Referent		3.4 (0.7,6.2; 0.14)		23.0 (14.4,31.6; <0.001)	
An elected local government official						
Not asked the question	1081	25.2 (23.8,26.7)	23	6.7 (3.5,9.8)	9	4.9 (1.6,8.1)
Not willing	3053	70.1 (68.6,71.7)	301	74.3 (69.1,79.4)	72	49.7 (40.1,59.3)
Sometimes willing	107	3.0 (2.4,3.6)	51	13.5 (9.6,17.3)	25	19.1 (11.1,27.2)
Very or completely willing	34	1.0 (0.7,1.4)	12	4.0 (1.5,6.5)	33	24.6 (16.3,32.8)
Adjusted prevalence difference* (95% CI; q-value)	Referent		2.7 (0.1,5.2; 0.30)		22.8 (14.5,31.0; <0.001)	
An election worker, such as a poll worker or vote counter						
Not asked the question	1081	25.2 (23.8,26.7)	23	6.7 (3.5,9.8)	9	4.9 (1.6,8.1)
Not willing	3112	71.9 (70.3,73.4)	328	79.3 (74.1,84.5)	83	57.1 (47.6,66.7)
Sometimes willing	51	1.3 (0.9,1.7)	30	9.2 (5.4,12.9)	20	15.6 (8.1,23.2)
Very or completely willing	34	1.1 (0.7,1.5)	9	4.2 (1.2,7.2)	28	21.1 (13.2,29.0)
Adjusted prevalence difference* (95% CI; q-value)	Referent		2.6 (-0.3,5.5; 0.27)		19.0 (11.4,26.7; <0.001)	
A public health official						
Not asked the question	1081	25.2 (23.8,26.7)	23	6.7 (3.5,9.8)	9	4.9 (1.6,8.1)
Not willing	3098	71.6 (70.1,73.1)	310	76.4 (71.3,81.4)	78	51.8 (42.2,61.4)
Sometimes willing	70	1.8 (1.3,2.3)	42	11.4 (7.8,15.1)	22	16.4 (8.8,24.0)
Very or completely willing	26	0.8 (0.4,1.1)	11	3.6 (1.2,6.0)	29	24.8 (16.1,33.6)
Adjusted prevalence difference* (95% CI; q-value)	Referent		2.5 (0.1,5.0; 0.29)		23.7 (15.3,32.2; <0.001)	
A member of the military or National Guard						
Not asked the quesion	1081	25.2 (23.8,26.7)	23	6.7 (3.5,9.8)	9	4.9 (1.6,8.1)
Not willing	3046	70.1 (68.6,71.7)	317	77.8 (72.9,82.8)	78	49.9 (40.3,59.5)
Sometimes willing	114	2.9 (2.4,3.5)	38	10.3 (6.7,13.8)	18	13.3 (6.9,19.8)
Very or completely willing	36	1.2 (0.7,1.6)	11	4.1 (1.5,6.7)	34	30.2 (20.6,39.8)
Adjusted prevalence difference* (95% CI; q-value)	Referent		2.2 (-0.5,4.9; 0.42)		27.4 (18.2,36.5; <0.001)	
A police officer						
Not asked the quesion	1081	25.2 (23.8,26.7)	23	6.7 (3.5,9.8)	9	4.9 (1.6,8.1)
Not willing	3005	68.8 (67.3,70.4)	310	76.0 (70.9,81.1)	81	55.0 (45.4,64.6)
Sometimes willing	142	3.8 (3.1,4.5)	42	11.3 (7.7,14.9)	19	14.5 (7.4,21.7)
Very or completely willing	49	1.5 (1.0,2.0)	15	5.4 (2.4,8.4)	29	23.5 (15.0,32.0)
Adjusted prevalence difference* (95% CI; q-value)	Referent		3.1 (0.1,6.2; 0.24)		20.3 (11.8,28.7; <0.001)	
A person who does not share your race or ethnicity						
Not asked the quesion	1081	25.2 (23.8,26.7)	23	6.7 (3.5,9.8)	9	4.9 (1.6,8.1)
Not willing	3119	71.9 (70.3,73.4)	321	77.6 (72.4,82.9)	81	52.9 (43.3,62.6)
Sometimes willing	52	1.5 (1.1,1.9)	35	10.7 (6.8,14.7)	20	16.4 (8.7,24.0)
Very or completely willing	27	0.9 (0.5,1.3)	11	4.3 (1.4,7.1)	30	24.6 (15.9,33.3)
Adjusted prevalence difference* (95% CI; q-value)	Referent		2.8 (-0.1,5.6; 0.28)		22.5 (14.2,30.9; <0.001)	
A person who does not share your religion						
Not asked the quesion	1081	25.2 (23.8,26.7)	23	6.7 (3.5,9.8)	9	4.9 (1.6,8.1)
Not willing	3117	71.8 (70.3,73.3)	323	78.9 (73.8,83.9)	88	59.4 (49.9,68.9)
Sometimes willing	43	1.3 (0.8,1.7)	32	10.0 (6.2,13.7)	19	15.1 (7.6,22.5)
Very or completely willing	33	1.1 (0.7,1.5)	9	3.3 (0.8,5.7)	24	19.4 (11.7,27.2)
Adjusted prevalence difference* (95% CI; q-value)	Referent		1.6 (-0.9,4.1; 0.55)		17.2 (9.8,24.7; <0.001)	
A person who does not share your political beliefs						
Not asked the quesion	1081	25.2 (23.8,26.7)	23	6.7 (3.5,9.8)	9	4.9 (1.6,8.1)
Not willing	3087	71.1 (69.6,72.6)	310	76.1 (70.9,81.4)	81	56.0 (46.4,65.6)
Sometimes willing	83	2.3 (1.7,2.8)	46	13.6 (9.2,18.1)	21	15.8 (8.5,23.2)
Very or completely willing	27	0.8 (0.5,1.2)	8	2.1 (0.6,3.7)	28	21.6 (13.3,29.9)
Adjusted prevalence difference* (95% CI; q-value)	Referent		1.0 (-0.7,2.6; 0.58)		20.5 (12.5,28.4; <0.001)	

Table S10, continued.

In a situation where you think force or violence is justified to advance an important political objective…How willing would you personally be to use force or violence against a person because they are…	Boogaloo Movement					
	Do Not Approve		Somewhat Approve		Strongly/Very Strongly Approve	
	Unweighted n	Weighted % (95% CI)	Unweighted n	Weighted % (95% CI)	Unweighted n	Weighted % (95% CI)
An elected federal or state government official						
Not asked the question	661	24.8 (23.0,26.7)	13	11.9 (5.1,18.6)	1	1.1 (-1.0,3.2)
Not willing	1835	68.6 (66.6,70.6)	51	52.0 (40.4,63.5)	20	35.9 (21.8,49.9)
Sometimes willing	90	4.2 (3.3,5.2)	23	23.1 (13.8,32.5)	13	24.3 (12.0,36.6)
Very or completely willing	36	1.8 (1.1,2.5)	10	13.0 (4.2,21.9)	22	37.5 (23.3,51.6)
Adjusted prevalence difference* (95% CI; q-value)	Referent		8.9 (0.8,17.1; 0.29)		32.8 (19.2,46.4; <0.001)	
An elected local government official						
Not asked the question	661	24.8 (23.0,26.7)	13	11.9 (5.1,18.6)	1	1.1 (-1.0,3.2)
Not willing	1848	69.4 (67.4,71.3)	48	47.6 (36.1,59.1)	16	30.6 (16.6,44.6)
Sometimes willing	79	3.9 (2.9,4.8)	24	23.8 (14.3,33.3)	13	23.8 (11.3,36.4)
Very or completely willing	29	1.3 (0.8,1.8)	11	14.7 (5.5,24.0)	26	43.2 (28.9,57.4)
Adjusted prevalence difference* (95% CI; q-value)	Referent		12.8 (4.2,21.4; 0.05)		41.0 (26.8,55.2; <0.001)	
An election worker, such as a poll worker or vote counter						
Not asked the question	661	24.8 (23.0,26.7)	13	11.9 (5.1,18.6)	1	1.1 (-1.0,3.2)
Not willing	1897	71.6 (69.7,73.6)	54	51.9 (40.4,63.5)	18	34.5 (20.2,48.9)
Sometimes willing	31	1.5 (0.9,2.1)	20	19.9 (11.0,28.7)	15	29.1 (15.6,42.6)
Very or completely willing	29	1.4 (0.8,2.0)	10	16.3 (6.3,26.3)	22	34.0 (20.8,47.1)
Adjusted prevalence difference* (95% CI; q-value)	Referent		13.2 (4.1,22.4; 0.07)		30.3 (17.2,43.5; <0.001)	
A public health official						
Not asked the question	661	24.8 (23.0,26.7)	13	11.9 (5.1,18.6)	1	1.1 (-1.0,3.2)
Not willing	1888	71.4 (69.5,73.3)	52	49.9 (38.3,61.4)	20	37.7 (23.4,52.1)
Sometimes willing	50	2.2 (1.5,2.8)	19	20.1 (11.0,29.3)	15	30.7 (16.6,44.9)
Very or completely willing	21	1.1 (0.6,1.6)	12	16.2 (6.8,25.6)	19	28.2 (16.1,40.3)
Adjusted prevalence difference* (95% CI; q-value)	Referent		14.3 (5.6,23.1; 0.02)		26.1 (13.7,38.6; 0.001)	
A member of the military or National Guard						
Not asked the quesion	661	24.8 (23.0,26.7)	13	11.9 (5.1,18.6)	1	1.1 (-1.0,3.2)
Not willing	1834	68.9 (66.9,70.9)	53	52.7 (41.1,64.2)	17	30.1 (16.5,43.7)
Sometimes willing	87	3.7 (2.9,4.5)	20	20.1 (11.0,29.2)	17	29.6 (16.7,42.5)
Very or completely willing	36	1.9 (1.2,2.7)	11	15.4 (6.0,24.7)	21	37.9 (23.6,52.2)
Adjusted prevalence difference* (95% CI; q-value)	Referent		10.5 (1.7,19.4; 0.22)		31.9 (18.2,45.7; <0.001)	
A police officer						
Not asked the quesion	661	24.8 (23.0,26.7)	13	11.9 (5.1,18.6)	1	1.1 (-1.0,3.2)
Not willing	1801	67.3 (65.3,69.4)	51	50.6 (39.1,62.2)	21	38.4 (24.1,52.6)
Sometimes willing	111	4.9 (3.9,5.8)	22	21.5 (12.4,30.5)	15	29.2 (15.5,42.9)
Very or completely willing	47	2.4 (1.6,3.1)	11	16.1 (6.4,25.7)	19	30.1 (17.2,42.9)
Adjusted prevalence difference* (95% CI; q-value)	Referent		10.7 (1.7,19.7; 0.19)		23.6 (10.8,36.3; 0.009)	
A person who does not share your race or ethnicity						
Not asked the quesion	661	24.8 (23.0,26.7)	13	11.9 (5.1,18.6)	1	1.1 (-1.0,3.2)
Not willing	1894	71.2 (69.2,73.1)	53	52.0 (40.4,63.6)	17	32.2 (18.3,46.1)
Sometimes willing	38	1.7 (1.1,2.3)	23	24.5 (14.6,34.4)	17	34.1 (19.7,48.4)
Very or completely willing	27	1.7 (1.0,2.3)	8	11.7 (3.0,20.4)	21	31.4 (18.7,44.0)
Adjusted prevalence difference* (95% CI; q-value)	Referent		7.8 (-0.2,15.8; 0.41)		26.9 (14.0,39.8; 0.001)	
A person who does not share your religion						
Not asked the quesion	661	24.8 (23.0,26.7)	13	11.9 (5.1,18.6)	1	1.1 (-1.0,3.2)
Not willing	1893	71.1 (69.1,73.0)	49	49.5 (37.9,61.0)	20	40.5 (25.7,55.4)
Sometimes willing	40	2.1 (1.4,2.8)	22	21.0 (12.0,30.0)	11	19.4 (8.0,30.8)
Very or completely willing	24	1.3 (0.7,1.8)	11	16.4 (6.5,26.2)	22	34.5 (21.4,47.7)
Adjusted prevalence difference* (95% CI; q-value)	Referent		13.6 (4.6,22.7; 0.05)		32.6 (19.3,45.8; <0.001)	
A person who does not share your political beliefs						
Not asked the question	661	24.8 (23.0,26.7)	13	11.9 (5.1,18.6)	1	1.1 (-1.0,3.2)
Not willing	1863	70.1 (68.1,72.0)	50	51.0 (39.5,62.6)	20	37.8 (23.5,52.2)
Sometimes willing	75	3.4 (2.5,4.2)	23	23.5 (13.7,33.2)	14	28.5 (14.5,42.5)
Very or completely willing	22	1.1 (0.6,1.6)	11	13.6 (5.1,22.2)	21	31.3 (18.7,43.9)
Adjusted prevalence difference* (95% CI; q-value)	Referent		12.0 (4.4,19.6; 0.03)		29.8 (17.2,42.3; <0.001)	

* Prevalence differences are adjusted for age, race and ethnicity, gender, education, income, Census division, and rurality and are for the very or completely willing comparison. They are expressed in absolute percentage points. Q-values represent the probability that the given difference would be a false discovery; they represent the expected proportion of "false positives" that would be seen among the collection of all differences whose q-values were at or below the given q-value.

Table S11. Approval of individual organizations and movements and future likelihood of firearm possession and use in a situation where political violence is perceived as justified

Thinking now about the future and all the changes it might bring, how likely is it that you will use a gun in any of the following ways in the next few years—in a situation where you think force or violence is justified to advance an important political objective?	Proud Boys					
	Do Not Approve		Somewhat Approve		Strongly/Very Strongly Approve	
	Unweighted n	Weighted % (95% CI)	Unweighted n	Weighted % (95% CI)	Unweighted n	Weighted % (95% CI)
I will be armed with a gun.						
Not likely	4296	86.7 (85.6,87.8)	202	57.9 (51.6,64.2)	93	55.6 (46.6,64.5)
Somewhat likely	353	7.7 (6.9,8.6)	60	21.2 (15.8,26.6)	23	14.1 (8.2,20.0)
Very or extremely likely	210	5.0 (4.2,5.7)	55	18.8 (13.7,23.9)	44	28.9 (20.6,37.3)
Adjusted prevalence difference* (95% CI; q-value)	Referent		12.8 (7.6,17.9; <0.001)		21.7 (13.6,29.9; <0.001)	
I will carry a gun openly, so that people know I am armed.						
Not likely	4591	93.3 (92.4,94.1)	252	73.6 (67.7,79.5)	107	62.7 (53.9,71.5)
Somewhat likely	166	3.6 (3.0,4.2)	39	13.3 (8.9,17.7)	19	14.7 (7.7,21.7)
Very or extremely likely	98	2.5 (1.9,3.0)	27	11.2 (6.7,15.8)	34	21.2 (14.0,28.5)
Adjusted prevalence difference* (95% CI; q-value)	Referent		7.3 (2.9,11.8; 0.01)		16.3 (9.0,23.6; <0.001)	
I will threaten someone with a gun.						
Not likely	4803	98.0 (97.5,98.5)	297	89.5 (85.3,93.7)	136	79.4 (71.6,87.3)
Somewhat likely	32	0.8 (0.5,1.1)	14	5.4 (2.5,8.4)	8	7.4 (1.7,13.2)
Very or extremely likely	17	0.5 (0.2,0.7)	6	3.1 (0.4,5.8)	16	11.8 (6.1,17.4)
Adjusted prevalence difference* (95% CI; q-value)	Referent		2.0 (-0.6,4.7; 0.41)		10.5 (4.8,16.1; 0.008)	
I will shoot someone with a gun.						
Not likely	4752	96.9 (96.2,97.5)	288	86.3 (81.6,90.9)	131	78.0 (70.2,85.8)
Somewhat likely	73	1.8 (1.3,2.2)	19	6.5 (3.4,9.6)	19	14.3 (7.4,21.1)
Very or extremely likely	31	0.7 (0.4,1.1)	10	4.7 (1.6,7.8)	10	6.4 (2.3,10.6)
Adjusted prevalence difference* (95% CI; q-value)	Referent		3.4 (0.3,6.4; 0.17)		4.5 (0.5,8.6; 0.17)	

Table S11, continued.

Thinking now about the future and all the changes it might bring, how likely is it that you will use a gun in any of the following ways in the next few years—in a situation where you think force or violence is justified to advance an important political objective?	Oath Keepers					
	Do Not Approve		Somewhat Approve		Strongly/Very Strongly Approve	
	Unweighted n	Weighted % (95% CI)	Unweighted n	Weighted % (95% CI)	Unweighted n	Weighted % (95% CI)
I will be armed with a gun.						
Not likely	3072	87.6 (86.3,88.9)	216	63.5 (57.4,69.6)	122	51.8 (44.2,59.4)
Somewhat likely	228	7.2 (6.2,8.2)	51	18.4 (13.3,23.5)	30	14.4 (8.9,19.8)
Very or extremely likely	131	4.6 (3.7,5.4)	51	17.2 (12.5,21.9)	62	32.1 (24.9,39.3)
Adjusted prevalence difference* (95% CI; q-value)	Referent		11.3 (6.4,16.1; <0.001)		25.9 (18.7,33.2; <0.001)	
I will carry a gun openly, so that people know I am armed.						
Not likely	3273	94.1 (93.1,95.1)	254	76.6 (71.2,82.1)	141	61.3 (53.8,68.8)
Somewhat likely	97	3.0 (2.3,3.7)	33	10.8 (6.9,14.7)	29	15.5 (9.6,21.5)
Very or extremely likely	58	2.3 (1.6,2.9)	31	11.7 (7.4,15.9)	45	21.7 (15.5,27.9)
Adjusted prevalence difference* (95% CI; q-value)	Referent		7.3 (3.1,11.5; 0.006)		16.8 (10.5,23.1; <0.001)	
I will threaten someone with a gun.						
Not likely	3397	98.2 (97.6,98.8)	290	87.0 (82.3,91.6)	184	80.8 (74.2,87.4)
Somewhat likely	20	0.7 (0.3,1.0)	16	6.9 (3.4,10.5)	11	7.1 (2.3,11.8)
Very or extremely likely	9	0.4 (0.1,0.7)	11	5.0 (1.9,8.1)	20	10.7 (5.8,15.7)
Adjusted prevalence difference* (95% CI; q-value)	Referent		3.8 (0.8,6.7; 0.09)		9.2 (4.4,14.0; 0.005)	
I will shoot someone with a gun.						
Not likely	3363	97.0 (96.3,97.7)	284	84.9 (80.0,89.7)	176	77.7 (70.9,84.5)
Somewhat likely	48	1.8 (1.2,2.3)	22	8.2 (4.8,11.6)	20	11.5 (6.0,17.0)
Very or extremely likely	18	0.6 (0.3,0.9)	12	6.0 (2.4,9.6)	19	9.4 (4.8,13.9)
Adjusted prevalence difference* (95% CI; q-value)	Referent		4.5 (1.2,7.7; 0.07)		7.6 (3.2,12.0; 0.01)	

Table S11, continued.

Thinking now about the future and all the changes it might bring, how likely is it that you will use a gun in any of the following ways in the next few years—in a situation where you think force or violence is justified to advance an important political objective?	Three Percenters					
	Do Not Approve		Somewhat Approve		Strongly/Very Strongly Approve	
	Unweighted n	Weighted % (95% CI)	Unweighted n	Weighted % (95% CI)	Unweighted n	Weighted % (95% CI)
I will be armed with a gun.						
Not likely	2090	84.0 (82.3,85.8)	119	57.6 (49.7,65.5)	51	40.4 (30.4,50.4)
Somewhat likely	192	8.9 (7.6,10.3)	31	17.9 (11.3,24.5)	23	20.0 (11.9,28.2)
Very or extremely likely	128	6.5 (5.3,7.8)	40	22.0 (15.4,28.5)	43	37.9 (27.9,47.8)
Adjusted prevalence difference* (95% CI; q-value)	Referent		13.5 (6.6,20.4; 0.001)		28.7 (18.9,38.6; <0.001)	
I will carry a gun openly, so that people know I am armed.						
Not likely	2259	92.0 (90.7,93.4)	141	68.5 (61.0,76.1)	66	52.0 (41.7,62.2)
Somewhat likely	89	3.9 (3.0,4.7)	26	14.7 (8.9,20.6)	24	24.8 (15.5,34.2)
Very or extremely likely	62	3.6 (2.5,4.6)	25	14.9 (8.9,20.9)	27	21.5 (13.5,29.6)
Adjusted prevalence difference* (95% CI; q-value)	Referent		8.1 (2.3,14.0; 0.04)		13.9 (5.6,22.3; 0.02)	
I will threaten someone with a gun.						
Not likely	2375	97.6 (96.8,98.3)	171	84.1 (77.9,90.3)	86	68.5 (58.6,78.4)
Somewhat likely	20	1.1 (0.6,1.6)	12	8.4 (3.5,13.3)	9	10.7 (3.3,18.1)
Very or extremely likely	13	0.8 (0.3,1.3)	9	5.7 (1.7,9.6)	22	19.1 (11.2,27.1)
Adjusted prevalence difference* (95% CI; q-value)	Referent		4.0 (0.1,7.9; 0.33)		17.2 (9.4,25.0; <0.001)	
I will shoot someone with a gun.						
Not likely	2349	96.2 (95.2,97.2)	166	82.4 (76.2,88.7)	84	67.4 (57.5,77.3)
Somewhat likely	41	2.2 (1.5,3.0)	17	8.7 (4.5,13.0)	16	16.9 (8.5,25.2)
Very or extremely likely	19	1.0 (0.5,1.5)	9	7.0 (2.1,11.8)	17	14.1 (7.1,21.0)
Adjusted prevalence difference* (95% CI; q-value)	Referent		4.5 (0.3,8.8; 0.32)		11.6 (4.8,18.3; 0.02)	

Table S11, continued.

Thinking now about the future and all the changes it might bring, how likely is it that you will use a gun in any of the following ways in the next few years—in a situation where you think force or violence is justified to advance an important political objective?	QAnon					
	Do Not Approve		Somewhat Approve		Strongly/Very Strongly Approve	
	Unweighted n	Weighted % (95% CI)	Unweighted n	Weighted % (95% CI)	Unweighted n	Weighted % (95% CI)
I will be armed with a gun.						
Not likely	4129	85.4 (84.2,86.6)	129	61.5 (54.2,68.9)	67	52.0 (41.4,62.7)
Somewhat likely	360	8.3 (7.4,9.2)	31	12.9 (8.2,17.7)	23	22.1 (12.9,31.2)
Very or extremely likely	223	5.6 (4.8,6.4)	44	23.9 (17.3,30.4)	26	23.8 (14.7,32.9)
Adjusted prevalence difference* (95% CI; q-value)	Referent		16.8 (10.0,23.7; <0.001)		15.8 (6.7,24.9; 0.004)	
I will carry a gun openly, so that people know I am armed.						
Not likely	4444	92.8 (91.9,93.7)	154	70.3 (63.1,77.5)	71	57.4 (46.8,67.9)
Somewhat likely	164	3.8 (3.1,4.4)	24	12.7 (7.6,17.7)	19	19.8 (10.7,28.8)
Very or extremely likely	102	2.7 (2.1,3.4)	26	15.3 (9.3,21.3)	26	20.7 (12.7,28.8)
Adjusted prevalence difference* (95% CI; q-value)	Referent		10.4 (4.6,16.2; 0.004)		15.7 (7.3,24.0; 0.003)	
I will threaten someone with a gun.						
Not likely	4659	97.9 (97.4,98.4)	183	85.2 (79.4,91.0)	88	70.6 (60.6,80.6)
Somewhat likely	28	0.7 (0.4,0.9)	17	10.1 (5.1,15.2)	11	12.0 (4.3,19.7)
Very or extremely likely	18	0.6 (0.3,0.9)	5	3.2 (0.3,6.2)	18	15.7 (8.2,23.3)
Adjusted prevalence difference* (95% CI; q-value)	Referent		2.0 (-1.0,5.0; 0.52)		14.5 (7.0,22.0; 0.004)	
I will shoot someone with a gun.						
Not likely	4616	96.9 (96.3,97.5)	180	83.4 (77.3,89.5)	86	68.7 (58.5,78.8)
Somewhat likely	65	1.6 (1.2,2.1)	19	10.7 (5.8,15.6)	15	14.8 (6.7,22.9)
Very or extremely likely	30	0.8 (0.5,1.1)	6	4.5 (0.6,8.3)	15	13.3 (6.2,20.3)
Adjusted prevalence difference* (95% CI; q-value)	Referent		2.7 (-0.7,6.1; 0.37)		11.5 (4.4,18.6; 0.04)	

Table S11, continued.

Thinking now about the future and all the changes it might bring, how likely is it that you will use a gun in any of the following ways in the next few years—in a situation where you think force or violence is justified to advance an important political objective?	Christian Nationalist Movement					
	Do Not Approve		Somewhat Approve		Strongly/Very Strongly Approve	
	Unweighted n	Weighted % (95% CI)	Unweighted n	Weighted % (95% CI)	Unweighted n	Weighted % (95% CI)
I will be armed with a gun.						
Not likely	2900	87.0 (85.6,88.3)	399	67.1 (62.6,71.5)	232	62.6 (56.7,68.5)
Somewhat likely	226	7.2 (6.2,8.2)	93	17.8 (14.1,21.5)	57	16.4 (12.0,20.7)
Very or extremely likely	142	5.3 (4.3,6.3)	75	14.2 (10.9,17.5)	61	19.7 (14.6,24.7)
Adjusted prevalence difference* (95% CI; q-value)	Referent		8.0 (4.3,11.6; <0.001)		11.8 (6.6,17.0; <0.001)	
I will carry a gun openly, so that people know I am armed.						
Not likely	3093	93.4 (92.4,94.4)	464	79.9 (76.0,83.7)	274	73.1 (67.5,78.7)
Somewhat likely	108	3.4 (2.7,4.1)	59	11.7 (8.5,14.8)	35	11.0 (7.0,15.0)
Very or extremely likely	66	2.7 (1.9,3.4)	43	7.5 (5.1,9.8)	41	14.6 (9.9,19.2)
Adjusted prevalence difference* (95% CI; q-value)	Referent		3.6 (0.9,6.3; 0.05)		9.7 (5.1,14.4; <0.001)	
I will threaten someone with a gun.						
Not likely	3231	98.0 (97.4,98.6)	541	93.2 (90.6,95.7)	320	87.2 (82.7,91.8)
Somewhat likely	22	0.7 (0.4,1.1)	21	4.4 (2.5,6.4)	10	4.5 (1.2,7.7)
Very or extremely likely	11	0.5 (0.2,0.9)	6	1.7 (0.3,3.1)	20	7.3 (3.9,10.7)
Adjusted prevalence difference* (95% CI; q-value)	Referent		0.8 (-0.7,2.2; 0.71)		6.1 (2.7,9.5; 0.007)	
I will shoot someone with a gun.						
Not likely	3197	96.9 (96.2,97.6)	520	89.7 (86.7,92.6)	313	85.4 (80.8,90.1)
Somewhat likely	50	1.8 (1.2,2.3)	37	6.7 (4.5,9.0)	18	6.5 (3.0,9.9)
Very or extremely likely	22	0.8 (0.4,1.1)	9	2.3 (0.7,3.9)	20	7.2 (3.8,10.7)
Adjusted prevalence difference* (95% CI; q-value)	Referent		0.8 (-0.8,2.4; 0.58)		5.2 (1.9,8.6; 0.06)	

Table S11, continued.

Thinking now about the future and all the changes it might bring, how likely is it that you will use a gun in any of the following ways in the next few years—in a situation where you think force or violence is justified to advance an important political objective?	White Supremacy Movement					
	Do Not Approve		Somewhat Approve		Strongly/Very Strongly Approve	
	Unweighted n	Weighted % (95% CI)	Unweighted n	Weighted % (95% CI)	Unweighted n	Weighted % (95% CI)
I will be armed with a gun.						
Not likely	5772	82.6 (81.6,83.7)	94	52.5 (43.5,61.5)	32	42.8 (29.7,55.9)
Somewhat likely	619	9.9 (9.1,10.7)	35	25.5 (17.3,33.6)	14	18.0 (8.5,27.6)
Very or extremely likely	394	6.7 (6.0,7.4)	32	20.4 (13.3,27.5)	25	36.5 (23.6,49.4)
Adjusted prevalence difference* (95% CI; q-value)	Referent		10.5 (3.5,17.6; 0.01)		27.9 (15.0,40.7; <0.001)	
I will carry a gun openly, so that people know I am armed.						
Not likely	6311	91.3 (90.5,92.1)	114	66.9 (58.3,75.5)	34	43.9 (30.8,57.1)
Somewhat likely	297	4.8 (4.2,5.4)	25	16.3 (9.5,23.2)	13	21.7 (9.9,33.6)
Very or extremely likely	173	3.1 (2.5,3.6)	22	15.2 (8.6,21.8)	24	31.7 (19.9,43.4)
Adjusted prevalence difference* (95% CI; q-value)	Referent		9.6 (3.2,16.1; 0.02)		26.8 (14.7,38.9; <0.001)	
I will threaten someone with a gun.						
Not likely	6713	97.9 (97.5,98.4)	139	82.9 (75.9,89.9)	48	61.1 (47.9,74.2)
Somewhat likely	43	0.7 (0.5,1.0)	13	8.9 (3.6,14.2)	10	16.0 (5.2,26.8)
Very or extremely likely	21	0.4 (0.2,0.6)	9	6.6 (2.1,11.1)	14	22.4 (11.3,33.5)
Adjusted prevalence difference* (95% CI; q-value)	Referent		5.7 (1.2,10.3; 0.10)		21.3 (10.3,32.4; 0.005)	
I will shoot someone with a gun.						
Not likely	6627	96.6 (96.0,97.1)	132	77.6 (69.9,85.3)	47	59.6 (46.3,72.9)
Somewhat likely	117	2.0 (1.6,2.4)	19	13.1 (7.1,19.0)	12	19.9 (8.3,31.6)
Very or extremely likely	39	0.7 (0.4,0.9)	10	7.7 (2.5,13.0)	12	17.4 (7.5,27.2)
Adjusted prevalence difference* (95% CI; q-value)	Referent		6.5 (1.4,11.6; 0.09)		16.1 (6.2,26.0; 0.02)	

Table S11, continued.

Thinking now about the future and all the changes it might bring, how likely is it that you will use a gun in any of the following ways in the next few years—in a situation where you think force or violence is justified to advance an important political objective?	Militia Movement					
	Do Not Approve		Somewhat Approve		Strongly/Very Strongly Approve	
	Unweighted n	Weighted % (95% CI)	Unweighted n	Weighted % (95% CI)	Unweighted n	Weighted % (95% CI)
I will be armed with a gun.						
Not likely	3752	85.7 (84.5,87.0)	252	61.7 (56.1,67.4)	60	39.7 (30.4,49.0)
Somewhat likely	329	8.3 (7.3,9.2)	68	19.1 (14.4,23.7)	18	13.0 (6.4,19.6)
Very or extremely likely	196	5.4 (4.6,6.2)	66	17.3 (12.9,21.7)	62	45.3 (35.8,54.9)
Adjusted prevalence difference* (95% CI; q-value)	Referent		10.4 (5.9,14.9; <0.001)		37.9 (28.5,47.3; <0.001)	
I will carry a gun openly, so that people know I am armed.						
Not likely	4034	92.8 (91.8,93.8)	311	76.8 (71.6,81.9)	79	53.6 (44.1,63.2)
Somewhat likely	150	3.8 (3.1,4.5)	47	12.7 (8.8,16.6)	22	16.2 (8.8,23.5)
Very or extremely likely	92	2.8 (2.1,3.4)	28	8.7 (5.0,12.4)	39	28.2 (19.7,36.8)
Adjusted prevalence difference* (95% CI; q-value)	Referent		4.1 (0.5,7.7; 0.13)		22.6 (14.0,31.2; <0.001)	
I will threaten someone with a gun.						
Not likely	4227	97.8 (97.2,98.4)	369	91.9 (88.4,95.4)	109	74.1 (65.6,82.6)
Somewhat likely	27	0.8 (0.4,1.1)	15	5.0 (2.3,7.7)	14	11.5 (4.8,18.2)
Very or extremely likely	17	0.7 (0.3,1.0)	3	1.4 (-0.4,3.2)	18	13.5 (7.2,19.8)
Adjusted prevalence difference* (95% CI; q-value)	Referent		0.0 (-1.8,1.8; 1.00)		11.7 (5.5,17.9; 0.006)	
I will shoot someone with a gun.						
Not likely	4191	96.8 (96.2,97.5)	358	89.4 (85.7,93.2)	100	68.9 (60.0,77.8)
Somewhat likely	58	1.6 (1.1,2.1)	22	6.3 (3.4,9.1)	24	18.7 (10.9,26.5)
Very or extremely likely	28	1.0 (0.6,1.4)	7	2.6 (0.4,4.8)	16	10.2 (4.9,15.5)
Adjusted prevalence difference* (95% CI; q-value)	Referent		1.1 (-1.1,3.3; 0.63)		7.9 (2.5,13.2; 0.08)	

Table S11, continued.

Thinking now about the future and all the changes it might bring, how likely is it that you will use a gun in any of the following ways in the next few years—in a situation where you think force or violence is justified to advance an important political objective?	Boogaloo Movement					
	Do Not Approve		Somewhat Approve		Strongly/Very Strongly Approve	
	Unweighted n	Weighted % (95% CI)	Unweighted n	Weighted % (95% CI)	Unweighted n	Weighted % (95% CI)
I will be armed with a gun.						
Not likely	2275	84.1 (82.4,85.8)	52	50.7 (39.1,62.2)	23	43.2 (28.6,57.8)
Somewhat likely	214	9.1 (7.9,10.4)	16	16.7 (8.1,25.4)	11	19.4 (7.4,31.3)
Very or extremely likely	135	6.3 (5.1,7.5)	26	29.0 (18.8,39.2)	23	37.5 (23.8,51.1)
Adjusted prevalence difference* (95% CI; q-value)	Referent		19.2 (8.5,29.9; 0.004)		27.0 (13.2,40.9; 0.002)	
I will carry a gun openly, so that people know I am armed.						
Not likely	2453	91.6 (90.2,92.9)	65	62.3 (51.0,73.5)	25	48.1 (33.5,62.8)
Somewhat likely	100	4.2 (3.3,5.1)	15	19.9 (10.2,29.5)	11	17.2 (7.1,27.3)
Very or extremely likely	71	3.8 (2.7,4.8)	14	14.3 (6.6,21.9)	21	34.7 (21.2,48.2)
Adjusted prevalence difference* (95% CI; q-value)	Referent		6.4 (-1.8,14.6; 0.33)		25.6 (12.4,38.7; 0.004)	
I will threaten someone with a gun.						
Not likely	2578	97.1 (96.2,97.9)	78	77.2 (67.5,86.9)	32	56.1 (41.8,70.4)
Somewhat likely	23	1.1 (0.6,1.6)	9	11.0 (3.8,18.2)	10	17.7 (6.6,28.7)
Very or extremely likely	17	1.1 (0.5,1.7)	7	8.2 (1.9,14.5)	15	26.2 (13.9,38.5)
Adjusted prevalence difference* (95% CI; q-value)	Referent		5.3 (-1.0,11.6; 0.55)		22.6 (10.4,34.8; 0.008)	
I will shoot someone with a gun.						
Not likely	2546	95.7 (94.8,96.7)	75	75.1 (65.2,84.9)	32	57.0 (42.8,71.3)
Somewhat likely	47	2.1 (1.4,2.8)	13	14.6 (6.7,22.5)	11	17.8 (7.5,28.2)
Very or extremely likely	29	1.5 (0.9,2.2)	6	6.8 (1.1,12.4)	14	25.1 (12.6,37.6)
Adjusted prevalence difference* (95% CI; q-value)	Referent		3.5 (-2.2,9.2; 0.66)		20.3 (8.6,32.0; 0.02)	

* Prevalence differences are adjusted for age, race and ethnicity, gender, education, income, Census division, and rurality and are for the very or extremely likely comparison. They are expressed in absolute percentage points. Q-values represent the probability that the given difference would be a false discovery; they represent the expected proportion of "false positives" that would be seen among the collection of all differences whose q-values were at or below the given q-value.

Table S12. Approval of individual organizations and movements and beliefs concerning democracy in the US

Statement	Proud Boys					
	Do Not Approve		Somewhat Approve		Strongly/Very Strongly Approve	
	Unweighted n	Weighted % (95% CI)	Unweighted n	Weighted % (95% CI)	Unweighted n	Weighted % (95% CI)
Do you believe that things in this country today are…						
Generally headed in the wrong direction	3840	78.6 (77.3,79.9)	283	83.0 (77.5,88.5)	130	74.8 (66.3,83.3)
Generally headed in the right direction	1012	20.8 (19.5,22.1)	39	16.3 (10.8,21.7)	31	25.2 (16.7,33.7)
Adjusted prevalence difference* (95% CI; q-value)	Referent		5.9 (0.5,11.2; 0.08)		-1.3 (-9.3,6.8; 0.80)	
When thinking about democracy in the United States these days, do you believe…						
There is a serious threat to our democracy.	3689	73.0 (71.6,74.5)	217	61.8 (55.5,68.0)	111	61.3 (52.2,70.4)
There may be a threat to our democracy, but it is not serious.	925	20.8 (19.5,22.2)	75	26.4 (20.6,32.1)	28	19.8 (12.1,27.5)
There is no threat to our democracy.	241	5.4 (4.7,6.2)	31	11.5 (7.1,15.9)	22	18.9 (11.2,26.6)
Adjusted prevalence difference* (95% CI; q-value)	Referent		-8.8 (-15.0,-2.6; 0.02)		-5.1 (-13.4,3.1; 0.38)	
How important do you think it is for the United States to remain a democracy?						
Not important	37	0.9 (0.6,1.3)	19	6.5 (3.5,9.5)	8	7.0 (2.1,12.0)
Somewhat important	165	4.5 (3.8,5.2)	41	16.6 (11.3,22.0)	16	10.8 (4.7,17.0)
Very or extremely important	4663	94.1 (93.2,94.9)	264	76.8 (71.1,82.6)	137	82.1 (74.7,89.6)
Adjusted prevalence difference* (95% CI; q-value)	Referent		4.9 (1.9,7.8; 0.01)		4.6 (0.0,9.2; 0.24)	
Democracy is the best form of government.						
Do not agree	142	3.6 (2.9,4.2)	35	11.6 (7.6,15.6)	23	15.3 (8.6,22.0)
Somewhat agree	835	19.9 (18.5,21.2)	87	31.1 (25.0,37.2)	27	19.4 (12.0,26.7)
Strongly or very strongly agree	3883	76.0 (74.6,77.4)	199	56.1 (49.8,62.4)	111	65.3 (56.6,74.0)
Adjusted prevalence difference* (95% CI; q-value)	Referent		6.5 (2.3,10.6; 0.02)		8.5 (2.2,14.7; 0.06)	
These days, American democracy only serves the interests of the wealthy and powerful.						
Do not agree	1431	25.7 (24.4,27.0)	93	23.9 (19.0,28.8)	36	23.4 (15.6,31.1)
Somewhat agree	1749	36.2 (34.7,37.7)	113	38.3 (32.0,44.5)	36	25.4 (17.1,33.6)
Strongly or very strongly agree	1689	37.8 (36.3,39.4)	116	36.9 (30.8,43.0)	89	51.3 (42.3,60.2)
Adjusted prevalence difference* (95% CI; q-value)	Referent		-3.2 (-9.8,3.4; 0.49)		6.2 (-3.4,15.7; 0.35)	
Having a strong leader for America is more important than having a democracy.						
Do not agree	3424	67.5 (66.0,69.0)	134	35.5 (29.9,41.2)	68	38.8 (30.2,47.3)
Somewhat agree	808	18.2 (16.9,19.4)	93	30.8 (25.0,36.7)	24	16.1 (9.5,22.7)
Strongly or very strongly agree	634	13.9 (12.8,15.0)	94	32.4 (26.3,38.6)	69	45.2 (36.2,54.1)
Adjusted prevalence difference* (95% CI; q-value)	Referent		15.6 (9.5,21.7; <0.001)		26.8 (18.0,35.7; <0.001)	
The 2020 election was stolen from Donald Trump, and Joe Biden is an illegitimate president.						
Do not agree	4187	86.2 (85.1,87.2)	69	21.7 (16.6,26.7)	26	16.6 (9.7,23.6)
Somewhat agree	333	6.6 (5.8,7.3)	97	32.3 (26.2,38.4)	30	21.7 (14.1,29.3)
Strongly or very strongly agree	341	6.9 (6.1,7.7)	155	45.4 (39.2,51.7)	104	60.6 (51.7,69.5)
Adjusted prevalence difference* (95% CI; q-value)	Referent		36.4 (30.4,42.5; <0.001)		53.3 (44.7,61.9; <0.001)	
Armed citizens should patrol polling places at election time.						
Do not agree	4545	92.1 (91.2,93.1)	197	56.3 (50.0,62.7)	60	33.4 (25.2,41.6)
Somewhat agree	206	4.7 (4.0,5.4)	82	28.2 (22.3,34.0)	34	21.7 (14.3,29.0)
Strongly or very strongly agree	115	2.8 (2.2,3.4)	44	15.2 (10.4,20.0)	67	44.9 (36.0,53.9)
Adjusted prevalence difference* (95% CI; q-value)	Referent		10.5 (5.8,15.2; <0.001)		39.1 (30.2,48.0; <0.001)	
In the next few years, there will be civil war in the United States						
Do not agree	2712	53.8 (52.3,55.4)	96	28.9 (23.2,34.6)	33	21.1 (13.6,28.6)
Somewhat agree	1659	34.4 (32.9,35.9)	155	45.4 (39.2,51.7)	54	31.1 (23.1,39.2)
Strongly or very strongly agree	473	11.0 (9.9,12.1)	70	24.5 (18.9,30.1)	74	47.8 (38.8,56.7)
Adjusted prevalence difference* (95% CI; q-value)	Referent		10.9 (5.1,16.6; 0.001)		32.1 (23.0,41.1; <0.001)	

Table S12, continued.

Statement	Oath Keepers					
	Do Not Approve		Somewhat Approve		Strongly/Very Strongly Approve	
	Unweighted n	Weighted % (95% CI)	Unweighted n	Weighted % (95% CI)	Unweighted n	Weighted % (95% CI)
Do you believe that things in this country today are…						
Generally headed in the wrong direction	2616	75.6 (73.9,77.2)	270	80.2 (74.6,85.9)	170	72.4 (64.9,79.8)
Generally headed in the right direction	811	23.9 (22.3,25.5)	47	18.7 (13.2,24.3)	47	26.4 (19.1,33.7)
Adjusted prevalence difference* (95% CI; q-value)	Referent		6.9 (1.3,12.5; 0.09)		-0.5 (-7.6,6.5; 0.93)	
When thinking about democracy in the United States these days, do you believe…						
There is a serious threat to our democracy.	2732	77.3 (75.7,79.0)	208	57.7 (51.3,64.1)	153	63.3 (55.6,71.0)
There may be a threat to our democracy, but it is not serious.	539	16.6 (15.1,18.0)	77	29.5 (23.3,35.6)	36	19.4 (12.8,26.1)
There is no threat to our democracy.	161	5.6 (4.7,6.6)	35	12.4 (8.0,16.7)	29	17.3 (11.1,23.4)
Adjusted prevalence difference* (95% CI; q-value)	Referent		-16.1 (-22.3,-9.9; <0.001)		-7.1 (-14.3,0.2; 0.16)	
How important do you think it is for the United States to remain a democracy?						
Not important	31	1.2 (0.7,1.6)	14	4.4 (2.0,6.8)	13	8.2 (3.7,12.7)
Somewhat important	90	3.8 (2.9,4.7)	45	19.0 (13.5,24.6)	21	11.9 (6.6,17.2)
Very or extremely important	3318	94.7 (93.7,95.7)	262	76.6 (70.8,82.4)	184	79.9 (73.4,86.4)
Adjusted prevalence difference* (95% CI; q-value)	Referent		2.1 (-0.4,4.7; 0.28)		5.0 (0.7,9.3; 0.15)	
Democracy is the best form of government.						
Do not agree	98	3.5 (2.8,4.3)	26	8.7 (4.8,12.5)	27	13.2 (8.0,18.4)
Somewhat agree	494	16.3 (14.9,17.8)	75	28.4 (22.4,34.3)	51	26.8 (19.8,33.7)
Strongly or very strongly agree	2845	79.8 (78.2,81.4)	217	61.5 (55.1,67.8)	139	59.8 (52.2,67.3)
Adjusted prevalence difference* (95% CI; q-value)	Referent		3.1 (-0.9,7.0; 0.38)		6.2 (1.2,11.2; 0.09)	
These days, American democracy only serves the interests of the wealthy and powerful.						
Do not agree	1008	25.9 (24.4,27.5)	96	24.1 (19.2,29.1)	59	24.5 (18.2,30.8)
Somewhat agree	1261	37.2 (35.4,39.0)	114	38.8 (32.5,45.1)	46	25.2 (18.1,32.2)
Strongly or very strongly agree	1170	36.7 (34.9,38.5)	108	35.8 (29.8,41.9)	111	49.7 (42.0,57.3)
Adjusted prevalence difference* (95% CI; q-value)	Referent		-3.5 (-10.0,2.9; 0.52)		5.6 (-2.8,14.1; 0.42)	
Having a strong leader for America is more important than having a democracy.						
Do not agree	2558	71.4 (69.6,73.1)	154	42.6 (36.4,48.8)	80	32.2 (25.4,39.0)
Somewhat agree	465	14.7 (13.3,16.0)	91	29.6 (23.9,35.3)	43	21.2 (14.8,27.6)
Strongly or very strongly agree	412	13.5 (12.1,14.9)	73	26.3 (20.4,32.2)	92	45.3 (37.7,53.0)
Adjusted prevalence difference* (95% CI; q-value)	Referent		7.5 (1.7,13.2; 0.03)		25.9 (18.2,33.6; <0.001)	
The 2020 election was stolen from Donald Trump, and Joe Biden is an illegitimate president.						
Do not agree	3089	89.0 (87.8,90.2)	101	32.2 (26.2,38.2)	49	22.0 (15.9,28.0)
Somewhat agree	166	5.1 (4.3,6.0)	102	31.5 (25.7,37.3)	39	22.7 (15.8,29.6)
Strongly or very strongly agree	183	5.6 (4.7,6.5)	117	36.1 (30.1,42.1)	128	54.3 (46.7,62.0)
Adjusted prevalence difference* (95% CI; q-value)	Referent		27.2 (21.4,32.9; <0.001)		47.9 (40.4,55.4; <0.001)	
Armed citizens should patrol polling places at election time.						
Do not agree	3248	93.2 (92.1,94.2)	203	57.5 (51.1,63.8)	87	36.9 (29.7,44.1)
Somewhat agree	114	3.7 (3.0,4.5)	70	24.8 (19.1,30.4)	53	25.3 (18.5,32.1)
Strongly or very strongly agree	72	2.7 (2.0,3.4)	46	17.2 (12.1,22.3)	76	37.2 (29.7,44.7)
Adjusted prevalence difference* (95% CI; q-value)	Referent		11.8 (6.9,16.7; <0.001)		31.0 (23.6,38.5; <0.001)	
In the next few years, there will be civil war in the United States						
Do not agree	1956	55.3 (53.4,57.1)	115	33.1 (27.2,39.0)	44	20.3 (14.2,26.5)
Somewhat agree	1171	34.1 (32.3,35.8)	142	43.1 (37.0,49.3)	74	33.4 (26.2,40.6)
Strongly or very strongly agree	299	10.1 (8.9,11.3)	61	22.4 (16.9,28.0)	98	45.6 (38.0,53.2)
Adjusted prevalence difference* (95% CI; q-value)	Referent		8.4 (2.9,13.9; 0.01)		31.2 (23.3,39.1; <0.001)	

Table S12, continued.

Statement	Three Percenters					
	Do Not Approve		Somewhat Approve		Strongly/Very Strongly Approve	
	Unweighted n	Weighted % (95% CI)	Unweighted n	Weighted % (95% CI)	Unweighted n	Weighted % (95% CI)
Do you believe that things in this country today are…						
Generally headed in the wrong direction	1871	76.8 (74.8,78.7)	157	74.9 (67.3,82.5)	79	62.1 (51.7,72.4)
Generally headed in the right direction	539	22.8 (20.9,24.7)	39	24.2 (16.7,31.7)	38	37.6 (27.2,48.0)
Adjusted prevalence difference* (95% CI; q-value)	Referent		-0.1 (-7.5,7.2; 0.97)		-13.0 (-22.9,-3.0; 0.04)	
When thinking about democracy in the United States these days, do you believe…						
There is a serious threat to our democracy.	1916	76.4 (74.4,78.4)	113	51.9 (44.0,59.9)	68	49.0 (38.8,59.2)
There may be a threat to our democracy, but it is not serious.	366	16.5 (14.7,18.2)	59	33.9 (26.3,41.5)	27	27.2 (17.4,36.9)
There is no threat to our democracy.	133	6.7 (5.4,8.0)	24	13.5 (7.8,19.2)	23	23.9 (14.8,32.9)
Adjusted prevalence difference* (95% CI; q-value)	Referent		-19.4 (-27.2,-11.5; <0.001)		-17.2 (-26.5,-7.8; 0.002)	
How important do you think it is for the United States to remain a democracy?						
Not important	34	1.8 (1.1,2.4)	8	3.9 (1.0,6.8)	10	9.6 (3.4,15.7)
Somewhat important	86	5.1 (3.9,6.3)	38	22.7 (15.6,29.8)	17	17.6 (9.5,25.8)
Very or extremely important	2297	92.9 (91.5,94.2)	151	73.4 (66.1,80.8)	91	72.8 (63.4,82.2)
Adjusted prevalence difference* (95% CI; q-value)	Referent		0.7 (-2.2,3.5; 0.83)		5.4 (-0.7,11.5; 0.34)	
Democracy is the best form of government.						
Do not agree	97	5.1 (4.0,6.2)	16	7.4 (3.5,11.3)	15	13.6 (6.4,20.7)
Somewhat agree	384	18.8 (16.9,20.6)	56	32.6 (24.9,40.2)	25	23.5 (14.6,32.5)
Strongly or very strongly agree	1933	75.8 (73.8,77.9)	124	59.2 (51.4,67.1)	76	60.9 (50.8,71.0)
Adjusted prevalence difference* (95% CI; q-value)	Referent		-1.2 (-5.9,3.5; 0.83)		3.6 (-2.9,10.0; 0.59)	
These days, American democracy only serves the interests of the wealthy and powerful.						
Do not agree	668	24.7 (22.8,26.5)	38	17.2 (11.7,22.7)	22	16.3 (8.9,23.6)
Somewhat agree	865	36.1 (33.9,38.2)	83	44.9 (37.0,52.8)	34	32.5 (22.6,42.5)
Strongly or very strongly agree	884	39.0 (36.8,41.2)	75	37.1 (29.5,44.8)	61	50.2 (39.9,60.4)
Adjusted prevalence difference* (95% CI; q-value)	Referent		-6.3 (-14.5,2.0; 0.33)		4.6 (-6.4,15.6; 0.66)	
Having a strong leader for America is more important than having a democracy.						
Do not agree	1687	66.0 (63.8,68.2)	66	30.3 (23.3,37.3)	45	34.4 (25.0,43.9)
Somewhat agree	381	17.2 (15.5,19.0)	63	34.1 (26.6,41.6)	19	19.9 (11.3,28.5)
Strongly or very strongly agree	345	16.4 (14.6,18.2)	66	34.1 (26.4,41.7)	52	43.7 (33.5,53.8)
Adjusted prevalence difference* (95% CI; q-value)	Referent		13.2 (5.3,21.1; 0.01)		21.9 (11.5,32.3; 0.001)	
The 2020 election was stolen from Donald Trump, and Joe Biden is an illegitimate president.						
Do not agree	2050	83.9 (82.2,85.6)	65	31.9 (24.7,39.1)	25	20.5 (12.3,28.6)
Somewhat agree	158	6.9 (5.7,8.0)	59	30.3 (22.9,37.6)	22	24.5 (15.1,33.9)
Strongly or very strongly agree	207	8.9 (7.6,10.3)	71	36.6 (29.0,44.3)	71	55.0 (44.8,65.3)
Adjusted prevalence difference* (95% CI; q-value)	Referent		25.6 (18.2,33.0; <0.001)		44.2 (34.1,54.3; <0.001)	
Armed citizens should patrol polling places at election time.						
Do not agree	2218	90.2 (88.8,91.7)	103	46.3 (38.5,54.1)	35	26.5 (17.7,35.4)
Somewhat agree	114	5.2 (4.1,6.2)	52	28.9 (21.5,36.3)	32	28.8 (19.5,38.2)
Strongly or very strongly agree	80	4.2 (3.1,5.3)	42	24.8 (17.6,32.0)	49	43.2 (33.0,53.3)
Adjusted prevalence difference* (95% CI; q-value)	Referent		16.9 (9.9,23.9; <0.001)		34.7 (24.4,44.9; <0.001)	
In the next few years, there will be civil war in the United States						
Do not agree	1290	51.7 (49.5,54.0)	53	26.7 (19.7,33.7)	16	13.3 (6.3,20.2)
Somewhat agree	850	34.8 (32.7,37.0)	92	46.9 (39.0,54.7)	34	27.9 (18.8,37.1)
Strongly or very strongly agree	269	13.0 (11.4,14.7)	51	25.7 (18.8,32.5)	67	57.7 (47.6,67.8)
Adjusted prevalence difference* (95% CI; q-value)	Referent		7.4 (0.0,14.8; 0.12)		39.4 (29.0,49.7; <0.001)	

Table S12, continued.

Statement	QAnon					
	Do Not Approve		Somewhat Approve		Strongly/Very Strongly Approve	
	Unweighted n	Weighted % (95% CI)	Unweighted n	Weighted % (95% CI)	Unweighted n	Weighted % (95% CI)
Do you believe that things in this country today are…						
Generally headed in the wrong direction	3753	79.1 (77.8,80.4)	177	81.6 (75.0,88.2)	83	60.9 (50.0,71.9)
Generally headed in the right direction	957	20.3 (19.0,21.6)	30	17.4 (10.8,23.9)	34	38.5 (27.5,49.4)
Adjusted prevalence difference* (95% CI; q-value)	Referent		3.0 (-3.4,9.3; 0.52)		-14.5 (-25.2,-3.9; 0.04)	
When thinking about democracy in the United States these days, do you believe…						
There is a serious threat to our democracy.	3600	73.8 (72.3,75.2)	146	64.0 (56.3,71.6)	72	49.1 (38.5,59.7)
There may be a threat to our democracy, but it is not serious.	881	20.1 (18.7,21.4)	38	20.4 (14.0,26.8)	25	27.1 (16.7,37.6)
There is no threat to our democracy.	238	5.7 (4.9,6.5)	22	13.3 (7.4,19.2)	20	23.2 (13.6,32.7)
Adjusted prevalence difference* (95% CI; q-value)	Referent		-6.5 (-13.7,0.7; 0.19)		-16.8 (-26.3,-7.3; 0.004)	
How important do you think it is for the United States to remain a democracy?						
Not important	51	1.3 (0.9,1.7)	6	3.4 (0.6,6.3)	6	6.8 (1.2,12.4)
Somewhat important	160	4.3 (3.6,5.0)	26	15.7 (9.6,21.8)	16	18.8 (9.5,28.2)
Very or extremely important	4514	94.0 (93.1,94.8)	177	80.9 (74.4,87.4)	94	73.2 (63.0,83.3)
Adjusted prevalence difference* (95% CI; q-value)	Referent		1.0 (-1.7,3.7; 0.73)		4.0 (-1.5,9.5; 0.41)	
Democracy is the best form of government.						
Do not agree	147	3.7 (3.0,4.4)	14	7.4 (3.4,11.3)	15	14.4 (6.5,22.2)
Somewhat agree	781	18.9 (17.5,20.2)	63	33.8 (26.3,41.2)	20	22.2 (12.5,32.0)
Strongly or very strongly agree	3794	77.0 (75.6,78.4)	130	57.6 (49.9,65.2)	81	61.7 (51.0,72.5)
Adjusted prevalence difference* (95% CI; q-value)	Referent		0.8 (-3.3,5.0; 0.83)		7.1 (-0.3,14.5; 0.25)	
These days, American democracy only serves the interests of the wealthy and powerful.						
Do not agree	1410	26.3 (24.9,27.6)	46	17.2 (12.2,22.3)	27	20.6 (12.2,29.0)
Somewhat agree	1685	35.9 (34.4,37.4)	82	44.1 (36.4,51.8)	34	33.2 (22.8,43.7)
Strongly or very strongly agree	1634	37.6 (36.0,39.2)	80	38.0 (30.6,45.3)	56	45.1 (34.6,55.6)
Adjusted prevalence difference* (95% CI; q-value)	Referent		-3.4 (-11.2,4.4; 0.48)		-0.5 (-12.0,10.9; 0.93)	
Having a strong leader for America is more important than having a democracy.						
Do not agree	3350	68.1 (66.6,69.7)	93	36.7 (29.7,43.7)	39	31.3 (21.7,40.9)
Somewhat agree	762	17.6 (16.3,18.9)	62	31.1 (24.1,38.2)	19	17.7 (9.3,26.0)
Strongly or very strongly agree	607	13.7 (12.6,14.9)	52	30.8 (23.3,38.3)	57	48.8 (38.2,59.5)
Adjusted prevalence difference* (95% CI; q-value)	Referent		12.8 (5.5,20.0; 0.004)		30.8 (20.0,41.6; <0.001)	
The 2020 election was stolen from Donald Trump, and Joe Biden is an illegitimate president.						
Do not agree	3999	84.4 (83.3,85.6)	40	17.8 (12.2,23.3)	24	18.6 (10.5,26.6)
Somewhat agree	342	7.1 (6.3,8.0)	64	32.4 (25.1,39.7)	19	23.1 (13.2,33.0)
Strongly or very strongly agree	382	8.1 (7.2,9.0)	104	49.0 (41.4,56.6)	74	57.7 (47.0,68.4)
Adjusted prevalence difference* (95% CI; q-value)	Referent		38.5 (31.1,45.8; <0.001)		49.9 (39.9,60.0; <0.001)	
Armed citizens should patrol polling places at election time.						
Do not agree	4393	91.8 (90.9,92.7)	116	51.9 (44.3,59.6)	41	29.2 (19.9,38.5)
Somewhat agree	201	4.6 (3.9,5.3)	58	28.6 (21.7,35.5)	24	24.4 (14.8,34.1)
Strongly or very strongly agree	131	3.2 (2.6,3.9)	35	19.4 (13.1,25.8)	51	44.8 (34.2,55.4)
Adjusted prevalence difference* (95% CI; q-value)	Referent		14.2 (8.0,20.5; <0.001)		39.2 (28.8,49.7; <0.001)	
In the next few years, there will be civil war in the United States						
Do not agree	2619	54.0 (52.5,55.6)	57	28.3 (21.2,35.4)	28	21.7 (12.9,30.4)
Somewhat agree	1630	34.4 (32.9,35.9)	97	41.7 (34.4,49.1)	33	29.6 (19.7,39.5)
Strongly or very strongly agree	458	10.8 (9.8,11.9)	54	29.2 (22.2,36.3)	56	47.7 (37.1,58.3)
Adjusted prevalence difference* (95% CI; q-value)	Referent		15.4 (8.3,22.6; <0.001)		32.1 (21.1,43.1; <0.001)	

Table S12, continued.

Statement	Christian Nationalist Movement					
	Do Not Approve		Somewhat Approve		Strongly/Very Strongly Approve	
	Unweighted n	Weighted % (95% CI)	Unweighted n	Weighted % (95% CI)	Unweighted n	Weighted % (95% CI)
Do you believe that things in this country today are…						
Generally headed in the wrong direction	2537	77.5 (75.8,79.1)	487	82.1 (78.1,86.0)	297	79.6 (74.3,84.9)
Generally headed in the right direction	726	22.0 (20.4,23.6)	83	17.6 (13.6,21.5)	58	20.2 (14.9,25.5)
Adjusted prevalence difference* (95% CI; q-value)	Referent		5.7 (1.5,9.9; 0.04)		3.3 (-1.9,8.5; 0.38)	
When thinking about democracy in the United States these days, do you believe…						
There is a serious threat to our democracy.	2579	76.1 (74.4,77.8)	388	64.5 (59.9,69.0)	254	65.2 (59.2,71.3)
There may be a threat to our democracy, but it is not serious.	536	17.7 (16.2,19.3)	131	24.1 (20.1,28.2)	65	21.4 (16.1,26.8)
There is no threat to our democracy.	156	5.8 (4.8,6.8)	50	10.9 (7.6,14.1)	34	12.0 (7.8,16.2)
Adjusted prevalence difference* (95% CI; q-value)	Referent		-11.0 (-15.7,-6.2; <0.001)		-6.4 (-12.2,-0.6; 0.09)	
How important do you think it is for the United States to remain a democracy?						
Not important	37	1.3 (0.8,1.8)	16	3.7 (1.8,5.5)	7	2.9 (0.6,5.2)
Somewhat important	122	4.8 (3.9,5.7)	48	12.4 (8.6,16.2)	35	12.7 (8.3,17.1)
Very or extremely important	3115	93.6 (92.6,94.6)	505	83.5 (79.5,87.6)	313	84.0 (79.2,88.9)
Adjusted prevalence difference* (95% CI; q-value)	Referent		1.9 (0.0,3.7; 0.20)		0.5 (-1.9,3.0; 0.80)	
Democracy is the best form of government.						
Do not agree	132	4.7 (3.8,5.5)	33	7.4 (4.6,10.2)	22	6.9 (3.8,10.0)
Somewhat agree	578	19.9 (18.3,21.5)	134	28.0 (23.6,32.5)	67	23.1 (17.7,28.4)
Strongly or very strongly agree	2567	75.2 (73.5,76.9)	399	63.8 (59.1,68.5)	266	69.9 (64.1,75.7)
Adjusted prevalence difference* (95% CI; q-value)	Referent		1.4 (-1.4,4.2; 0.69)		-1.0 (-4.4,2.4; 0.86)	
These days, American democracy only serves the interests of the wealthy and powerful.						
Do not agree	836	22.3 (20.7,23.8)	164	25.3 (21.4,29.2)	95	23.2 (18.3,28.0)
Somewhat agree	1175	35.3 (33.5,37.2)	228	42.3 (37.7,47.0)	97	28.0 (22.5,33.4)
Strongly or very strongly agree	1267	42.2 (40.3,44.1)	178	32.1 (27.7,36.4)	161	47.7 (41.7,53.8)
Adjusted prevalence difference* (95% CI; q-value)	Referent		-11.0 (-16.0,-6.0; <0.001)		1.7 (-5.0,8.4; 0.94)	
Having a strong leader for America is more important than having a democracy.						
Do not agree	2418	71.0 (69.2,72.8)	245	39.6 (35.0,44.1)	121	31.4 (25.9,36.8)
Somewhat agree	486	16.1 (14.6,17.5)	161	30.4 (26.0,34.7)	83	23.6 (18.5,28.8)
Strongly or very strongly agree	371	12.6 (11.2,13.9)	163	29.6 (25.2,33.9)	148	44.0 (38.0,50.1)
Adjusted prevalence difference* (95% CI; q-value)	Referent		13.1 (8.4,17.7; <0.001)		27.2 (21.0,33.4; <0.001)	
The 2020 election was stolen from Donald Trump, and Joe Biden is an illegitimate president.						
Do not agree	2908	87.8 (86.5,89.1)	258	44.3 (39.7,49.0)	112	32.0 (26.5,37.5)
Somewhat agree	165	5.1 (4.2,5.9)	146	26.9 (22.6,31.2)	61	20.1 (14.9,25.4)
Strongly or very strongly agree	202	6.9 (5.8,7.9)	162	28.1 (24.0,32.2)	181	47.6 (41.5,53.6)
Adjusted prevalence difference* (95% CI; q-value)	Referent		20.1 (15.9,24.3; <0.001)		38.6 (32.7,44.5; <0.001)	
Armed citizens should patrol polling places at election time.						
Do not agree	3102	93.4 (92.4,94.5)	398	66.5 (62.0,71.1)	177	44.8 (38.9,50.7)
Somewhat agree	107	3.8 (3.0,4.5)	107	21.0 (17.1,25.0)	82	25.2 (19.8,30.6)
Strongly or very strongly agree	68	2.6 (1.9,3.3)	62	11.7 (8.6,14.7)	93	29.0 (23.4,34.7)
Adjusted prevalence difference* (95% CI; q-value)	Referent		7.9 (4.7,11.2; <0.001)		23.9 (18.3,29.4; <0.001)	
In the next few years, there will be civil war in the United States						
Do not agree	1822	54.1 (52.2,56.0)	218	35.5 (31.1,39.9)	89	23.7 (18.7,28.7)
Somewhat agree	1127	33.9 (32.1,35.7)	249	43.7 (39.1,48.3)	140	37.7 (32.0,43.5)
Strongly or very strongly agree	316	11.4 (10.0,12.7)	99	19.9 (16.1,23.8)	124	38.0 (32.0,44.0)
Adjusted prevalence difference* (95% CI; q-value)	Referent		5.3 (1.1,9.5; 0.06)		21.1 (15.0,27.3; <0.001)	

Table S12, continued.

Statement	White Supremacy Movement					
	Do Not Approve		Somewhat Approve		Strongly/Very Strongly Approve	
	Unweighted n	Weighted % (95% CI)	Unweighted n	Weighted % (95% CI)	Unweighted n	Weighted % (95% CI)
Do you believe that things in this country today are…						
Generally headed in the wrong direction	5568	81.3 (80.3,82.4)	132	75.7 (67.3,84.2)	48	60.5 (47.1,73.9)
Generally headed in the right direction	1215	18.0 (17.0,19.0)	31	24.3 (15.8,32.7)	24	36.2 (23.0,49.4)
Adjusted prevalence difference* (95% CI; q-value)	Referent		-4.6 (-12.6,3.4; 0.36)		-17.0 (-29.6,-4.3; 0.03)	
When thinking about democracy in the United States these days, do you believe…						
There is a serious threat to our democracy.	5011	70.7 (69.4,71.9)	93	49.2 (40.3,58.1)	41	49.0 (35.9,62.2)
There may be a threat to our democracy, but it is not serious.	1394	22.3 (21.1,23.4)	48	34.1 (25.3,42.9)	20	30.6 (17.7,43.5)
There is no threat to our democracy.	385	6.3 (5.6,7.0)	21	16.0 (8.8,23.1)	12	20.3 (9.2,31.5)
Adjusted prevalence difference* (95% CI; q-value)	Referent		-16.7 (-24.8,-8.6; <0.001)		-15.2 (-27.8,-2.6; 0.06)	
How important do you think it is for the United States to remain a democracy?						
Not important	79	1.4 (1.1,1.8)	4	2.4 (-0.1,4.9)	5	8.6 (0.6,16.7)
Somewhat important	290	5.5 (4.8,6.2)	32	25.1 (16.6,33.6)	7	10.0 (1.6,18.5)
Very or extremely important	6438	92.6 (91.8,93.4)	126	72.2 (63.6,80.8)	60	80.4 (69.4,91.4)
Adjusted prevalence difference* (95% CI; q-value)	Referent		-0.2 (-2.9,2.5; 0.92)		5.6 (-2.3,13.5; 0.35)	
Democracy is the best form of government.						
Do not agree	274	4.8 (4.2,5.4)	7	6.5 (1.1,11.9)	6	6.7 (0.8,12.5)
Somewhat agree	1254	20.8 (19.7,22.0)	61	39.4 (30.6,48.2)	16	29.1 (16.4,41.7)
Strongly or very strongly agree	5269	73.7 (72.5,74.9)	93	52.3 (43.4,61.3)	50	63.7 (50.7,76.6)
Adjusted prevalence difference* (95% CI; q-value)	Referent		-1.6 (-6.8,3.7; 0.74)		-1.8 (-7.4,3.8; 0.74)	
These days, American democracy only serves the interests of the wealthy and powerful.						
Do not agree	2073	27.2 (26.0,28.3)	31	16.2 (9.8,22.7)	17	21.2 (10.2,32.1)
Somewhat agree	2414	35.5 (34.3,36.8)	69	44.8 (35.9,53.8)	21	34.2 (21.1,47.3)
Strongly or very strongly agree	2322	37.0 (35.6,38.3)	61	37.2 (28.6,45.8)	33	41.9 (29.2,54.7)
Adjusted prevalence difference* (95% CI; q-value)	Referent		-5.4 (-14.7,3.9; 0.43)		-2.2 (-16.6,12.2; 0.89)	
Having a strong leader for America is more important than having a democracy.						
Do not agree	4441	62.3 (60.9,63.6)	47	25.0 (17.5,32.4)	19	24.4 (13.2,35.6)
Somewhat agree	1323	21.0 (19.9,22.1)	54	34.1 (25.7,42.5)	9	13.2 (4.2,22.1)
Strongly or very strongly agree	1028	16.0 (15.0,17.1)	60	39.2 (30.4,48.1)	43	60.3 (47.5,73.2)
Adjusted prevalence difference* (95% CI; q-value)	Referent		19.2 (10.1,28.3; <0.001)		41.3 (28.5,54.0; <0.001)	
The 2020 election was stolen from Donald Trump, and Joe Biden is an illegitimate president.						
Do not agree	5058	74.2 (73.1,75.4)	41	24.4 (16.8,32.0)	7	7.9 (1.9,13.9)
Somewhat agree	765	11.2 (10.4,12.1)	45	29.7 (21.3,38.2)	21	37.4 (24.1,50.7)
Strongly or very strongly agree	964	14.0 (13.1,14.9)	74	44.0 (35.2,52.8)	42	50.9 (37.6,64.1)
Adjusted prevalence difference* (95% CI; q-value)	Referent		27.6 (19.0,36.3; <0.001)		39.3 (26.2,52.3; <0.001)	
Armed citizens should patrol polling places at election time.						
Do not agree	6123	88.8 (87.9,89.7)	68	36.1 (27.8,44.4)	16	15.8 (7.1,24.5)
Somewhat agree	423	6.7 (6.0,7.4)	54	34.6 (25.9,43.3)	17	29.7 (17.1,42.3)
Strongly or very strongly agree	248	3.9 (3.4,4.5)	39	27.8 (19.5,36.0)	39	53.9 (40.7,67.1)
Adjusted prevalence difference* (95% CI; q-value)	Referent		20.9 (12.7,29.1; <0.001)		47.5 (34.5,60.5; <0.001)	
In the next few years, there will be civil war in the United States						
Do not agree	3612	51.7 (50.4,53.0)	38	23.5 (15.7,31.2)	14	19.6 (8.6,30.5)
Somewhat agree	2423	35.3 (34.1,36.6)	82	48.5 (39.6,57.4)	15	18.1 (8.4,27.7)
Strongly or very strongly agree	735	12.0 (11.1,12.9)	41	26.3 (18.4,34.2)	43	61.8 (48.9,74.6)
Adjusted prevalence difference* (95% CI; q-value)	Referent		9.8 (1.5,18.1; 0.06)		45.0 (32.3,57.7; <0.001)	

Table S12, continued.

Statement	Militia Movement					
	Do Not Approve		Somewhat Approve		Strongly/Very Strongly Approve	
	Unweighted n	Weighted % (95% CI)	Unweighted n	Weighted % (95% CI)	Unweighted n	Weighted % (95% CI)
Do you believe that things in this country today are…						
Generally headed in the wrong direction	3396	78.5 (77.1,79.9)	341	83.3 (78.4,88.1)	115	75.5 (66.5,84.5)
Generally headed in the right direction	878	20.8 (19.4,22.2)	52	16.3 (11.4,21.1)	26	23.9 (15.0,32.9)
Adjusted prevalence difference* (95% CI; q-value)	Referent		5.0 (0.2,9.8; 0.20)		-1.7 (-10.3,6.8; 0.85)	
When thinking about democracy in the United States these days, do you believe…						
There is a serious threat to our democracy.	3291	74.3 (72.8,75.8)	286	66.7 (61.0,72.5)	97	62.5 (52.9,72.1)
There may be a threat to our democracy, but it is not serious.	768	19.4 (18.0,20.8)	77	22.4 (17.3,27.5)	27	22.5 (13.9,31.1)
There is no threat to our democracy.	222	5.8 (5.0,6.7)	30	10.5 (6.4,14.7)	18	15.0 (7.8,22.2)
Adjusted prevalence difference* (95% CI; q-value)	Referent		-4.6 (-10.1,0.9; 0.25)		-5.2 (-13.9,3.4; 0.35)	
How important do you think it is for the United States to remain a democracy?						
Not important	43	1.2 (0.8,1.6)	12	3.4 (1.4,5.4)	9	7.2 (2.0,12.3)
Somewhat important	135	4.1 (3.4,4.9)	39	13.1 (8.8,17.4)	16	14.8 (6.9,22.6)
Very or extremely important	4114	94.4 (93.6,95.3)	343	83.5 (78.9,88.1)	116	77.6 (68.8,86.3)
Adjusted prevalence difference* (95% CI; q-value)	Referent		1.4 (-0.6,3.4; 0.37)		4.3 (-0.8,9.4; 0.28)	
Democracy is the best form of government.						
Do not agree	145	4.1 (3.3,4.8)	27	7.9 (4.7,11.0)	15	10.6 (4.4,16.7)
Somewhat agree	660	17.4 (16.1,18.8)	90	25.4 (20.2,30.5)	33	26.5 (17.7,35.3)
Strongly or very strongly agree	3481	78.1 (76.7,79.6)	276	66.4 (60.8,71.9)	93	62.6 (53.1,72.1)
Adjusted prevalence difference* (95% CI; q-value)	Referent		2.3 (-0.9,5.6; 0.34)		2.5 (-3.7,8.6; 0.69)	
These days, American democracy only serves the interests of the wealthy and powerful.						
Do not agree	1289	27.1 (25.7,28.6)	102	21.9 (17.5,26.2)	28	17.6 (10.5,24.7)
Somewhat agree	1548	36.2 (34.6,37.8)	146	37.7 (32.1,43.3)	36	30.9 (21.5,40.3)
Strongly or very strongly agree	1455	36.4 (34.8,38.1)	144	39.6 (33.8,45.4)	77	50.4 (40.8,60.0)
Adjusted prevalence difference* (95% CI; q-value)	Referent		-1.0 (-7.0,5.1; 0.95)		5.6 (-4.8,16.0; 0.56)	
Having a strong leader for America is more important than having a democracy.						
Do not agree	2933	65.8 (64.2,67.4)	195	43.8 (38.2,49.5)	55	34.6 (25.8,43.4)
Somewhat agree	695	17.4 (16.1,18.7)	100	27.0 (21.9,32.1)	25	20.5 (12.6,28.5)
Strongly or very strongly agree	658	16.4 (15.1,17.7)	98	28.8 (23.2,34.3)	61	44.0 (34.5,53.6)
Adjusted prevalence difference* (95% CI; q-value)	Referent		9.7 (4.3,15.0; 0.003)		22.3 (12.6,32.0; <0.001)	
The 2020 election was stolen from Donald Trump, and Joe Biden is an illegitimate president.						
Do not agree	3517	81.7 (80.4,83.0)	128	32.1 (26.7,37.4)	26	18.5 (11.0,26.0)
Somewhat agree	337	7.9 (7.0,8.8)	102	27.8 (22.5,33.2)	34	26.7 (17.9,35.4)
Strongly or very strongly agree	429	10.0 (9.0,11.1)	163	39.9 (34.3,45.5)	81	53.7 (44.1,63.3)
Adjusted prevalence difference* (95% CI; q-value)	Referent		27.7 (22.2,33.2; <0.001)		42.2 (32.8,51.6; <0.001)	
Armed citizens should patrol polling places at election time.						
Do not agree	3962	91.5 (90.6,92.5)	252	60.4 (54.7,66.1)	44	27.7 (19.5,35.9)
Somewhat agree	197	4.7 (4.0,5.5)	90	25.7 (20.5,30.9)	39	30.9 (21.8,39.9)
Strongly or very strongly agree	128	3.4 (2.8,4.1)	50	13.6 (9.4,17.8)	59	41.4 (32.0,50.9)
Adjusted prevalence difference* (95% CI; q-value)	Referent		8.1 (4.1,12.0; <0.001)		35.1 (25.7,44.4; <0.001)	
In the next few years, there will be civil war in the United States						
Do not agree	2384	54.3 (52.6,56.0)	122	30.0 (24.8,35.3)	27	19.6 (11.9,27.2)
Somewhat agree	1486	34.5 (33.0,36.1)	180	43.6 (37.9,49.3)	49	33.8 (24.8,42.9)
Strongly or very strongly agree	407	10.6 (9.5,11.7)	88	25.6 (20.3,30.9)	66	46.6 (37.0,56.1)
Adjusted prevalence difference* (95% CI; q-value)	Referent		11.7 (6.5,16.9; <0.001)		29.4 (19.5,39.3; <0.001)	

Table S12, continued.

Statement	Boogaloo Movement					
	Do Not Approve		Somewhat Approve		Strongly/Very Strongly Approve	
	Unweighted n	Weighted % (95% CI)	Unweighted n	Weighted % (95% CI)	Unweighted n	Weighted % (95% CI)
Do you believe that things in this country today are…						
Generally headed in the wrong direction	2050	77.9 (76.1,79.6)	69	67.7 (56.4,79.1)	36	57.0 (42.2,71.8)
Generally headed in the right direction	570	21.6 (19.8,23.4)	28	32.3 (20.9,43.6)	20	39.3 (24.7,54.0)
Adjusted prevalence difference* (95% CI; q-value)	Referent		-11.5 (-22.6,-0.5; 0.14)		-19.0 (-33.2,-4.8; 0.06)	
When thinking about democracy in the United States these days, do you believe…						
There is a serious threat to our democracy.	2082	76.7 (74.8,78.5)	49	45.6 (34.2,56.9)	29	45.4 (31.0,59.8)
There may be a threat to our democracy, but it is not serious.	410	17.0 (15.4,18.7)	28	32.0 (21.0,43.0)	13	21.8 (10.0,33.6)
There is no threat to our democracy.	135	6.0 (4.9,7.1)	19	21.3 (11.1,31.6)	15	32.8 (18.7,46.8)
Adjusted prevalence difference* (95% CI; q-value)	Referent		-23.0 (-34.1,-11.9; <0.001)		-22.5 (-35.7,-9.3; 0.005)	
How important do you think it is for the United States to remain a democracy?						
Not important	35	1.6 (1.0,2.2)	9	8.8 (3.0,14.7)	4	9.5 (0.2,18.9)
Somewhat important	98	4.9 (3.9,6.0)	17	21.3 (10.6,32.0)	11	20.9 (8.2,33.7)
Very or extremely important	2497	93.3 (92.1,94.5)	70	68.8 (57.6,80.1)	41	68.5 (54.3,82.7)
Adjusted prevalence difference* (95% CI; q-value)	Referent		5.4 (-0.4,11.3; 0.39)		5.3 (-4.1,14.7; 0.45)	
Democracy is the best form of government.						
Do not agree	110	5.0 (4.0,6.0)	10	13.5 (4.8,22.1)	4	7.3 (-0.2,14.8)
Somewhat agree	413	18.1 (16.4,19.9)	38	37.2 (26.1,48.3)	10	22.2 (8.7,35.8)
Strongly or very strongly agree	2105	76.7 (74.8,78.6)	48	48.0 (36.5,59.6)	42	68.5 (54.2,82.9)
Adjusted prevalence difference* (95% CI; q-value)	Referent		4.1 (-3.7,11.9; 0.70)		-2.7 (-10.6,5.3; 0.71)	
These days, American democracy only serves the interests of the wealthy and powerful.						
Do not agree	725	24.2 (22.4,25.9)	13	13.3 (5.0,21.5)	8	14.6 (4.0,25.1)
Somewhat agree	954	36.3 (34.3,38.4)	50	52.5 (41.0,64.1)	16	31.6 (17.3,45.8)
Strongly or very strongly agree	952	39.3 (37.2,41.5)	33	32.9 (22.2,43.7)	32	52.0 (37.3,66.6)
Adjusted prevalence difference* (95% CI; q-value)	Referent		-17.0 (-28.5,-5.4; 0.03)		1.1 (-14.8,17.1; 0.92)	
Having a strong leader for America is more important than having a democracy.						
Do not agree	1866	67.3 (65.2,69.4)	33	30.6 (20.1,41.0)	11	19.5 (8.2,30.7)
Somewhat agree	389	16.5 (14.9,18.2)	38	35.5 (24.8,46.2)	9	17.9 (6.1,29.7)
Strongly or very strongly agree	374	15.9 (14.3,17.6)	25	32.6 (21.1,44.1)	35	59.0 (44.6,73.4)
Adjusted prevalence difference* (95% CI; q-value)	Referent		9.5 (-1.9,20.9; 0.18)		36.4 (21.8,51.0; <0.001)	
The 2020 election was stolen from Donald Trump, and Joe Biden is an illegitimate president.						
Do not agree	2208	82.7 (81.1,84.4)	32	31.5 (20.9,42.2)	10	13.9 (4.9,22.8)
Somewhat agree	171	6.9 (5.8,8.0)	42	41.3 (29.9,52.6)	15	34.6 (19.8,49.4)
Strongly or very strongly agree	250	10.2 (8.8,11.6)	22	26.6 (16.1,37.2)	30	47.8 (33.3,62.2)
Adjusted prevalence difference* (95% CI; q-value)	Referent		11.9 (1.7,22.0; 0.10)		35.2 (20.3,50.0; <0.001)	
Armed citizens should patrol polling places at election time.						
Do not agree	2428	90.9 (89.5,92.2)	47	45.8 (34.3,57.4)	10	16.8 (6.1,27.5)
Somewhat agree	104	4.4 (3.5,5.3)	36	36.3 (25.4,47.2)	15	32.6 (18.2,47.1)
Strongly or very strongly agree	97	4.5 (3.5,5.5)	14	17.9 (8.5,27.3)	30	47.5 (33.0,61.9)
Adjusted prevalence difference* (95% CI; q-value)	Referent		8.9 (0.0,17.9; 0.21)		39.4 (25.1,53.8; <0.001)	
In the next few years, there will be civil war in the United States						
Do not agree	1431	52.4 (50.3,54.6)	29	30.4 (19.3,41.5)	7	15.2 (4.1,26.3)
Somewhat agree	909	34.2 (32.2,36.2)	42	40.2 (29.1,51.3)	15	28.7 (14.7,42.7)
Strongly or very strongly agree	285	13.0 (11.4,14.6)	25	28.2 (17.7,38.6)	34	54.2 (39.5,69.0)
Adjusted prevalence difference* (95% CI; q-value)	Referent		6.0 (-5.1,17.0; 0.40)		30.6 (14.8,46.3; 0.001)	

* Prevalence differences are adjusted for age, race and ethnicity, gender, education, income, Census division, and rurality and are for the following responses: item 1, "Generally headed in the wrong direction"; item 2, "There is a serious threat to our democracy"; item 3, "Not important"; item 4, "Do not agree"; all other items, "strongly or very strongly agree." They are expressed in absolute percentage points. Q-values represent the probability that the given difference would be a false discovery; they represent the expected proportion of "false positives" that would be seen among the collection of all differences whose q-values were at or below the given q-value.

Table S13. Approval of individual organizations and movements and justification for violence in non-political situations

What do you think about the use of force or violence in the following situations?	Proud Boys					
	Do Not Approve		Somewhat Approve		Strongly/Very Strongly Approve	
	Unweighted n	Weighted % (95% CI)	Unweighted n	Weighted % (95% CI)	Unweighted n	Weighted % (95% CI)
In self defense						
Never justified	63	1.4 (1.0,1.8)	3	0.8 (-0.1,1.7)	7	4.5 (1.0,8.1)
Sometimes justified	1153	23.7 (22.4,25.1)	53	21.6 (15.8,27.4)	22	18.6 (10.4,26.7)
Usually or always justified	3657	74.6 (73.2,76.0)	268	77.7 (71.8,83.5)	131	75.6 (66.9,84.2)
Adjusted prevalence difference* (95% CI; q-value)	Referent		4.0 (-1.6,9.7; 0.47)		3.9 (-4.0,11.8; 0.55)	
To prevent someone from injuring or killing another person						
Never justified	63	1.6 (1.1,2.0)	4	1.2 (-0.0,2.5)	7	5.1 (1.2,8.9)
Sometimes justified	968	20.7 (19.4,22.0)	43	16.6 (11.6,21.7)	16	14.1 (6.6,21.6)
Usually or always justified	3827	77.2 (75.8,78.6)	277	82.1 (77.0,87.3)	137	79.5 (71.2,87.7)
Adjusted prevalence difference* (95% CI; q-value)	Referent		5.9 (0.6,11.1; 0.12)		6.5 (-1.3,14.4; 0.34)	
To prevent someone from injuring or killing themselves						
Never justified	294	6.5 (5.7,7.3)	14	5.3 (2.0,8.7)	18	11.5 (5.4,17.7)
Sometimes justified	1716	36.3 (34.8,37.8)	100	33.0 (26.9,39.1)	45	26.9 (19.0,34.7)
Usually or always justified	2854	56.8 (55.2,58.4)	209	61.5 (55.2,67.8)	97	60.2 (51.4,69.1)
Adjusted prevalence difference* (95% CI; q-value)	Referent		6.0 (-0.2,12.2; 0.21)		7.5 (-1.7,16.7; 0.29)	
To prevent harm or damage to property						
Never justified	904	18.8 (17.6,20.1)	21	8.0 (4.2,11.7)	14	7.7 (3.4,12.0)
Sometimes justified	2577	53.2 (51.6,54.8)	149	46.3 (40.0,52.6)	48	31.1 (22.6,39.6)
Usually or always justified	1377	27.4 (26.0,28.8)	154	45.7 (39.5,52.0)	98	59.9 (51.0,68.7)
Adjusted prevalence difference* (95% CI; q-value)	Referent		16.4 (10.1,22.7; <0.001)		31.8 (22.9,40.7; <0.001)	
To win an argument						
Never justified	4482	91.2 (90.3,92.2)	247	71.2 (65.1,77.3)	102	54.3 (45.2,63.4)
Sometimes justified	294	6.1 (5.4,6.9)	55	20.0 (14.4,25.6)	27	21.3 (13.4,29.2)
Usually or always justified	91	2.2 (1.7,2.8)	22	8.8 (4.9,12.7)	29	21.5 (13.7,29.3)
Adjusted prevalence difference* (95% CI; q-value)	Referent		4.9 (1.2,8.5; 0.04)		16.9 (9.4,24.4; <0.001)	
In response to an insult						
Never justified	4322	87.0 (85.9,88.1)	238	68.3 (62.2,74.4)	94	50.9 (41.9,59.9)
Sometimes justified	433	10.0 (9.0,11.0)	67	24.3 (18.7,29.9)	28	18.7 (11.8,25.7)
Usually or always justified	112	2.7 (2.1,3.2)	18	7.2 (3.4,11.1)	37	28.3 (19.7,36.9)
Adjusted prevalence difference* (95% CI; q-value)	Referent		2.9 (-0.8,6.7; 0.36)		23.0 (14.6,31.4; <0.001)	
To get respect						
Never justified	4531	91.9 (90.9,92.8)	249	69.9 (63.7,76.2)	104	55.3 (46.2,64.4)
Sometimes justified	245	5.6 (4.9,6.4)	48	19.7 (14.1,25.4)	18	12.6 (6.5,18.7)
Usually or always justified	93	2.2 (1.7,2.7)	26	10.0 (5.8,14.3)	38	30.7 (21.9,39.6)
Adjusted prevalence difference* (95% CI; q-value)	Referent		6.2 (2.0,10.3; 0.02)		25.6 (17.2,34.1; <0.001)	

Table S13, continued.

What do you think about the use of force or violence in the following situations?	Oath Keepers					
	Do Not Approve		Somewhat Approve		Strongly/Very Strongly Approve	
	Unweighted n	Weighted % (95% CI)	Unweighted n	Weighted % (95% CI)	Unweighted n	Weighted % (95% CI)
In self defense						
Never justified	48	1.6 (1.1,2.1)	9	2.9 (1.0,4.8)	7	3.4 (0.7,6.1)
Sometimes justified	896	26.1 (24.4,27.7)	58	22.6 (16.7,28.5)	28	17.9 (11.3,24.6)
Usually or always justified	2496	72.0 (70.3,73.7)	253	74.1 (68.1,80.1)	182	77.6 (70.6,84.6)
Adjusted prevalence difference* (95% CI; q-value)	Referent		4.1 (-1.8,10.1; 0.46)		10.3 (3.6,17.0; 0.02)	
To prevent someone from injuring or killing another person						
Never justified	46	1.6 (1.1,2.1)	6	2.1 (0.4,3.8)	10	4.7 (1.6,7.7)
Sometimes justified	711	21.1 (19.6,22.7)	51	19.1 (13.7,24.5)	22	15.6 (9.1,22.2)
Usually or always justified	2671	76.7 (75.1,78.3)	263	78.5 (72.9,84.0)	183	78.2 (71.1,85.2)
Adjusted prevalence difference* (95% CI; q-value)	Referent		3.7 (-1.9,9.4; 0.45)		7.3 (0.7,13.9; 0.15)	
To prevent someone from injuring or killing themselves						
Never justified	222	7.0 (6.0,7.9)	18	5.2 (2.7,7.8)	17	9.1 (4.2,14.0)
Sometimes justified	1245	36.9 (35.1,38.7)	109	34.6 (28.5,40.6)	47	23.4 (16.7,30.0)
Usually or always justified	1967	55.7 (53.8,57.5)	193	60.0 (53.8,66.2)	152	66.3 (58.8,73.7)
Adjusted prevalence difference* (95% CI; q-value)	Referent		4.3 (-2.2,10.9; 0.43)		14.9 (7.1,22.7; 0.002)	
To prevent harm or damage to property						
Never justified	696	20.1 (18.6,21.6)	27	9.5 (5.8,13.3)	18	9.4 (4.8,14.0)
Sometimes justified	1839	53.3 (51.4,55.1)	152	47.0 (40.7,53.3)	65	30.0 (22.9,37.0)
Usually or always justified	895	26.1 (24.4,27.7)	142	43.5 (37.2,49.8)	132	58.3 (50.7,65.9)
Adjusted prevalence difference* (95% CI; q-value)	Referent		14.1 (7.7,20.5; <0.001)		31.5 (23.8,39.1; <0.001)	
To win an argument						
Never justified	3189	91.6 (90.4,92.7)	241	71.6 (65.6,77.6)	133	54.6 (46.9,62.3)
Sometimes justified	183	5.8 (4.9,6.7)	59	18.9 (14.0,23.8)	40	20.3 (13.7,26.8)
Usually or always justified	64	2.2 (1.6,2.9)	21	9.5 (5.1,13.9)	41	22.7 (16.0,29.4)
Adjusted prevalence difference* (95% CI; q-value)	Referent		4.5 (0.4,8.6; 0.13)		17.4 (11.0,23.8; <0.001)	
In response to an insult						
Never justified	3085	88.0 (86.7,89.3)	235	69.0 (62.9,75.1)	132	55.2 (47.5,62.9)
Sometimes justified	284	9.2 (8.1,10.4)	58	19.2 (14.3,24.0)	48	23.7 (17.2,30.2)
Usually or always justified	65	2.3 (1.7,3.0)	26	11.4 (6.5,16.3)	36	19.9 (13.2,26.6)
Adjusted prevalence difference* (95% CI; q-value)	Referent		6.4 (2.1,10.7; 0.03)		14.1 (7.4,20.7; <0.001)	
To get respect						
Never justified	3221	92.4 (91.3,93.5)	253	73.5 (67.5,79.5)	145	58.6 (50.8,66.4)
Sometimes justified	160	5.3 (4.4,6.2)	41	15.8 (10.9,20.7)	28	14.7 (9.0,20.4)
Usually or always justified	56	1.9 (1.3,2.5)	25	10.2 (5.7,14.7)	43	25.4 (18.2,32.6)
Adjusted prevalence difference* (95% CI; q-value)	Referent		5.5 (1.2,9.7; 0.07)		20.3 (13.6,27.0; <0.001)	

Table S13, continued.

What do you think about the use of force or violence in the following situations?	Three Percenters					
	Do Not Approve		Somewhat Approve		Strongly/Very Strongly Approve	
	Unweighted n	Weighted % (95% CI)	Unweighted n	Weighted % (95% CI)	Unweighted n	Weighted % (95% CI)
In self defense						
Never justified	46	2.2 (1.5,2.9)	7	3.2 (0.8,5.5)	4	4.0 (0.0,8.0)
Sometimes justified	616	25.7 (23.8,27.7)	40	23.8 (16.7,31.0)	22	23.1 (13.7,32.5)
Usually or always justified	1755	71.8 (69.8,73.9)	149	72.4 (65.1,79.8)	91	71.2 (61.3,81.1)
Adjusted prevalence difference* (95% CI; q-value)	Referent		2.8 (-4.8,10.4; 0.68)		5.3 (-4.3,14.9; 0.58)	
To prevent someone from injuring or killing another person						
Never justified	45	2.2 (1.5,2.9)	7	3.7 (0.9,6.6)	4	3.0 (-0.2,6.3)
Sometimes justified	510	21.6 (19.7,23.4)	36	22.3 (15.1,29.5)	22	21.7 (12.7,30.8)
Usually or always justified	1850	75.5 (73.5,77.5)	154	74.0 (66.6,81.4)	90	72.5 (63.0,82.1)
Adjusted prevalence difference* (95% CI; q-value)	Referent		0.7 (-6.8,8.2; 0.91)		3.7 (-5.8,13.2; 0.66)	
To prevent someone from injuring or killing themselves						
Never justified	179	8.2 (6.9,9.6)	7	3.5 (0.8,6.2)	12	10.4 (3.6,17.3)
Sometimes justified	862	36.2 (34.0,38.3)	75	39.5 (31.7,47.4)	34	27.4 (18.3,36.4)
Usually or always justified	1369	55.1 (52.9,57.4)	115	57.0 (49.1,64.8)	71	60.5 (50.4,70.6)
Adjusted prevalence difference* (95% CI; q-value)	Referent		3.9 (-4.4,12.2; 0.69)		10.5 (0.1,20.8; 0.23)	
To prevent harm or damage to property						
Never justified	494	20.5 (18.7,22.3)	25	12.7 (7.7,17.7)	12	9.6 (3.4,15.7)
Sometimes justified	1235	50.2 (47.9,52.4)	82	43.3 (35.4,51.2)	42	36.5 (26.6,46.3)
Usually or always justified	684	28.9 (26.8,31.0)	90	43.9 (36.1,51.7)	62	50.6 (40.4,60.8)
Adjusted prevalence difference* (95% CI; q-value)	Referent		11.3 (3.3,19.4; 0.03)		21.3 (10.9,31.7; <0.001)	
To win an argument						
Never justified	2198	89.2 (87.7,90.8)	134	64.1 (56.3,71.9)	62	43.4 (33.5,53.3)
Sometimes justified	154	6.9 (5.7,8.1)	40	23.3 (16.2,30.4)	25	26.2 (16.6,35.8)
Usually or always justified	62	3.5 (2.5,4.5)	22	12.4 (7.2,17.6)	28	26.1 (17.0,35.2)
Adjusted prevalence difference* (95% CI; q-value)	Referent		4.7 (-0.4,9.9; 0.27)		18.2 (9.4,27.1; 0.002)	
In response to an insult						
Never justified	2131	86.4 (84.7,88.0)	134	62.5 (54.6,70.4)	61	46.1 (36.0,56.2)
Sometimes justified	215	9.8 (8.4,11.2)	39	22.6 (15.8,29.4)	24	22.5 (13.9,31.1)
Usually or always justified	66	3.5 (2.5,4.4)	24	14.9 (8.6,21.2)	32	29.7 (20.1,39.4)
Adjusted prevalence difference* (95% CI; q-value)	Referent		7.6 (1.8,13.4; 0.10)		21.7 (11.8,31.5; <0.001)	
To get respect						
Never justified	2229	90.7 (89.3,92.1)	139	64.8 (56.9,72.7)	61	41.2 (31.4,50.9)
Sometimes justified	128	6.0 (4.9,7.2)	34	20.6 (13.7,27.5)	21	21.9 (13.0,30.8)
Usually or always justified	58	2.9 (2.1,3.8)	23	14.2 (8.2,20.1)	34	34.3 (24.2,44.3)
Adjusted prevalence difference* (95% CI; q-value)	Referent		7.1 (1.3,12.9; 0.09)		27.4 (17.8,37.1; <0.001)	

Table S13, continued.

What do you think about the use of force or violence in the following situations?	QAnon					
	Do Not Approve		Somewhat Approve		Strongly/Very Strongly Approve	
	Unweighted n	Weighted % (95% CI)	Unweighted n	Weighted % (95% CI)	Unweighted n	Weighted % (95% CI)
In self defense						
Never justified	65	1.5 (1.1,1.9)	5	2.8 (0.3,5.2)	5	3.8 (0.2,7.4)
Sometimes justified	1085	22.9 (21.6,24.2)	38	22.4 (15.5,29.3)	19	23.6 (13.1,34.2)
Usually or always justified	3581	75.4 (74.0,76.7)	166	74.8 (67.7,81.9)	93	70.8 (60.1,81.6)
Adjusted prevalence difference* (95% CI; q-value)	Referent		2.3 (-4.4,9.0; 0.63)		-0.1 (-10.3,10.2; 0.99)	
To prevent someone from injuring or killing another person						
Never justified	61	1.4 (1.0,1.8)	2	1.1 (-0.5,2.6)	5	3.1 (0.2,6.1)
Sometimes justified	909	20.0 (18.7,21.3)	36	19.1 (13.1,25.2)	16	16.8 (7.8,25.7)
Usually or always justified	3746	78.1 (76.7,79.5)	171	79.8 (73.6,86.0)	95	77.3 (67.7,86.9)
Adjusted prevalence difference* (95% CI; q-value)	Referent		3.8 (-2.4,10.1; 0.51)		7.1 (-2.3,16.4; 0.42)	
To prevent someone from injuring or killing themselves						
Never justified	274	6.1 (5.3,6.9)	8	3.9 (1.1,6.8)	11	10.0 (3.0,17.1)
Sometimes justified	1715	37.6 (36.0,39.1)	67	34.4 (27.0,41.9)	31	26.6 (17.1,36.1)
Usually or always justified	2733	55.9 (54.3,57.5)	133	61.4 (53.8,68.9)	75	61.7 (51.1,72.2)
Adjusted prevalence difference* (95% CI; q-value)	Referent		6.5 (-1.2,14.1; 0.28)		12.4 (1.7,23.0; 0.17)	
To prevent harm or damage to property						
Never justified	868	18.4 (17.2,19.7)	18	8.7 (4.5,12.9)	13	10.4 (4.1,16.7)
Sometimes justified	2537	54.0 (52.4,55.6)	90	42.7 (35.2,50.3)	39	32.9 (22.9,43.0)
Usually or always justified	1311	27.0 (25.6,28.4)	101	48.5 (40.9,56.2)	65	55.0 (44.4,65.6)
Adjusted prevalence difference* (95% CI; q-value)	Referent		18.7 (10.9,26.4; <0.001)		27.2 (16.7,37.6; <0.001)	
To win an argument						
Never justified	4355	91.2 (90.2,92.2)	156	69.7 (62.3,77.0)	63	44.1 (33.8,54.4)
Sometimes justified	275	6.0 (5.2,6.8)	37	20.6 (14.0,27.2)	24	24.6 (14.6,34.6)
Usually or always justified	96	2.4 (1.9,3.0)	16	9.7 (4.8,14.5)	28	26.9 (17.2,36.5)
Adjusted prevalence difference* (95% CI; q-value)	Referent		4.5 (-0.3,9.2; 0.25)		22.2 (12.8,31.6; <0.001)	
In response to an insult						
Never justified	4196	87.1 (86.0,88.2)	153	68.0 (60.6,75.4)	64	46.8 (36.3,57.3)
Sometimes justified	418	10.0 (9.0,11.0)	33	18.3 (12.2,24.4)	21	19.0 (10.7,27.2)
Usually or always justified	110	2.6 (2.0,3.1)	23	13.7 (8.0,19.4)	32	32.5 (21.9,43.1)
Adjusted prevalence difference* (95% CI; q-value)	Referent		8.4 (3.3,13.6; 0.01)		26.2 (15.9,36.6; <0.001)	
To get respect						
Never justified	4412	92.4 (91.5,93.3)	154	66.7 (59.1,74.3)	65	43.5 (33.3,53.7)
Sometimes justified	228	5.3 (4.6,6.1)	33	20.0 (13.2,26.9)	19	20.4 (11.3,29.4)
Usually or always justified	85	2.0 (1.5,2.4)	22	13.2 (7.8,18.7)	32	33.4 (22.8,44.0)
Adjusted prevalence difference* (95% CI; q-value)	Referent		8.9 (3.6,14.2; 0.01)		29.0 (18.6,39.4; <0.001)	

Table S13, continued.

What do you think about the use of force or violence in the following situations?	Christian Nationalist Movement					
	Do Not Approve		Somewhat Approve		Strongly/Very Strongly Approve	
	Unweighted n	Weighted % (95% CI)	Unweighted n	Weighted % (95% CI)	Unweighted n	Weighted % (95% CI)
In self defense						
Never justified	52	1.7 (1.2,2.2)	13	3.0 (1.3,4.7)	10	3.3 (1.2,5.4)
Sometimes justified	834	25.2 (23.5,26.8)	112	22.6 (18.3,26.9)	50	17.4 (12.2,22.6)
Usually or always justified	2392	72.9 (71.2,74.6)	444	73.7 (69.2,78.2)	296	79.3 (73.9,84.7)
Adjusted prevalence difference* (95% CI; q-value)	Referent		2.8 (-1.8,7.4; 0.46)		7.8 (2.4,13.2; 0.03)	
To prevent someone from injuring or killing another person						
Never justified	50	1.8 (1.2,2.3)	12	2.8 (1.1,4.5)	16	5.7 (2.6,8.8)
Sometimes justified	678	21.1 (19.5,22.6)	108	19.9 (16.1,23.8)	43	14.7 (10.0,19.4)
Usually or always justified	2538	76.7 (75.1,78.3)	449	76.7 (72.6,80.8)	295	79.3 (74.0,84.6)
Adjusted prevalence difference* (95% CI; q-value)	Referent		1.4 (-2.9,5.8; 0.73)		5.0 (-0.3,10.4; 0.21)	
To prevent someone from injuring or killing themselves						
Never justified	229	8.0 (6.9,9.1)	22	4.1 (2.2,5.9)	32	9.6 (5.7,13.5)
Sometimes justified	1235	38.3 (36.5,40.2)	169	30.1 (25.8,34.5)	82	23.2 (18.1,28.4)
Usually or always justified	1806	53.3 (51.4,55.2)	379	65.3 (60.8,69.9)	241	67.0 (61.3,72.8)
Adjusted prevalence difference* (95% CI; q-value)	Referent		10.2 (5.3,15.2; <0.001)		13.5 (7.3,19.7; <0.001)	
To prevent harm or damage to property						
Never justified	696	21.7 (20.1,23.2)	57	10.2 (7.4,13.0)	31	10.6 (6.5,14.7)
Sometimes justified	1760	53.6 (51.7,55.5)	241	43.3 (38.6,48.0)	118	33.7 (28.0,39.4)
Usually or always justified	813	24.3 (22.7,26.0)	271	45.9 (41.3,50.6)	204	54.7 (48.7,60.8)
Adjusted prevalence difference* (95% CI; q-value)	Referent		17.7 (12.7,22.7; <0.001)		26.5 (20.3,32.8; <0.001)	
To win an argument						
Never justified	3063	92.6 (91.5,93.6)	427	70.9 (66.4,75.5)	243	64.3 (58.4,70.2)
Sometimes justified	147	4.9 (4.0,5.8)	107	20.7 (16.7,24.7)	65	19.6 (14.6,24.6)
Usually or always justified	64	2.2 (1.6,2.8)	36	7.9 (5.0,10.8)	47	16.0 (11.3,20.6)
Adjusted prevalence difference* (95% CI; q-value)	Referent		4.2 (1.4,7.0; 0.03)		11.2 (6.5,15.8; <0.001)	
In response to an insult						
Never justified	2924	87.6 (86.3,89.0)	432	69.9 (65.3,74.4)	237	62.3 (56.3,68.3)
Sometimes justified	271	9.3 (8.1,10.5)	101	21.1 (17.1,25.0)	73	22.2 (17.1,27.3)
Usually or always justified	78	2.8 (2.1,3.4)	37	8.6 (5.5,11.7)	44	15.2 (10.5,19.9)
Adjusted prevalence difference* (95% CI; q-value)	Referent		4.2 (1.1,7.3; 0.05)		9.6 (4.9,14.2; 0.002)	
To get respect						
Never justified	3074	93.0 (92.0,94.0)	454	74.0 (69.5,78.5)	255	66.0 (60.0,72.0)
Sometimes justified	139	4.5 (3.7,5.4)	70	15.4 (11.6,19.2)	45	13.9 (9.5,18.3)
Usually or always justified	63	2.2 (1.6,2.8)	44	9.8 (6.6,13.0)	55	19.9 (14.7,25.2)
Adjusted prevalence difference* (95% CI; q-value)	Referent		6.1 (2.9,9.2; 0.002)		14.8 (9.7,19.9; <0.001)	

Table S13, continued.

What do you think about the use of force or violence in the following situations?	White Supremacy Movement					
	Do Not Approve		Somewhat Approve		Strongly/Very Strongly Approve	
	Unweighted n	Weighted % (95% CI)	Unweighted n	Weighted % (95% CI)	Unweighted n	Weighted % (95% CI)
In self defense						
Never justified	90	1.5 (1.1,1.8)	5	3.4 (0.4,6.4)	5	6.2 (0.5,11.9)
Sometimes justified	1428	20.9 (19.8,22.0)	35	24.5 (16.3,32.8)	18	35.5 (21.9,49.2)
Usually or always justified	5298	77.4 (76.2,78.5)	122	70.7 (62.1,79.3)	50	58.3 (44.7,71.8)
Adjusted prevalence difference* (95% CI; q-value)	Referent		-4.4 (-12.7,3.8; 0.61)		-16.3 (-29.3,-3.3; 0.07)	
To prevent someone from injuring or killing another person						
Never justified	91	1.7 (1.3,2.1)	4	2.4 (0.1,4.7)	9	11.4 (3.5,19.2)
Sometimes justified	1226	18.7 (17.6,19.7)	30	20.5 (12.8,28.2)	14	26.2 (13.3,39.1)
Usually or always justified	5475	79.0 (77.9,80.1)	128	75.7 (67.7,83.8)	49	61.9 (48.5,75.2)
Adjusted prevalence difference* (95% CI; q-value)	Referent		0.0 (-8.0,8.0; 1.00)		-12.0 (-25.1,1.0; 0.23)	
To prevent someone from injuring or killing themselves						
Never justified	375	6.0 (5.3,6.6)	11	5.8 (2.1,9.6)	11	15.4 (5.0,25.8)
Sometimes justified	2297	34.4 (33.1,35.7)	52	33.5 (24.9,42.0)	21	30.1 (18.1,42.1)
Usually or always justified	4132	59.2 (57.9,60.5)	99	59.4 (50.5,68.2)	40	53.9 (40.7,67.1)
Adjusted prevalence difference* (95% CI; q-value)	Referent		2.4 (-6.4,11.2; 0.74)		-0.5 (-13.9,13.0; 0.95)	
To prevent harm or damage to property						
Never justified	1116	16.6 (15.6,17.6)	11	6.0 (2.4,9.6)	7	7.8 (1.6,14.0)
Sometimes justified	3522	52.1 (50.7,53.4)	70	40.5 (31.8,49.1)	19	28.3 (15.9,40.7)
Usually or always justified	2162	30.8 (29.6,32.0)	81	52.2 (43.3,61.1)	46	63.3 (50.5,76.2)
Adjusted prevalence difference* (95% CI; q-value)	Referent		19.4 (10.4,28.4; <0.001)		34.0 (21.4,46.6; <0.001)	
To win an argument						
Never justified	6240	90.7 (89.9,91.5)	92	51.3 (42.4,60.3)	29	34.4 (22.4,46.4)
Sometimes justified	431	6.6 (5.9,7.3)	45	30.4 (22.0,38.9)	21	33.6 (20.4,46.8)
Usually or always justified	135	2.3 (1.8,2.7)	25	16.9 (9.9,23.8)	22	31.5 (19.4,43.6)
Adjusted prevalence difference* (95% CI; q-value)	Referent		13.1 (6.2,20.0; 0.003)		27.2 (15.3,39.1; <0.001)	
In response to an insult						
Never justified	6004	86.3 (85.3,87.3)	88	51.4 (42.5,60.4)	31	36.0 (23.8,48.2)
Sometimes justified	644	10.8 (9.9,11.6)	43	24.9 (17.5,32.4)	18	28.2 (16.1,40.4)
Usually or always justified	159	2.6 (2.1,3.0)	30	22.0 (13.8,30.1)	23	35.2 (22.1,48.2)
Adjusted prevalence difference* (95% CI; q-value)	Referent		18.3 (10.3,26.4; <0.001)		30.7 (17.7,43.7; <0.001)	
To get respect						
Never justified	6303	91.4 (90.7,92.2)	90	48.5 (39.6,57.4)	34	39.5 (26.9,52.2)
Sometimes justified	354	5.6 (5.0,6.3)	38	26.0 (17.8,34.2)	12	20.6 (9.5,31.6)
Usually or always justified	151	2.5 (2.1,3.0)	33	23.7 (15.7,31.7)	26	39.3 (26.2,52.4)
Adjusted prevalence difference* (95% CI; q-value)	Referent		19.5 (11.5,27.5; <0.001)		34.3 (21.2,47.4; <0.001)	

Table S13, continued.

What do you think about the use of force or violence in the following situations?	Militia Movement					
	Do Not Approve		Somewhat Approve		Strongly/Very Strongly Approve	
	Unweighted n	Weighted % (95% CI)	Unweighted n	Weighted % (95% CI)	Unweighted n	Weighted % (95% CI)
In self defense						
Never justified	64	1.6 (1.2,2.0)	10	2.9 (1.1,4.8)	5	3.1 (0.2,6.1)
Sometimes justified	1018	23.9 (22.5,25.3)	50	16.4 (11.5,21.3)	24	23.1 (13.9,32.4)
Usually or always justified	3209	74.2 (72.8,75.7)	333	80.1 (74.9,85.3)	113	73.7 (64.4,83.1)
Adjusted prevalence difference* (95% CI; q-value)	Referent		6.2 (1.2,11.2; 0.11)		1.7 (-6.8,10.3; 0.81)	
To prevent someone from injuring or killing another person						
Never justified	60	1.6 (1.2,2.1)	7	2.5 (0.2,4.7)	7	4.9 (1.0,8.8)
Sometimes justified	840	20.3 (18.9,21.6)	51	13.6 (9.7,17.5)	23	18.7 (10.5,27.0)
Usually or always justified	3375	77.5 (76.1,78.9)	334	83.2 (78.7,87.7)	112	76.4 (67.7,85.1)
Adjusted prevalence difference* (95% CI; q-value)	Referent		6.6 (2.0,11.2; 0.03)		2.5 (-5.8,10.7; 0.85)	
To prevent someone from injuring or killing themselves						
Never justified	252	6.3 (5.4,7.1)	25	7.7 (4.1,11.3)	12	8.6 (2.7,14.4)
Sometimes justified	1472	35.3 (33.7,36.9)	140	35.3 (29.7,40.8)	39	27.5 (18.9,36.1)
Usually or always justified	2561	58.1 (56.5,59.8)	228	56.4 (50.6,62.2)	91	63.9 (54.5,73.2)
Adjusted prevalence difference* (95% CI; q-value)	Referent		0.4 (-5.3,6.2; 0.92)		10.3 (0.9,19.7; 0.16)	
To prevent harm or damage to property						
Never justified	796	18.7 (17.4,20.0)	38	10.2 (6.5,13.9)	9	6.0 (1.8,10.2)
Sometimes justified	2227	52.3 (50.6,54.0)	194	48.1 (42.3,53.8)	46	31.2 (22.3,40.2)
Usually or always justified	1259	28.5 (27.1,30.0)	161	41.0 (35.4,46.7)	87	62.7 (53.5,72.0)
Adjusted prevalence difference* (95% CI; q-value)	Referent		11.2 (5.3,17.0; 0.001)		33.1 (24.1,42.1; <0.001)	
To win an argument						
Never justified	3947	91.1 (90.1,92.1)	314	76.2 (70.9,81.4)	88	54.2 (44.5,63.9)
Sometimes justified	250	6.1 (5.2,6.9)	53	15.4 (10.9,19.9)	26	23.9 (15.0,32.8)
Usually or always justified	90	2.5 (1.9,3.0)	24	7.0 (4.0,10.1)	28	21.9 (13.8,29.9)
Adjusted prevalence difference* (95% CI; q-value)	Referent		3.6 (0.5,6.7; 0.17)		16.8 (9.1,24.5; <0.001)	
In response to an insult						
Never justified	3836	87.8 (86.7,89.0)	301	73.5 (68.3,78.8)	82	52.2 (42.6,61.8)
Sometimes justified	347	9.0 (8.0,10.1)	68	19.1 (14.4,23.8)	24	18.2 (10.9,25.5)
Usually or always justified	103	2.8 (2.2,3.4)	24	6.7 (3.7,9.8)	36	29.6 (20.3,38.8)
Adjusted prevalence difference* (95% CI; q-value)	Referent		3.1 (0.1,6.2; 0.27)		23.9 (15.0,32.8; <0.001)	
To get respect						
Never justified	3996	92.1 (91.2,93.1)	321	77.4 (72.2,82.6)	89	54.4 (44.7,64.1)
Sometimes justified	195	5.0 (4.2,5.8)	44	13.4 (9.0,17.8)	16	13.7 (6.8,20.6)
Usually or always justified	96	2.6 (2.0,3.1)	27	8.4 (5.0,11.7)	37	31.9 (22.4,41.3)
Adjusted prevalence difference* (95% CI; q-value)	Referent		4.6 (1.3,8.0; 0.05)		26.1 (17.1,35.0; <0.001)	

Table S13, continued.

What do you think about the use of force or violence in the following situations?	Boogaloo Movement					
	Do Not Approve		Somewhat Approve		Strongly/Very Strongly Approve	
	Unweighted n	Weighted % (95% CI)	Unweighted n	Weighted % (95% CI)	Unweighted n	Weighted % (95% CI)
In self defense						
Never justified	46	2.1 (1.4,2.7)	5	5.3 (0.6,10.0)	2	2.1 (-0.8,4.9)
Sometimes justified	666	25.2 (23.3,27.0)	25	30.1 (18.8,41.4)	13	27.3 (13.5,41.1)
Usually or always justified	1919	72.6 (70.7,74.5)	66	62.5 (50.9,74.1)	42	70.6 (56.8,84.5)
Adjusted prevalence difference* (95% CI; q-value)	Referent		-7.6 (-18.5,3.3; 0.59)		-0.1 (-13.9,13.6; 0.99)	
To prevent someone from injuring or killing another person						
Never justified	45	2.0 (1.4,2.6)	7	7.3 (1.9,12.7)	5	8.6 (0.7,16.4)
Sometimes justified	538	20.8 (19.1,22.6)	20	20.0 (11.0,29.1)	11	24.4 (10.7,38.1)
Usually or always justified	2038	76.7 (74.9,78.5)	69	70.7 (60.3,81.0)	41	67.0 (52.6,81.3)
Adjusted prevalence difference* (95% CI; q-value)	Referent		-2.4 (-13.0,8.2; 0.87)		-5.6 (-19.5,8.4; 0.70)	
To prevent someone from injuring or killing themselves						
Never justified	169	7.0 (5.9,8.2)	12	12.3 (5.1,19.4)	5	6.8 (0.5,13.0)
Sometimes justified	973	37.4 (35.3,39.5)	34	34.5 (23.5,45.6)	17	29.1 (15.6,42.6)
Usually or always justified	1484	55.2 (53.1,57.3)	50	51.2 (39.6,62.7)	35	64.1 (50.1,78.1)
Adjusted prevalence difference* (95% CI; q-value)	Referent		-0.4 (-12.0,11.3; 0.99)		14.1 (0.0,28.1; 0.29)	
To prevent harm or damage to property						
Never justified	532	20.4 (18.7,22.1)	15	14.7 (7.3,22.0)	2	2.1 (-0.8,4.9)
Sometimes justified	1369	51.4 (49.3,53.6)	44	43.2 (31.8,54.6)	19	31.5 (18.0,45.0)
Usually or always justified	725	27.8 (25.9,29.7)	37	40.1 (28.6,51.7)	36	66.4 (52.8,80.1)
Adjusted prevalence difference* (95% CI; q-value)	Referent		9.1 (-2.3,20.5; 0.24)		34.5 (21.0,48.1; <0.001)	
To win an argument						
Never justified	2420	90.9 (89.6,92.2)	56	52.9 (41.2,64.5)	17	30.0 (16.4,43.6)
Sometimes justified	135	5.4 (4.4,6.4)	27	28.9 (18.0,39.8)	14	29.0 (15.1,42.8)
Usually or always justified	74	3.5 (2.6,4.4)	12	14.2 (5.5,23.0)	26	41.0 (27.1,55.0)
Adjusted prevalence difference* (95% CI; q-value)	Referent		8.2 (0.3,16.2; 0.21)		32.4 (18.4,46.3; <0.001)	
In response to an insult						
Never justified	2315	86.0 (84.4,87.6)	58	57.7 (46.2,69.2)	15	26.7 (13.5,39.8)
Sometimes justified	234	10.2 (8.8,11.6)	24	23.9 (14.4,33.4)	14	22.7 (11.1,34.3)
Usually or always justified	80	3.5 (2.7,4.4)	14	16.4 (7.0,25.7)	28	50.7 (36.1,65.2)
Adjusted prevalence difference* (95% CI; q-value)	Referent		10.0 (1.3,18.6; 0.13)		41.4 (27.1,55.7; <0.001)	
To get respect						
Never justified	2431	91.3 (90.0,92.5)	58	52.5 (40.8,64.2)	18	26.7 (14.3,39.1)
Sometimes justified	120	5.1 (4.1,6.1)	25	31.1 (19.8,42.4)	11	24.1 (10.8,37.4)
Usually or always justified	78	3.4 (2.6,4.2)	12	13.6 (5.2,22.1)	28	49.2 (34.7,63.8)
Adjusted prevalence difference* (95% CI; q-value)	Referent		7.9 (0.3,15.5; 0.28)		41.6 (26.8,56.3; <0.001)	

* Prevalence differences are adjusted for age, race and ethnicity, gender, education, income, Census division, and rurality and are for the usually or always justified comparison. They are expressed in absolute percentage points. Q-values represent the probability that the given difference would be a false discovery; they represent the expected proportion of “false positives” that would be seen among the collection of all differences whose q-values were at or below the given q-value.
